# Supplementary material for: Metapopulation Patterns of Iberian Butterflies Revealed by Fuzzy Logic
Source: Insects. 2021 Apr 28;12(5):392. doi: 10.3390/insects12050392 (PMC8145449; doi:10.3390/insects12050392)
Supplement: Supplementary file 1 [file insects-12-00392-s001.zip › insects-1161392-supplementary.pdf]

Table S1. Environmental variables considered. In all cases, variables are the average value for each UTM 10 × 10 cells. Codes of the variables used in the favorability models.

Code	Variables
**Climate**	
PAnn	Total annual precipitation (mm) (1)
PSpr	Total precipitation in spring (1)
PSum	Total precipitation in summer (1)
PAut	Total precipitation in autumn (1)
PWin	Total precipitation in winter (1)
TAnn	Mean annual temperature(ºC) (1)
TSpr	Mean temperature in spring (1)
TSum	Mean temperature in summer (1)
TAut	Mean temperature in autumn (1)
TWin	Mean temperature in winter (1)
TJan	Mean temperature in January (1)
TJul	Mean temperature in July (1)
TnAnn	Minimum annual temperature (1)
TnSpr	Minimum temperature in spring 1)
TnSum	Minimum temperature in summer (1)
TnAut	Minimum temperature in autumn (1)
TnWin	Minimum temperature in winter (1)
TnJan	Minimum temperature in January (1)
TnJul	Minimum temperature in July (1)
TxAnn	Maximum annual temperature (1)
TxSpr	Maximum temperature in spring (1)
TxSum	Maximum temperature in summer (1)
TxAut	Maximum temperature in autumn (1)
TxWin	Maximum temperature in winter (1)
TxJan	Maximum temperature in January (1)
TxJul	Maximum temperature in July (1)
DP01	Days with precipitation > 0.1 mm (1)
DP01Spr	Days with precipitation > 0.1mm in spring (1)
DP01Sum	Days with precipitation > 0.1 mm in summer (1)
DP01Aut	Days with precipitation > 0.1mm in autumn (1)
DP01Win	Days with precipitation > 0.1 mm in winter (1)
DP10	Days with precipitation > 10 mm annual (1)
DP10Spr	Days with precipitation > 10 mm in spring (1)
DP10Sum	Days with precipitation > 10 mm in summer (1)
DP10Aut	Days with precipitation > 10 mm in autumn (1)
DP10Win	Days with precipitation > 10 mm in winter (1)
DP1	Days with precipitation > 1 mm annual (1)
DP1Spr	Days with precipitation > 1 mm in winter (1)
DP1Sum	Days with precipitation ≥ 1 mm in spring (1)
DP1Aut	Days with precipitation ≥ 1 mm in summer 1)
DP1Win	Days with precipitation ≥ 1 mm in autumn (1)
DP30	Days with precipitation ≥ 30 mm annual (1)
DP30Spr	Days with precipitation ≥ 30 mm in spring (1)
DP30Sum	Days with precipitation ≥ 30 mm in summer (1)

DP30Aut	Days with precipitation ≥ 30 mm in autumn (1)
DP30Win	Days with precipitation ≥ 30 mm in winter 1)
DTn0	Days with minimum temperature ≤ 0 ºC (1)
DTn0Spr	Days with minimum temperature ≤ 0 ºC in spring (1)
DTn0Sum	Days with minimum temperature ≤ 0 ºC in summer (1)
DTn0Aut	Days with minimum temperature ≤ 0 ºC in spring (1)
DTn0Win	Days with minimum temperature ≤ 0 ºC in winter (1)
DTn10	Days with minimum temperature ≥ 10 ºC (1)
DTn10Aut	Days with minimum temperature ≥ 10 ºC in autumn (1)
DTx15	Days with maximum temperature ≥ 15ºC (1)
DTx15Spr	Days with maximum temperature ≥ 15ºC in spring (1)
DTx15Sum	Days with maximum temperature ≥ 15ºC in summer (1)
DTx15Aut	Days with maximum temperature ≥ 15ºC in autumn (1)
SID	Direct irradiance at surface [1983-2005] (Kwh/m2/day) (1)
SIDSpr	Direct irradiance at surface in spring [1983-2005] (Kwh/m2/day) (1)
SIDSum	Direct irradiance at surface in summer [1983-2005] (Kwh/m2/day) (1)
SIDAut	Direct irradiance at surface in autumn [1983-2005] (Kwh/m2/day) (1)
SIDWin	Direct irradiance at surface in winter [1983-2005] (Kwh/m2/day) (1)
SIS	Surface Incoming Radiation [1983-2005] (Kwh/m2/day) (1)
SISSpr	Surface Incoming Radiation in spring [1983-2005] (Kwh/m2/day) (1)
SISSum	Surface Incoming Radiation in summer [1983-2005] (Kwh/m2/day) (1)
SISAut	Surface Incoming Radiation in autumn [1983-2005] (Kwh/m2/day) (1)
SISWin	Surface Incoming Radiation in winter [1983-2005] (Kwh/m2/day) (1)
PET	Mean annual potential evapotranspiration (mm) (1)
AET	Mean annual actual evapotranspiration (mm) (1)
**Topography**	
E	Mean elevation (m) (2)
ER	Elevation range (m) (2)
SE	Southward exposure degree (3)
WE	Westward exposure degree (3)
Slop	Slope (º) (calculated from elevation) (2)
CTI	Hydrologically conditioned compound topographic index (3)
**Lithology**	
Clay	Presence of clay (4)
PClay	Proportion of clay (4)
Sil	Presence of siliceous rocks (4)
PSil	Proportion of siliceous rocks (4)
Calc	Presence of calcareous rocks (4)
PCalc	Proportion of calcareous rocks (4)
Grav	Presence of gravel (4)
PGrav	Proportion of gravel (4)
Gyp	Presence of gypsum (4)
PGyp	Proportion of gypsum (4)
**Human activity**	
Dhi	Distance to the nearest highway (km) (5)
HPd	Human population density in 2000 (number of inhabitants km2) (6)
U100	Distance to the nearest town with more than 100,000 inhabitants (km) (5)
U500	Distance to the nearest town with more than 500,000 inhabitants (km) (5)

Spatial situation	
LONG	Longitude
LAT	Latitude

1) [31]; 2) [43] (1996); 3) [44]; 4) [30]; 5) [45]; 6) [46].

Table S2. Logit equations of the favorability functions obtained for each butterfly species in the Iberian Peninsula, which correspond to the type of equation (5) in [14]. Codes of variables as in Table S1.

**Species**	**Logit equations**
Aglais io	Y=8.58505 - 11.521867*SISAUT + 0.001939*AR - 0.639905*TXSPR + 2.682574*TSUM + 0.000813*HPD - 0.030485*DHI + 0.004999*AET + 5.578557*SIDAUT - 0.92369*GYP + 0.561894*DP30SPR + 0.212108*DTN0AUT + 0.250581*DP01SPR - 0.126839*DTN0SPR - 0.396477*TNSPR - 0.021345*DTX25 - 0.006646*U100 + 1.298485*SIDSUM - 0.241855*DP1SPR + 0.255476*DP1SUM - 0.179388*DP01SUM + 0.021357*PSPR - 0.00687*PANN - 1.553634*TJUL + 0.057074*DTX25SPR
Aglais urticae	Y=9.301473 - 2.430828*TXANN - 1.557167*SISSPR + 4.4607*TSUM - 0.013446*U100 - 2.62687*TNAUT + 0.002013*AR - 5.573159*SISWIN + 0.000593*HPD + 0.577104*DP01SPR + 0.01206*PSPR - 0.036347*DTX25 - 0.162877*DP01SUM - 0.012251*PWIN - 0.324236*DP1SPR - 0.08533*DP01 + 0.424995*DTN20AUT + 0.032147*DTX25SUM - 2.78263*TJUL + 0.482183*DP30SPR + 0.001957*AET - 0.070105*DTN20 + 0.877914*DTN0SUM - 0.054925*DTN0SPR + 0.414728*PSIL + 0.482195*TJAN + 3.250891*SIDAUT + 0.007302*PAUT + 2.630727*TAUT
Agriades glandon	Y= - 12.363111 + 0.311484*SLOP - 0.020073*PET + 0.075757*U100 + 0.037149*PAUT + 1.500857*LONG
Agriades pyrenaicus	Y= - 794.678629 - 2.331355*TJUL + 2.745778*CALC + 18.09055*LAT - 0.074306*PET + 0.543006*SLOP + 0.004288*AR + 0.020029*PAUT + 4.785017*CTI
Agriades zullichi	Y=71.905236 - 4.873962*CTI
Anthocharis euphenoides	Y= - 25.357495 + 0.302382*TXWIN - 0.004889*U500 + 0.001402*AR - 0.010694*U100 + 0.003533*AET + 0.000502*HPD + 0.249035*DTN20AUT + 0.488267*CALC - 2.459291*SIDAUT + 0.688171*DP30SPR + 0.216421*DTN0AUT - 0.108395*DTN0SPR - 0.04235*DTX25SPR + 1.512402*TSUM + 0.298713*DP01SPR - 0.07922*DP1 + 0.132326*SLOP - 0.343446*DP01SUM - 0.467732*TXAUT - 0.490455*PSIL + 0.333687*LAT - 0.373309*DP10SPR + 0.260782*DP1SUM - 1.28019*TJUL - 0.044825*DTN20 + 0.576241*DTN0SUM + 0.012783*PSPR - 0.00885*WE - 0.005276*PWIN + 0.549914*TNJUL - 0.605643*TNSPR + 0.017708*DTX25SUM - 0.10788*LONG + 1.55954*SISSUM
Antocharis cardamines	Y=31.015648 + 5.500663*SISSPR - 0.003741*U500 + 0.004901*AET + 0.000671*HPD - 0.551283*DP30WIN + 0.014882*PSPR + 4.347338*SIDSUM - 5.976525*SISSUM - 0.006816*PWIN + 0.39332*DTN20AUT - 1.896433*TNAUT - 0.007443*U100 + 0.195356*SLOP + 0.489141*CALC + 0.657001*DP01SPR + 0.310839*DP30 - 0.055513*DTN20 - 17.721861*SISAUT + 12.828401*SIDAUT - 3.2009*SIDSPR - 0.142267*DP10AUT + 0.038731*DTX25SUM - 0.031545*DTX25 - 1.079117*TXSPR - 0.39104*PCALC - 0.89782*TNWIN + 8.114284*TANN + 0.356073*DP01WIN - 0.255353*DP01 - 0.362618*DP1SPR + 0.294028*GRAV + 0.167921*DP1AUT - 0.227106*CTI - 3.110842*TXANN - 1.304328*TNSPR - 0.133678*DTN0SPR + 0.191311*DTN0AUT
Apatura ilia	Y=16.546046 - 1.299593*PCALC - 0.014953*U500 + 0.404902*LONG + 1.962195*PSIL + 1.683502*DP30WIN - 0.999813*DP30 + 0.000686*HPD - 0.088415*DTN20 + 3.803429*SIDWIN + 0.374341*DTN0AUT + 1.226404*GRAV + 0.135966*SLOP - 0.940257*DP10SPR - 1.11787*DP10AUT +

0.478372*DP10 + 2.943695*TNAUT - 4.053285*TXJUL + 0.17652*DTX25SUM - 0.331371*DTX25AUT + 8.225286*TANN - 4.969531*SISSPR + 1.047086*DP30AUT - 1.239423*DTN0SUM - 8.667277*TNANN + 0.009004*PAUT - 1.654286*TXJAN - 0.155087*DTN0SPR + 2.852938*TJUL

Apatura iris Y= - 16.250191 - 19.156999*SISSPR - 1.971411*TXWIN + 0.001748*AR - 0.508791*DTX25SPR + 0.062833*DTX25 + 0.587414*SIL + 0.175721*LONG - 0.319908*DTN20 + 3.921891*TJAN - 2.451504*TNJAN + 0.784349*TNSPR + 0.956816*LAT + 15.989684*SIDSPR - 24.04534*SIDWIN + 21.501181*SISWIN

Aphantopus hyperanthus Y=57.408227 - 15.777417*SISSPR + 5.108875*SID + 0.759946*DP01SPR - 11.88357*SISAUT - 0.009625*U500 - 0.172001*DP01 + 0.723917*SIL + 8.944092*SIDSPR + 0.775095*DTN20AUT - 0.141569*DTN20 - 1.94676*TNJAN + 2.550178*TNSPR - 1.483009*TSPR + 1.068231*CTI + 0.001291*AR - 0.577551*CALC - 0.355785*DP1SPR - 0.957151*DP30WIN + 0.429948*DP30 + 5.10136*SISWIN - 1.048108*TNJUL + 1.229384*TNAUT

Aporia crataegi Y= - 16.267569 - 0.992686*TXWIN + 0.004488*AET + 0.261398*LONG + 0.000631*HPD - 0.020278*DHI + 0.001574*AR + 0.889275*TXAUT - 0.097078*DTX25SPR + 0.003917*ALT - 0.019029*WE + 16.611766*SIDAUT - 14.873801*SISAUT + 0.476625*CLAY + 0.456749*DP01SPR - 0.318889*DP01SUM + 0.009511*SE + 0.003563*PET + 0.632444*LAT + 0.006521*PSPR - 0.436066*DP1SPR + 0.248782*DTN0AUT - 0.245209*DTN0SPR + 0.082012*DTN20AUT - 0.657869*DP30WIN + 0.253153*DP1WIN - 0.177139*DP01WIN + 0.357368*DP30 - 2.95847*SID + 0.771275*TJAN + 0.035719*DTN0 - 0.375022*TSPR - 0.27112*DP10AUT

Araschnia levana Y= - 24.93953 + 1.997493*LONG + 0.010501*AET - 0.226762*DTN20 + 2.023548*TNSPR - 1.296801*TWIN + 0.456751*DP1SPR + 0.001687*AR - 0.372071*DP1SUM + 1.059645*DP10SUM

Arethusana arethusa Y= - 7.526674 + 0.608523*CALC + 0.006818*AET + 0.988627*DP30SUM - 0.432338*DP1WIN - 0.086142*DTX25SPR + 0.054658*DTX25SUM - 0.115861*DTN0WIN + 0.002223*AR + 0.344056*DP01WIN - 0.675543*DP10AUT - 0.179477*SLOP + 0.258819*DP10SPR + 16.735948*SISSUM - 2.457255*TNAUT + 6.625821*TNSUM - 4.587478*TNJUL - 0.710418*LONG - 1.323067*TXSPR - 33.265146*SISAUT + 0.806321*TXANN - 17.099171*SIDSUM - 0.005574*U500 + 24.282518*SIDAUT - 0.089523*DTN20 + 0.482151*DP1AUT - 0.288636*DP01AUT + 10.537065*SID + 0.445748*DP30 - 0.54026*DP30WIN

Argynnis adippe Y=27.571575 - 0.45505*CTI - 0.005225*U500 + 0.004707*AET - 0.251584*TXWIN - 0.005785*PET - 0.020021*DHI + 0.241038*LONG + 0.014725*PSPR - 0.01285*PSUM - 1.1001*DP30WIN - 0.117879*DTX25SPR + 0.001681*AR + 0.01024*U100 + 0.031315*DTX25SUM + 0.776232*DP01SPR - 2.159646*SIDSUM - 0.145991*DTN20AUT + 0.500885*DP30 - 3.722953*SISSPR - 0.479989*DP1SPR + 0.283566*DP1WIN - 0.192456*DP01 + 0.205066*DTN0AUT - 0.129788*DTN0SPR - 21.647132*SISAUT + 13.846363*SIDAUT + 12.406591*SIS - 0.496644*DP30AUT

Argynnis aglaja Y= - 31.792145 + 0.726913*TXAUT + 0.367757*LONG + 0.003707*AET - 10.788231*SISWIN - 0.627813*TXWIN + 0.002203*AR + 7.796546*SIDWIN - 0.375241*DP1SUM + 0.003058*ALT - 0.581303*DTN20AUT + 0.206604*DP01SPR + 0.718536*Y + 1.186623*DP30SPR + 0.988471*DP30AUT - 0.149214*DTX25SPR + 0.03898*DTX25SUM - 0.016237*DHI + 0.058807*DTN20 + 0.418146*DP10SUM - 0.643835*DP30 + 0.004457*PWIN - 0.107399*DP01AUT

Argynnis niobe Y=72.561919 + 0.002034*ALT + 0.455472*PSIL - 0.627835*DP10SUM - 1.824501*TXWIN + 0.00138*AR + 0.06682*DTX25SUM + 0.015594*PAUT + 12.150694*SID - 28.427343*SISAUT - 0.047711*DTN20 + 0.213599*DP01SPR - 0.196765*DP01AUT + 1.458956*TXJAN + 0.184829*DTN0AUT - 0.044425*DTN0 + 12.879597*SIDAUT - 0.00698*U100 - 0.006698*PWIN + 0.127033*SLOP - 0.027846*DTX25 - 3.065144*TNAUT + 4.186261*TNANN - 1.312505*TNSPR - 2.81591*TXJUL + 2.976344*TXSUM + 0.476262*DP30SPR - 0.108363*DP10 - 0.903141*LAT + 0.015629*PSUM

Argynnis pandora Y=14.482122 - 0.511351*TXWIN + 0.001769*AR - 0.323298*DP10SUM + 0.001903*AET + 0.000689*HPD + 4.566878*TANN - 5.676569*SISAUT + 1.42979*TSUM - 0.57628*GYP + 7.23301*SID + 0.232691*GRAV - 4.275571*SIS + 0.148161*DTN0AUT - 0.098583*DTN0SPR + 0.269985*DTN20AUT - 1.548666*TJUL - 0.045405*DTN20 - 1.675972*TSPR + 0.548706*DP01SPR - 0.128748*DP01 - 0.195768*DP1SPR + 0.005299*PSPR - 7.408204*TAUT + 3.219502*TXAUT + 2.440989*TNAUT + 0.058405*DTX25SPR - 0.54879*TXSPR - 0.037551*DTX25AUT

Argynnis paphia Y= - 26.803536 + 0.006894*AET - 0.004346*U500 + 0.452324*LAT + 0.002078*AR - 0.324555*DP1SUM - 0.227407*DTN20AUT + 0.782071*TSUM - 0.160303*DTX25SPR - 0.48773*DP30WIN + 0.72859*DP30SPR + 0.040722*DTX25SUM - 0.028333*DHI + 0.051745*DTN0AUT - 16.35731*SISSPR - 0.325002*GRAV + 55.85819*SIS - 10.974459*SIDSUM + 0.275798*DP01SPR - 0.224319*DP01AUT + 0.019706*PSPR - 45.697422*SISAUT + 25.480171*SIDAUT - 8.02808*SIDWIN - 0.004328*PANN - 1.701787*TNAUT + 1.417279*TNWIN + 0.50249*DP30SUM + 4.567244*TNSUM - 3.429047*TNJUL + 0.14359*DP1AUT - 1.365282*TANN

Aricia cramera Y=48.848376 + 5.104827*SIDSUM + 0.00196*AET + 0.001615*AR - 5.430737*SISSUM + 0.000752*HPD - 0.06625*DP10WIN - 15.487539*SISAUT + 0.232018*CLAY + 0.217198*CALC + 0.007323*SE - 0.027348*DTX25SPR - 0.668565*LAT + 9.917149*SIDAUT + 5.356075*SISSPR - 2.873065*SIDSPR + 1.939636*TXJUL - 0.329863*TXANN + 2.9446*TSUM - 2.595965*TJUL - 1.907429*TXSUM - 0.002565*U100 + 0.225173*SIL + 0.382479*PCLAY

Aricia montensis Y=18.006989 + 0.002121*AR + 0.419357*CALC + 0.005071*AET - 0.031606*DHI - 0.356872*DP1SUM - 0.09982*DTX25SPR + 0.179578*DTN0AUT + 4.546841*TNSUM - 1.352427*TNAUT - 3.195107*TNJUL + 0.170347*DP01SPR - 0.240395*DP01AUT + 0.516299*DP30SUM - 12.015775*SISAUT + 0.137843*DP01WIN + 0.533458*DP30SPR + 0.01799*PSPR - 0.007047*PWIN - 0.375478*DP10SPR + 2.089818*TNJAN - 1.940029*TNWIN - 0.091445*DTN0SPR - 0.018232*PSUM + 0.38164*DP10SUM + 0.002177*ALT + 7.057*SIDAUT + 0.122281*LONG

Aricia morronensis Y=33.675836 - 1.580316*TXSPR - 0.242071*DP1SUM - 0.021044*U100 + 1.351078*CALC - 0.641995*CTI - 0.630866*DP10AUT - 3.310723*SISSPR + 3.504768*TXSUM - 0.029913*WE + 0.001018*AR + 0.007452*U500 + 0.266421*DP30 - 2.530473*TXJUL

Aricia nicias Y= - 310.719328 - 10.38214*DP10SPR + 5.005388*DP1SPR + 44.329256*SISSPR + 1.175543*SLOP + 4.395139*DP01SUM - 3.065793*DP01SPR

Boloria daphne Y=39.437704 + 0.005712*AET + 0.517808*LONG - 7.800866*SISSPR - 0.624287*DP1SUM + 0.001588*AR - 34.355113*SISAUT - 0.010141*PET + 22.896582*SIDAUT - 0.012631*DHI + 0.085516*DTX25SUM - 1.026356*DP10WIN + 0.009393*PSPR + 15.289996*SIS + 0.084611*DP1 + 0.867639*DP30AUT - 0.16002*DTX25AUT - 0.74396*DP10AUT + 0.522674*DP10 - 0.430638*DP10SUM + 0.126595*SLOP - 0.614209*DP01AUT + 0.162517*DP01 - 0.259707*DP30

Boloria dia Y= - 88.099439 + 2.170448*LAT + 0.301084*LONG + 0.002193*AR + 0.006421*AET - 1.416275*TXJAN - 0.181185*DP1SUM - 0.37809*DP30WIN - 0.01011*U100 + 0.985306*SIL - 0.010674*PET + 0.801589*PCLAY + 0.084249*DTX25SUM - 0.127087*DTX25SPR + 0.888827*DP30SUM - 1.960645*SISSPR - 0.005767*U500 - 0.182637*DTN0WIN - 0.123456*DP01AUT - 0.107052*DTX25AUT - 1.034649*TNSPR + 2.345206*TJAN - 2.005267*TNWIN - 0.428638*DTN0SPR + 0.228535*DTN0 + 2.180527*TNAUT + 0.145662*DP01WIN

Boloria eunomia Y=149.592203 - 0.166841*PET + 1.373684*DTX25AUT - 40.507552*TWIN - 1.101089*SLOP + 7.811498*DP30SUM - 37.932536*TSUM + 73.850568*TANN - 2.399121*DTX25SPR + 1.058359*LONG

Boloria euphrosyne Y= - 17.210374 - 0.99983*TXANN + 1.008177*LAT + 0.238102*LONG + 3.258692*TXSUM - 6.308758*SISSPR + 22.985729*SIDAUT + 0.008009*AET + 0.233569*SLOP + 0.541929*DP01SPR - 0.188925*DTX25SPR - 0.147438*DP01 + 0.91971*DP30SUM - 0.022956*DHI - 0.011292*PSPR - 12.266538*SISAUT - 4.836402*SIDWIN - 2.493707*TXJUL - 0.534353*DTN20AUT

Boloria napaea Y=35.666404 + 1.781938*LONG - 9.094181*SISSPR

Boloria pales Y= - 193.167988 + 0.002485*AR + 5.064187*LAT + 1.811163*DP30AUT - 0.419094*DP1AUT - 4.666404*SISSPR - 1.252216*DP30SPR - 0.717446*TNWIN

Boloria selene Y=3.667039 - 13.093606*SISSPR - 2.047335*TXSPR - 0.012012*U100 + 0.013062*AET - 0.017703*PET + 3.658642*TSUM - 0.343962*DTN20AUT + 0.001263*AR + 0.626343*LAT - 1.247486*TXAUT - 3.033087*TXJAN + 5.402911*TXWIN + 6.270537*TJUL - 5.066669*TNJUL - 3.815668*TXJUL + 0.10281*DP01SPR - 0.220988*DP1AUT + 1.072172*PSIL + 1.329455*TNSPR - 1.550809*TJAN - 0.120185*SLOP + 10.948568*SIDSPR - 16.749592*SIDWIN + 14.200943*SISWIN + 0.643511*DP30AUT - 0.149482*DP30 + 0.850443*PCLAY

Borbo borbonica Y= - 14.579519 + 1.623328*DP30WIN - 0.269298*U100 + 0.656571*DTN20AUT

Brenthis hecate Y=12.518191 - 0.49509*TWIN + 0.006656*AET - 0.773824*PSIL - 0.015596*PSUM + 0.439373*LONG + 1.697031*SISSUM - 0.456089*DTN20AUT - 0.555268*CTI - 0.469017*DP30WIN + 0.626196*DP30SPR - 0.006316*PET - 17.108106*SISWIN + 0.001257*AR + 0.871908*TXAUT - 0.530385*TXSPR + 0.012281*U100 - 0.023099*DHI + 0.528454*DP01SPR - 0.716239*DP01SUM + 12.830063*SIDWIN + 0.618294*DP1SUM - 0.447397*DP1SPR - 0.366007*DP10AUT + 0.404285*DP10SPR

Brenthis ino Y= - 40.339264 - 0.218695*TXWIN + 0.571592*LONG - 0.245086*DP01SUM + 0.534378*DP01SPR + 0.001423*AR + 1.063157*LAT - 0.028058*PSUM + 0.628515*DP10SUM - 0.040829*DHI - 0.01546*PET + 0.006481*AET - 0.102189*DP01 + 1.20695*DP30SPR - 0.220331*DTX25SPR + 0.048284*DTX25SUM + 1.114872*PCALC + 1.897163*PGRAV + 0.006585*PWIN - 0.536289*DTN20AUT - 0.831128*DP30 + 0.729597*DP30SUM + 0.844549*DP30WIN

Cyaniris semiargus Y=42.876361 - 0.502025*TXWIN - 0.005824*U500 + 0.004202*AET + 11.485555*SISSUM - 0.030095*DHI + 0.001814*AR + 0.35045*DP01SPR - 0.317078*DP01SUM - 1.626508*TNJUL + 0.394834*CTI + 0.511748*DP10SUM - 0.627187*PSIL - 0.265072*DP1SPR + 0.011436*PSPR -

0.010377*PWIN + 2.029495*TNSUM - 1.62078*TNAUT + 2.077066*TNSPR - 30.041443*SISAUT + 22.508923*SIDAUT - 8.797817*SIDSUM - 0.854996*LAT + 0.972559*TXAUT - 1.632553*TSPR

Cacyreus marshalli Y= - 48.925822 + 0.001484*HPD + 0.011652*U500 + 1.279112*TNJUL + 0.747765*LAT + 0.003882*AET - 0.032369*DHI + 0.654463*SIL + 0.681916*PGRAV + 0.19011*DP30 - 0.471684*DP10AUT + 0.00682*PAUT - 1.385438*TXSPR + 3.234979*TANN + 0.848016*DTN0SUM - 1.595874*TNSUM - 1.75835*TJAN + 0.678443*TXJAN

Callophrys avis Y=14.138876 + 0.005996*AET + 1.746001*SIL - 0.004698*U500 - 1.119723*CTI + 2.188651*DP30SUM - 0.045114*PSUM + 0.000525*HPD - 1.291594*PCALC - 0.123614*DTX25SPR - 0.00243*ALT + 0.004596*PANN - 0.426601*DTN0SPR + 0.131458*DTN0 + 0.48325*DP30 - 0.420909*DP10WIN - 1.04511*DP30AUT

Callophrys rubi Y=19.467978 - 0.538531*CTI - 2.968731*TXJAN + 0.354183*SIL - 0.004424*U500 + 2.563492*TXWIN + 0.000604*HPD + 0.30811*DP1SUM - 3.036853*SISAUT + 0.001729*AR + 3.016359*SIDSUM - 0.1054*DP10WIN + 0.203213*DP10SUM - 0.44942*TXSPR + 0.046899*DTX25SUM - 0.016815*DTX25 + 0.163938*DTN0AUT - 0.097826*DTN0SPR + 0.309539*DP30AUT - 0.30997*DP01SUM - 0.010667*PWIN + 0.004146*PANN - 0.249574*DP1 + 0.270026*DP01SPR - 2.753839*SISSUM + 0.438686*TNAUT + 0.00401*U100 + 0.180798*DTN20AUT - 0.039726*DTN20 + 0.002772*PET - 0.098961*SLOP + 0.622864*PGRAV + 0.362609*DP1WIN + 0.181107*DP1AUT + 0.196907*CALC - 1.001534*TNWIN + 1.320607*TJAN

Carcharodus alceae Y= - 3.65391 + 0.001243*HPD + 0.001492*AR + 0.002068*AET + 0.833792*TNJUL - 0.659864*TNSUM - 14.143983*SISWIN + 1.072719*DTN0SUM - 0.018231*DHI + 0.004619*U100 + 0.281319*SIL + 4.793667*SISAUT + 8.364298*SIDWIN - 0.245056*DP10WIN + 0.162194*DTN20AUT + 0.366777*PSIL + 0.261605*DP01WIN + 0.172503*DP10AUT + 0.351414*DP30WIN - 0.015336*PWIN + 0.005274*PANN - 0.030312*DTN20 - 0.125347*DP01 + 0.171238*DP01SPR

Carcharodus baeticus Y=54.405897 + 0.101539*DTX25SUM + 0.001614*AR + 0.000944*HPD + 14.0988*SIDWIN + 8.628582*SIDSUM + 0.409982*LONG + 0.194228*DTN20AUT - 0.007407*PET + 0.075418*DP01WIN - 9.612299*SISSUM - 0.255356*TXANN - 0.950183*LAT - 18.610713*SISWIN + 0.074611*DTX25SPR - 0.083439*DTX25AUT + 10.798054*SISSPR - 7.413761*SIDSPR

Carcharodus flocciferus Y=18.609995 - 0.192461*TXAUT + 0.524466*LONG - 7.654717*SISSPR + 4.909093*SIS + 0.000659*AR + 0.216891*DP01SPR + 0.002392*AET - 0.210185*DP01SUM - 0.055572*DP1

Carcharodus lavatherae Y= - 37.432427 - 0.718557*TXAUT + 0.339134*LONG + 0.001872*AR - 0.975766*PSIL - 2.055881*SIDWIN - 0.365898*DP01SUM - 0.017957*PWIN + 0.02393*PSPR + 0.65228*LAT + 2.924699*SIS + 0.810424*TXWIN + 0.084916*DTN0AUT + 0.223632*DP01SPR

Carcharodus palaemon Y=14.618217 + 0.00485*AR - 0.026782*PSUM - 2.166948*DTN20 - 13.426487*SIS + 0.002007*ALT + 1.885136*DTN20AUT + 1.660546*CALC + 0.061191*DHI + 1.695364*CTI

Carcharodus tripolinus Y=3.159746 - 1.966748*DP10SUM + 1.061402*CALC + 0.000509*HPD + 0.757068*TJAN - 0.940916*TSPR

Celastrina argiolus Y=49.753571 + 0.003776*AET + 0.002174*AR + 0.001156*HPD + 2.440821*SISWIN - 0.825355*TXSPR + 2.64205*SIDSPR + 0.003065*U100 - 0.165695*DP10WIN + 0.199541*DP01WIN + 0.555195*DP30AUT - 0.190957*DP01AUT - 13.904996*SISAUT + 6.173664*SIDAUT + 0.388463*CLAY - 0.372492*LAT + 3.025592*TSUM + 0.12751*DTN0AUT - 7.020281*SISSUM + 5.43728*SIDSUM - 0.018433*DTX25 - 0.522763*GYP - 1.225387*TNJUL + 0.149041*DTN20AUT - 0.024586*DTN20 - 0.872843*TXJUL - 0.002627*PWIN + 0.039762*DTX25SPR - 0.056612*DTN0SPR

Charaxes jasius Y=4.631081 + 0.003083*AET + 0.779149*LONG + 0.240416*DP30WIN + 0.380871*SIL + 0.000803*HPD - 0.005843*ALT + 0.001659*AR - 0.262339*DP1SUM - 18.202278*SIDWIN - 16.842982*SIDAUT + 51.488629*SID + 30.189511*SISWIN - 48.851465*SIS - 0.023909*PAUT + 0.003882*U500 - 22.224951*SIDSPR + 25.564182*SISSPR + 0.160116*SLOP + 0.00851*PANN + 0.057894*DP01WIN + 0.035491*DTN0WIN - 0.31436*TXANN

Chazara briseis Y=2.081935 - 0.012271*U500 - 0.017645*U100 + 0.780586*CALC + 0.00065*HPD - 1.076261*DTN0SUM - 0.211044*DTX25SPR + 1.725048*TXANN + 0.001466*AR - 18.578317*SISWIN + 0.22743*LONG + 11.726698*SIDWIN + 18.122066*SISAUT - 0.117796*DTN0WIN + 0.052198*DTX25SUM - 2.914354*SISSPR - 0.340982*DP01AUT - 2.498957*TAUT + 1.65039*TNSUM - 1.203294*TJUL - 0.307905*DTN0SPR + 0.171458*DTN0 + 0.581152*DP30SUM - 0.450913*DP01SUM + 0.160103*DP01 - 0.437391*DP10AUT + 0.010526*PAUT - 0.005152*PET - 8.43979*SIDAUT

Chazara prieuri Y=3.389266 + 0.004897*ALT + 0.001009*HPD - 2.379212*PSIL - 0.010117*U500 + 1.367239*DTN0SUM - 0.029383*PAUT + 0.364791*LONG - 2.386964*DP30SPR - 0.107031*DP1 + 0.040543*PSPR - 1.976938*TAUT + 1.960586*TSPR

Coenonympha arcania Y= - 35.167206 - 0.269067*TXJAN + 0.008314*AET - 0.00816*U500 + 0.927905*LAT + 0.00234*AR + 0.266696*LONG - 3.322497*SISSPR - 0.019814*DHI - 0.011365*PSUM + 0.39863*CALC - 0.1773*DTX25SPR + 0.038986*DTX25SUM + 0.020517*PSPR + 1.489011*SISSUM - 0.524288*DP1SPR + 0.401732*DP01SPR - 0.472712*DP01AUT + 0.343463*DP1AUT - 0.00988*PWIN + 0.112508*DP01WIN - 0.050332*DTN20 + 0.412903*DP30SPR

Coenonympha glycerion Y= - 67.104625 - 2.80418*TXANN - 0.01204*U500 + 0.979248*LAT + 0.005227*ALT + 0.00719*AET + 0.272731*DP1SUM - 0.007441*PET + 0.60979*DP30SUM + 1.081513*TSUM - 0.180363*SLOP + 0.001393*AR - 0.02214*DHI + 0.29649*DP01SPR + 13.300752*SISSUM - 11.556852*SIDSUM - 0.33823*LONG - 22.965218*SISWIN - 0.099943*DP1 + 2.414434*TXAUT + 0.062153*DTX25SUM - 0.03793*DTX25 - 1.413549*TNAUT - 0.347851*DP01SUM + 0.94273*TNSPR + 20.596353*SIDWIN - 0.044476*DTN0WIN - 0.006854*PWIN + 0.012775*PAUT

Coenonympha pamphilus Y=14.089395 - 15.266418*SISWIN + 0.002717*AET + 6.410034*SIDAUT + 0.001883*AR + 5.3533*SISSPR - 0.02275*PWIN - 0.009077*PAUT - 0.017061*DHI - 0.653189*GYP + 0.201667*SIL + 0.000401*HPD + 0.429701*CLAY + 0.16024*DTN0AUT + 1.409907*TXAUT + 0.611752*DP30AUT - 0.267083*DP01AUT - 0.12753*DTN0SPR + 0.011797*PANN - 5.825327*SIDSPR - 0.251055*DP10SPR - 0.080736*DTX25AUT + 9.090515*SIDWIN + 0.02798*DTX25SUM - 8.467065*SISSUM + 6.392133*SIDSUM + 0.094874*DTN20AUT + 0.002665*PET - 2.79523*TXANN + 1.759304*TXJAN + 0.303369*DP01SPR + 0.484871*DP1WIN + 0.399618*DP1AUT + 0.027212*DTN0 - 0.32029*DP1 + 0.289699*DP1SUM - 2.579633*TXWIN - 1.138677*TNJUL - 0.614492*TNJAN + 9.217453*TANN - 2.585602*TSPR - 2.340447*TAUT

Coenonynpha dorus Y= - 66.203179 - 0.568293*CTI + 0.000776*HPD + 0.573687*GYP - 0.084188*DTX25SPR + 0.027812*DTX25SUM + 0.001634*AR + 1.037349*LAT + 0.488169*CALC - 0.15335*DP30WIN - 0.004627*U100 + 0.762236*PGRAV - 0.021215*DTN20 + 0.869253*DP30SUM - 0.030028*PSUM +

7.29077*SID - 3.364522*SISSPR - 10.046118*SIDSUM - 0.003579*U500 + 10.985021*SISSUM - 0.27715*DP10AUT + 0.148675*DTN0AUT + 0.281117*DP10SUM - 0.029085*DTN0 - 0.409146*PCLAY + 0.011589*PSPR + 0.007691*PAUT + 0.683161*TNSPR - 0.573726*TNAUT

Colias alfacariensis Y= - 57.035651 - 0.020556*U100 - 0.093954*DTX25SPR - 0.006324*U500 + 0.354685*CALC + 0.00106*AR + 0.562768*LAT + 0.045967*DTN0WIN + 0.870435*DP01SPR - 0.746446*PSIL + 0.00033*HPD + 0.018242*PSPR + 13.454889*SISSUM - 9.961327*SIDSUM - 0.023518*PWIN + 0.104395*SLOP - 0.350431*DP1SPR - 0.397266*DP01 + 0.006725*PANN + 0.514738*DP01WIN + 0.33851*DP1AUT - 2.118918*TNAUT + 0.004245*PET + 11.126576*SIDAUT + 0.046205*DTX25SUM - 0.031556*DTX25 - 0.072945*DP10 - 14.121237*SISAUT - 0.172298*DTN0SPR + 0.226047*DTN0AUT + 2.387258*TNJAN - 2.522545*TNWIN - 1.011943*TXJAN + 0.001902*ALT + 0.939323*TNSUM + 0.246024*DP10SUM - 2.049349*TJUL - 2.566751*TSPR + 7.347695*TANN

Colias crocea Y=11.00613 - 7.615772*SISWIN + 0.001771*AR + 0.001382*HPD - 3.021706*TNSUM - 0.01772*DHI + 0.291269*CALC + 0.001508*AET - 0.18518*DP10WIN + 0.008644*PSPR + 0.205871*DTN0AUT + 0.262677*SIL + 0.012699*SE - 0.018933*PWIN + 0.903118*DP30WIN - 0.424849*DP30 + 0.407125*DP30AUT - 0.109163*DTN0SPR + 5.546387*TSUM - 1.6122*TJUL - 2.149124*TXSUM + 0.308518*CLAY + 8.049642*SID - 3.341114*SISSUM + 1.519808*TNSPR + 0.951609*PGYP + 0.002445*PET + 1.327378*TJAN - 1.5115*TNWIN - 1.475263*TXWIN + 0.172145*DTN20AUT - 0.034009*DTN20 + 6.150686*TANN - 1.276015*TAUT - 3.371718*TSPR + 0.26949*DP1WIN + 0.007258*PANN + 0.497749*DP01SPR - 0.386925*DP1SPR - 0.130646*DP01 + 0.178059*DP10SUM

Colias phicomone Y= - 148.097566 + 0.001664*AR - 0.029077*PET + 3.674034*LAT + 0.103053*DTN0SPR + 0.200395*DTN20 - 0.021727*PWIN + 0.028475*PAUT

Colotis evagore Y= - 24.430237 + 0.728151*LONG + 5.417038*SISAUT + 0.000677*HPD + 0.195919*SLOP + 0.087207*DTX25AUT - 0.049949*DHI - 0.078086*DTN20 - 0.924823*DP10SUM + 0.203599*DTN20AUT

Cupido alcetas Y= - 155.657215 + 1.442967*LONG + 1.520139*DP10SUM + 1.985816*SIL - 0.177582*DTN20 - 0.883729*DP1SUM + 2.543835*LAT + 15.49715*SISWIN + 0.512326*DP1WIN + 0.024007*U500 - 0.06618*PSPR + 0.017364*PANN - 6.322009*SIDAUT + 1.15068*GYP + 0.356322*SLOP + 1.198038*CTI - 0.688148*DP10AUT

Cupido argiades Y= - 37.187498 + 1.640489*LAT + 0.202022*LONG - 4.992613*SISSPR - 0.20298*DP1AUT - 0.017539*U100 + 0.001906*AR + 0.197732*DP01WIN + 5.783315*SIDSPR - 0.009419*PET + 0.000471*HPD - 0.196983*DP01SUM + 0.987487*DP10SUM - 0.191577*DP10 - 9.08368*SISAUT + 0.00888*PSPR + 3.558041*SIDWIN + 6.539548*TSUM - 1.197557*TSPR - 5.2331*TJUL

Cupido lorquini Y=74.342514 - 1.398228*LAT + 0.32219*DP01SPR - 1.626982*CTI - 0.062853*PSUM - 0.174223*DTN20AUT - 0.04507*DHI

Cupido minimus Y= - 0.023175 - 1.044087*TXAUT - 0.006121*U500 + 0.428877*CALC + 0.001577*AR - 0.114542*DTX25SPR - 0.532702*DP30 + 0.003613*AET + 1.307611*DP30SPR - 7.699216*SIDAUT - 8.288767*SIS + 3.212119*TXSUM - 2.319667*TXJUL + 15.016508*SID + 0.947955*DP30AUT - 0.335197*DP10AUT

+ 0.052699*DTN0WIN + 2.553706*TJAN - 2.317698*TWIN - 0.445883*DP1SUM + 0.6141*DP10SUM - 0.728955*PSIL + 0.059469*DTX25SUM + 0.177558*DP01SPR + 10.132042*SISSPR - 9.487964*SIDSPR + 0.159749*SLOP - 0.030041*DTX25 - 4.35381*TNJUL + 4.579729*TNSUM - 2.639656*SISSUM

Cupido osiris Y=13.187845 + 0.351692*LONG + 0.006011*AET + 0.540469*CALC - 0.428587*DP30WIN - 4.438781*SISSPR - 0.326548*DP1SUM + 0.448128*DP10SUM + 0.001156*AR - 0.029875*DHI - 1.325545*TXWIN - 0.211715*DTX25SPR - 0.717987*PSIL - 0.452908*CTI + 1.593827*TXSPR - 2.223286*TNJUL + 2.587617*TNSUM + 4.099199*SIS + 0.21399*DP01SPR - 0.15179*DP01AUT + 1.577016*TJAN + 0.033025*DTX25SUM - 2.398272*TANN

Danaus chrysippus Y= - 7.86113 + 0.754433*LONG - 0.003205*ALT + 4.415779*SISAUT - 0.04001*DTX25SUM + 0.000567*HPD - 0.717045*TXAUT + 1.385609*TSPR - 0.603308*TNJUL

Danaus plexippus Y= - 12.0367 + 0.000454*HPD + 0.221447*SLOP + 3.552667*TJAN - 3.98388*TSPR + 3.334661*SISSUM + 0.544429*DP10AUT - 0.198379*DP1SPR - 0.011542*PET + 0.64085*TXJUL - 0.017247*U100 - 0.005207*ALT - 0.002939*AET

Erebia arvernensis Y= - 410.879215 - 0.079551*WE + 9.296779*LAT + 1.562616*DTN0SUM + 1.070598*LONG + 0.002523*AR - 0.712263*TSPR + 60.534879*SISWIN - 30.010641*SIDWIN + 0.232771*DTX25AUT - 27.200106*SISAUT + 2.493617*SISSUM + 0.478046*DP1SPR - 0.722782*DP1SUM

Erebia epiphron Y= - 117.686786 - 0.79801*TXANN + 3.041745*LAT + 0.002634*AR - 0.204655*DP1SUM - 0.008151*U500 - 0.018486*PET - 2.492842*DP30WIN + 1.530399*DP30 - 1.743132*DP30AUT + 1.816796*SISSUM + 0.954739*TNSUM + 0.045568*DTN0 - 7.682422*SIDAUT

Erebia epistygne Y= - 23.528426 + 1.568982*CALC - 0.310886*DP01WIN + 0.325086*DP1WIN + 0.294816*LONG - 1.894688*TNSPR - 0.157493*SLOP - 0.030658*DHI - 47.687037*SIDAUT + 38.097638*SIDWIN + 4.187702*SISSUM - 0.011189*PWIN + 0.016572*U100 - 1.675819*DTN0SUM - 37.95049*SISWIN + 35.905148*SISAUT + 0.091918*DTX25AUT - 7.144804*TJUL + 0.94933*GYP + 3.497478*TXSUM + 4.785387*TNJUL + 0.004016*ALT

Erebia euryale Y= - 71.8518 + 0.002778*AR + 2.066812*LAT + 0.151086*DTN20 - 15.345107*SISSPR - 0.102084*DP1 + 9.051826*SIDSPR + 0.026248*U100 + 0.200884*DP01SPR + 0.842557*DTN0SUM + 1.296959*CTI + 0.005283*ALT

Erebia gorge Y= - 124.133144 - 0.367762*DTX25AUT + 0.343023*SLOP + 0.014997*PSUM + 2.871542*LAT + 0.052245*DTX25 - 0.233462*DP10SPR - 0.719488*TNSPR - 0.159757*DTN0AUT

Erebia gorgone Y= - 14.004017 - 0.379521*DTX25SPR + 0.063089*U100 + 0.980386*LONG + 0.02489*PAUT

Erebia hispánica Y=44.556044 - 1.789152*DP1SUM - 2.147501*TXAUT

Erebia lefebvrei Y= - 190.764189 + 0.001418*AR + 4.919177*LAT - 0.22236*DP1 - 2.555614*CTI + 1.094887*DP01SPR + 0.168326*DTN0SPR + 1.281792*DP10AUT - 0.199119*DP01 - 0.641486*DP10SPR + 8.11848*TNSUM - 6.744761*TNJUL + 0.006073*ALT

Erebia manto Y= - 1110.637139 + 0.003748*AR + 2.327299*LONG - 2.18091*DP1SUM + 4.71172*DP01SPR + 0.32683*DHI - 0.827776*DP01 + 0.265901*U100 - 1.961835*DP1WIN - 0.843161*DTN0SPR - 0.580898*DTX25AUT + 26.309966*LAT - 12.676252*TNAUT + 5.24134*TNJAN + 5.567664*DP30SPR + 0.07308*PAUT + 4.551638*TNSUM - 0.843161*DTN0SPR - 0.580898*DTX25AUT + 26.309966*LAT - 12.676252*TNAUT + 5.24134*TNJAN + 5.567664*DP30SPR + 0.07308*PAUT + 4.551638*TNSUM

Erebia meolans Y= - 6.396933 - 2.564906*TXANN + 0.001521*AR + 0.010002*AET + 0.415331*LONG - 10.336429*SISSPR + 3.647706*TXSUM - 0.034786*DHI - 0.014182*PET + 9.861374*SIDSPR + 0.042249*DTN20 + 1.45793*TXAUT - 36.46948*SISAUT + 0.004216*ALT - 0.027561*DTX25 + 0.120599*DP01SPR + 0.246143*DP10SPR - 0.156256*DP1SUM + 23.313028*SIDAUT + 0.035685*PSPR + 36.092432*SISWIN - 25.25699*SIDWIN - 2.331164*TXJUL + 0.861973*PCALC + 1.090896*LAT - 0.20541*DP1SPR - 0.010215*PANN - 0.321852*TXJAN + 0.036866*DTX25SUM + 0.820882*CALC

Erebia neoridas Y= - 99.268221 + 0.701583*LONG - 0.207277*DTX25SPR + 2.182097*LAT - 0.023437*PET + 4.597395*SISWIN + 0.277098*SLOP + 0.01784*AET - 0.05906*DHI - 0.321852*TXJAN + 0.036866*DTX25SUM + 0.820882*CALC

Erebia oeme Y= - 33.305342 + 0.00306*AR - 1.259165*DTN20 + 0.091882*U100 + 0.643204*DP01SUM - 2.349576*CTI + 5.511371*PGRAV

Erebia palarica Y= - 90.199632 - 0.028199*PET - 1.027805*LONG - 0.617483*TNJUL + 3.525348*LAT + 0.042591*DHI - 0.231172*DP01SUM - 1.721767*DTN20 - 0.013404*U500 - 2.349576*CTI + 5.511371*PGRAV

Erebia pandrose Y= - 321.549992 + 1.008619*DP10SUM - 0.686064*TXSPR - 0.556666*DP1SUM + 1.49981*LONG + 7.751211*LAT + 0.001449*AR

Erebia prono Y=91.49214 + 15.379222*TSUM + 0.003919*AR - 37.672607*SISAUT + 0.051337*U100 - 14.962202*TJUL - 26.919232*TNWIN - 0.408668*DTN0WIN + 16.673137*TNJAN + 6.125929*TNSPR + 10.029066*SIDSPR

Erebia rondoui Y= - 433.422943 + 0.051054*DTN0 + 0.538823*SLOP + 2.551363*LONG + 0.69415*DP01AUT + 4.530547*DP30AUT + 0.026432*AET + 9.353967*LAT - 1.12232*DP30 - 0.746608*DP1AUT

Erebia triaria Y= 43.712835 - 0.790639*CTI - 0.013534*PET + 0.044446*DTX25SUM + 4.422927*SIDWIN - 0.193454*DP1SUM - 0.127539*DTX25SPR + 0.00233*AR + 0.007875*U100 - 0.215773*SLOP + 3.741574*TNSUM + 0.621017*PCALC - 0.018292*DHI + 0.002883*ALT - 11.418202*SISAUT - 2.44471*TNJUL - 1.14863*TNANN

Erebia zapateri Y= - 29.914216 - 0.399364*HPD + 0.00648*ALT - 0.037691*PAUT + 0.880209*GRAV + 0.486848*DP10WIN - 11.163416*TNSPR - 0.024351*PET + 7.217286*TNANN + 4.386796*TSPR - 0.276373*DTX25SPR

Erynnis tages Y= - 23.97607 + 0.315645*DP10SUM - 1.086793*PSIL - 0.651133*CTI - 0.03228*DHI + 0.635393*LAT + 0.30602*LONG + 0.237525*DP01WIN + 0.001356*AR - 0.128473*DP01SUM + 0.000373*HPD + 0.523995*CLAY - 0.733257*PCLAY - 0.141492*DTN20AUT - 0.168298*DP01AUT + 1.090489*PGRAV + 0.009186*PET - 0.952761*DTN0AUT - 1.539146*SISSPR - 0.10313*DTX25SPR - 1.294893*DTN0SPR - 0.01148*PSUM + 0.00758*PSPR - 0.00496*PWIN +

0.710622*DP30AUT - 0.303421*DP30WIN + 1.159021*DTN0 + 0.003085*ALT - 3.086703*TNJUL + 3.376306*TNSUM + 0.265777*TXSPR - 1.13247*DTN0WIN - 1.116009*DTN0SUM

Euchloe ausonia Y= - 120.653814 + 0.001832*AR - 14.534128*SIDSUM + 2.340201*LAT - 0.546317*DP01SUM + 0.402452*DP01SPR + 14.206757*TNAUT - 15.052115*TNANN + 0.606646*DTN0AUT + 0.062004*DTX25SUM + 1.250509*LONG + 0.024609*U500 - 0.182183*DTN0WIN + 31.172626*SID - 14.820898*SIDWIN

Euchloe bazae Y= - 233.197503 + 13.865467*TXJUL - 14.117215*TXSUM + 103.285982*SISSPR - 107.19034*SID - 0.332265*PAUT - 11.471534*DP10WIN + 0.380497*PWIN

Euchloe belemia Y= - 29.336489 + 0.009065*U500 + 0.534337*DP30 - 0.877983*DP30SPR + 0.000664*HPD + 1.068783*PCALC - 0.058083*DTN0WIN - 0.017459*DHI - 1.769759*DP30SUM - 0.484137*DP10SPR - 0.472056*LONG + 0.001463*AR + 0.631557*TXSUM + 6.307539*SISSPR - 6.576535*SIDAUT + 1.503277*TXAUT - 2.16397*TXANN - 0.828082*PSIL + 0.456205*SIL + 1.251713*DTN0SUM - 0.061143*DTX25AUT + 0.023404*DTX25

Euchloe crameri Y=1.59774 + 0.386018*GYP + 0.001639*AR + 6.272755*SIDSUM + 0.215376*LAT + 0.003568*PET + 0.000694*HPD + 0.229671*DTN20AUT - 5.682767*SISSUM - 1.549855*TXWIN + 0.141141*LONG + 0.001239*AET + 2.231154*TJAN + 0.477355*DTN0SUM + 0.209859*TXSUM - 0.043492*DTN20 + 0.578019*SIL - 0.506969*PSIL + 0.009157*SE + 0.104683*DP01WIN - 0.505346*DP1SPR - 0.951476*TNJAN + 0.024823*DTX25AUT + 3.692288*SIDSPR - 6.505096*SID + 0.455194*DP01SPR - 0.137465*DP01 + 0.104838*DP1

Euchloe tagis Y= - 6.790073 - 0.008633*U500 + 9.057022*SISAUT + 0.627824*CALC - 0.01079*U100 - 0.564985*TAUT - 0.425435*DP1SUM + 0.002048*AR - 7.928759*SISWIN + 0.00034*HPD + 0.615874*TXSPR - 0.108974*DTX25SPR + 0.820631*PCALC - 2.156003*SISSUM + 0.01852*PSPR - 0.389452*DP10WIN - 0.934852*DP30SPR + 0.55257*DP10AUT + 0.28283*DTN20AUT - 0.065358*DTN20 + 0.049828*DTX25SUM

Eumedonia eumedon Y=5.533576 - 0.470531*TSPR + 1.306392*CALC + 0.001747*AR - 1.129015*DP30SUM - 0.012257*PET + 0.01306*U100 + 0.260084*DTN20AUT

Euphydryas aurinia Y=31.729158 - 0.678631*CTI + 0.004811*AET - 0.074926*DTX25SPR + 0.000596*HPD + 0.926042*TXJUL + 0.583206*SIL + 0.001674*AR - 0.271546*DP1AUT + 0.189573*DP01SPR - 0.009709*DHI - 0.166563*DP01SUM - 0.12956*SLOP + 0.041784*DTX25SUM + 7.957077*SIDAUT - 7.196133*SISAUT - 1.771265*SIDWIN + 0.343869*CLAY - 0.0656*DTX25AUT - 5.505452*SISSUM + 0.131907*DTN0AUT + 2.709442*TNSUM + 3.51122*SIDSUM + 0.192904*DP10AUT - 0.191287*DP10SPR + 0.001295*ALT + 0.286308*TXJAN - 0.122336*DTN0SPR - 0.659231*TNSPR + 0.025126*DTN0 + 0.070353*DP1WIN - 1.135629*TXSUM - 1.885382*TNJUL

Euphydryas desfontainii Y= - 37.241649 - 1.838854*TWIN + 0.2005*LONG + 0.004152*AET + 0.624027*CALC + 0.122859*DP01SPR - 0.122981*DTX25SPR + 0.006302*PET - 0.006628*U500 + 0.513081*CLAY + 0.001364*AR - 0.541595*DP30WIN - 0.364669*DP1SUM + 0.424759*DP10SUM + 0.30039*DTN20AUT - 0.05574*DTN20 + 0.287705*DTN0AUT - 0.163652*DTN0SPR + 0.977789*TNJAN + 0.652449*TXAUT + 0.005771*PWIN + 0.510409*LAT + 2.990564*SIDWIN

Favonius quercus Y= - 3.33656 + 0.777198*TXWIN + 0.005126*AET + 2.1615*TSUM + 0.566556*DP30SPR + 0.000542*HPD - 0.01356*DHI + 0.333666*SIL + 0.001781*AR + 0.366502*LAT + 0.006734*PSPR - 0.080171*DTX25SPR + 0.014245*SE + 0.026615*DTX25 - 0.404688*DP10WIN + 0.347758*DP1WIN + 0.623987*DP01SPR - 0.173207*DP01 - 0.432313*DP1SPR - 1.953079*TXANN - 0.717535*TNJUL + 0.044543*DTN0WIN + 0.241417*DP10AUT - 9.013111*SISSUM + 7.285534*SIDSUM + 0.318792*CLAY - 0.059345*DTX25AUT

Gegenes nostrodamus Y= - 23.462146 + 0.689789*GRAV + 0.000505*HPD + 0.059415*DTX25SUM + 0.014492*SE - 0.005533*ALT + 0.001878*AR - 2.406183*TAUT + 1.453324*TXJAN - 1.050822*PSIL - 0.537536*DP10WIN + 0.015559*PSPR + 0.147434*DP01WIN + 3.447128*SISSUM + 0.508799*DP10SUM + 1.906141*TNANN - 1.391593*TJAN + 0.335533*DP10AUT - 0.185649*DP01SUM

Glaucopsyche alexis Y= - 31.313174 - 0.015298*U100 - 0.268854*LONG + 0.003392*AET - 0.227797*DP10WIN + 0.001597*AR + 7.945495*SISWIN + 13.408667*SISSUM - 20.310524*SIS + 0.018776*PSPR - 0.022878*DHI - 0.009904*PWIN + 0.000514*HPD + 6.542426*TSUM - 5.012043*TJUL - 2.772346*TAUT - 0.113165*DTX25SPR + 0.347941*CALC + 0.501971*DTN0SUM + 0.143535*DTX25AUT - 0.031407*DTX25 - 0.311882*CTI - 4.881349*SIDSUM + 0.245709*DP30WIN + 0.497427*DP01SPR - 0.38043*DP1SPR + 0.504854*LAT - 2.194156*TXSPR + 2.477644*SIDSPR + 2.002333*TXANN - 0.125993*DP01SUM - 0.392019*DP01AUT + 0.288846*DP1AUT + 0.236354*GRAV + 1.808009*TSPR

Glaucopsyche melanops Y= - 36.531532 + 0.001433*AR + 1.134883*SIDSUM + 0.728322*LAT + 0.000614*HPD + 0.001408*AET - 0.020278*DHI - 0.37685*CTI + 0.850475*DTN0SUM + 0.687981*SIL - 0.642729*PSIL - 0.172835*DP30AUT + 0.007462*SE + 0.670438*TJAN - 1.585919*TXWIN + 0.27408*TXJUL + 0.976284*TXJAN + 0.062325*DTN20AUT

Gonepteryx cleopatra Y= - 4.310883 + 0.002067*AR + 0.419746*CALC + 0.002818*AET + 0.000705*HPD + 0.412752*LAT - 0.087515*DP10 + 0.014953*PSPR + 5.029436*SIDSUM - 6.46849*SISSUM + 0.237886*DTN0AUT + 0.144204*LONG + 0.309532*DP1WIN - 0.111504*DP1 - 0.007999*DHI - 1.72206*TSPR + 1.93667*TANN - 0.092134*DTN0SPR - 0.004738*U100 + 0.389088*SIL - 0.739549*PSIL - 0.281843*CTI + 0.424868*DP30AUT - 0.004858*PANN - 0.001835*ALT + 0.018705*PSUM - 0.403145*PCLAY - 1.809009*TXWIN + 1.373131*TXJAN - 1.370558*TJUL + 1.50948*TSUM + 0.107163*DP01SPR - 0.135795*DP01SUM + 3.504472*SISSPR - 2.541356*SIDSPR

Gonepteryx rhamni Y=19.866538 + 0.001603*AR - 0.003342*U500 - 0.102065*DTX25SPR - 1.941268*SISWIN - 1.472176*TJUL + 0.00065*HPD + 0.004277*AET - 0.02554*DHI - 0.696001*DP30WIN + 0.037547*PSPR - 0.011843*PANN + 11.608749*SIDAUT - 0.080976*DP10 - 0.479676*CTI + 2.040976*TSUM - 11.712788*SISAUT - 0.205007*DP1AUT - 0.168038*DTN0WIN + 0.016451*PAUT - 0.327525*DTN0SPR + 0.268336*DTN20AUT - 0.058351*DTN20 - 0.364385*TXSPR + 0.231969*DP1WIN + 0.405884*DP30 - 0.004077*U100 + 0.301231*DP01SPR - 0.180993*DP1SPR - 0.060755*DP01 + 0.20134*DTN0

Hamearis lucina Y= - 11.20197 - 0.789809*TXAUT + 0.874952*DP30SUM + 0.007434*AET + 0.305454*LONG + 0.737535*LAT - 0.253016*DP1SUM - 0.017319*PET + 0.001622*AR + 0.638695*TSUM - 0.476323*DTN20AUT - 0.015482*DHI + 0.148334*DTX25AUT - 0.18068*DTX25SPR + 0.987946*GYP - 2.294098*SISSPR + 0.421083*SIL

Hesperia comma Y= - 56.001484 - 0.007685*U500 + 0.007693*AET - 0.029156*DHI + 0.000432*HPD + 0.754023*DP30SUM - 0.122487*DTX25SPR + 0.001813*AR - 10.286232*TNANN - 0.340019*DP1SUM + 0.26623*DP01SPR + 0.830055*LAT - 3.528198*TXSPR + 6.437807*TSPR - 0.134249*DP01AUT +

5.023681*SISSUM - 3.465525*SIDSUM + 0.006185*PSPR - 0.004887*PET + 0.029279*DTX25SUM + 7.13872*TNSUM + 2.91899*TNJAN - 2.814423*TNJUL - 0.153877*LONG + 0.706645*DP30SPR - 0.466087*DP30WIN

Heteropterus morpheus Y=20.723377 + 0.952639*LONG - 0.029858*PSUM - 10.286514*SIDWIN + 1.489902*CALC + 0.04326*PSPR - 0.47637*DP10SPR - 3.712616*SIDSUM + 10.087481*TXSUM - 0.987834*DP30WIN - 8.467629*TXJUL - 1.636706*TXANN - 0.064105*DHI - 0.02545*PWIN + 0.621259*DP10AUT

Hipparchia alcyone Y= - 27.890757 + 0.004105*AET - 0.003963*U500 + 0.001799*AR - 0.153677*DTX25SPR + 0.000652*HPD + 0.03391*DTX25SUM - 0.182031*DP1SUM + 0.018241*PSPR - 0.011011*PWIN + 4.762269*SISSUM - 2.685717*SIDSUM + 0.48597*LAT + 0.115027*DP01SPR - 0.097619*DP1AUT - 3.807535*SISSPR - 0.020249*DTN20 - 0.264131*CTI + 0.394203*PCALC - 0.009275*DHI + 0.00943*SE - 0.018246*PSUM + 0.434767*DP30SUM + 0.011524*PAUT - 1.747609*TNAUT + 1.746757*TNANN + 0.34926*DP10SUM - 0.192726*DP10AUT + 2.142501*SIDSPR

Hipparchia fagi Y= - 60.154926 + 0.550634*LONG + 1.219534*LAT + 0.007851*AET - 0.018703*U500 - 0.478022*DTX25SPR + 0.055253*DTX25 + 3.63312*SIDWIN + 12.54254*TSUM - 10.528976*TJUL - 0.969581*DTN20AUT + 0.163325*DTN20 - 2.610721*TANN + 0.712835*PCALC - 0.569768*DP10AUT + 0.48518*DP1WIN + 0.001127*AR + 0.902861*DP30SPR - 0.118246*DP1 + 0.48693*DP10SUM

Hipparchia fidia Y=5.937724 + 0.001592*AR - 0.005633*U500 + 0.196841*DP1WIN - 0.056576*DTX25SPR + 0.011381*SE + 0.001876*AET + 0.000323*HPD + 0.344008*CALC - 0.010678*DHI + 0.107168*DTN0AUT - 0.41359*CTI + 0.232325*DP01AUT - 0.871308*DTN0SUM - 0.32536*DP10WIN - 0.069715*DP1 + 0.261575*DP30 - 0.042317*DP01 + 0.634632*DP30SUM - 0.009539*PSUM - 0.001411*ALT - 1.691965*TAUT + 1.691153*TANN

Hipparchia semele Y=13.295783 + 0.361303*TXAUT - 0.002993*U500 + 0.001971*AR + 0.377584*CLAY - 0.072722*DTX25SPR - 0.782071*TXJAN - 0.00954*U100 + 0.001979*AET + 0.00052*HPD + 0.234625*CALC + 1.470936*TWIN + 5.338373*TNSUM - 2.547844*TNJUL + 0.501578*DP01SPR + 0.607555*DP10SUM + 12.833056*SIDWIN - 13.440654*SISWIN - 0.113946*DP01 - 0.530216*TXSPR - 0.219313*DP1 - 0.016559*PSUM + 1.373565*TNJAN + 0.007891*PAUT - 0.18345*DP10WIN + 0.347758*DP1WIN + 0.262553*DP1AUT - 0.107129*DTN0SPR + 0.135114*DTN0AUT - 6.401189*TNANN - 0.0174*DTN20 + 1.40564*TNSPR

Hipparchia statilinus Y= - 44.765535 + 0.002695*AET + 0.000878*HPD + 0.002164*AR - 0.003441*U500 + 0.874015*LAT + 0.382496*DP01WIN + 0.640799*DP01SPR - 0.283224*DP01 - 0.247277*DP1SPR + 0.203937*DP1AUT + 0.130048*DTN0AUT - 0.083055*DTN0SPR + 0.033882*DTX25SUM + 0.337451*DP30SUM - 1.701878*TSPR + 4.784298*TANN - 0.754522*TXWIN + 0.253754*DTN20AUT - 0.046444*DTN20 - 2.029905*TNAUT - 4.636606*TXSUM + 3.019705*TXJUL - 0.053583*DTX25AUT + 5.32783*TSUM - 4.049539*TJUL - 0.145945*DP10WIN + 3.162811*SID - 1.380075*SIDSPR + 0.00512*PAUT

Hyponephele lupina Y= - 16.424836 + 0.052657*DTN0WIN + 3.607366*SIDSUM - 0.007639*U500 + 0.001641*AR - 0.064554*DTX25AUT + 0.034823*DTX25SUM + 4e - 04*HPD - 2.004974*SIS - 0.957858*TSPR - 2.373399*TNAUT + 3.848107*TNANN + 0.839454*DP30SPR - 0.373894*DP30WIN - 0.17436*DP10 + 0.669897*TXAUT - 0.271447*TXJUL - 0.068953*DTN20 + 0.245538*DTN20AUT - 0.733057*TNWIN + 0.408304*DP10AUT + 0.009665*PSPR

Hyponephele lycaon Y= - 59.882082 - 1.292782*TXWIN + 0.005019*AET + 0.106916*DTX25SUM - 0.121071*DTX25AUT - 0.029426*DHI + 0.001569*AR + 0.351067*CLAY + 0.57317*DP30AUT + 1.557317*TXAUT - 0.006921*PET + 1.856603*TNSUM - 0.007107*U100 + 20.392975*SISSUM - 41.206087*SIS + 0.005174*ALT + 33.676699*SID - 14.521262*SIDSUM - 0.029839*DTX25 + 0.720391*DP30SPR + 0.730956*DP30SUM - 13.311628*SIDWIN + 0.568317*PCALC - 1.635542*TNAUT - 1.13674*TJUL + 1.24857*TJAN + 1.041916*LAT + 15.395745*SISWIN + 0.384209*DP01SPR - 0.141212*DP10 + 0.301844*DP01WIN - 0.192633*DP01

Iolana debilitata Y= - 7.70691 + 0.586301*LONG + 2.361493*TNJAN - 0.030869*U500 - 0.20238*DP1SUM + 0.301903*DP1WIN + 0.957663*CALC + 0.00053*HPD + 0.77645*SIL - 0.263472*DP01AUT - 0.170357*DTX25SPR + 0.435232*TXAUT - 3.119524*TNWIN + 1.230988*DTN0SUM - 0.250472*DTN20AUT + 0.047716*DTX25SUM

Iphiclides podalirius Y=9.629224 - 3.726833*SISWIN + 0.001786*AR + 0.00117*HPD + 0.006588*SE - 0.006763*U100 + 0.001427*AET - 0.510122*DP10WIN + 0.498836*SIL - 0.007819*DHI + 1.2966*PGYP + 0.567087*DP01SPR - 0.127903*DP01 + 0.168262*TNWIN + 0.161805*DTN0AUT - 11.968833*SISAUT - 0.119679*DP01SUM + 0.007391*PSUM - 0.419609*PSIL - 0.279041*DP1SPR + 10.161931*SIDAUT - 0.197367*CTI + 0.184314*CLAY + 0.185531*DP10 - 0.020624*DTN0 + 0.028439*DTX25SUM + 3.832105*SIS + 0.162427*DP1WIN + 0.218308*DP30WIN - 0.373202*DP30SPR - 0.014955*DTX25

Issoria lathonia Y=16.614473 - 1.53161*TXWIN + 0.000914*AET + 0.000681*HPD + 0.001736*AR - 4.93047*SISWIN + 0.568995*TXAUT + 1.316515*TXJAN + 8.021192*SID + 0.668295*DP01SPR - 0.624774*GYP + 0.17971*DTN0AUT - 0.156379*DP01 - 0.433518*DP1SPR - 4.338613*SIS - 0.405266*TXSPR - 0.093139*DTN0SPR + 0.00352*PET - 0.243025*PSIL + 0.429143*DP30AUT - 0.008131*PWIN + 0.168469*DP1WIN + 0.010494*PSPR - 4.133967*SISAUT + 0.000904*ALT

Kanetise circe Y= - 50.205812 + 0.002275*AET + 0.001704*AR + 0.016977*SE - 1.445727*TXWIN + 1.191846*LAT - 0.684617*TXSUM + 0.066003*DTX25SUM + 0.987236*DP01SPR - 0.388271*DP01 + 0.556722*DP30SPR + 0.237154*LONG - 2.747033*SISSPR - 0.11583*DTN20 + 0.490293*DTN20AUT - 0.411732*DP1SPR + 0.27409*DP01WIN + 0.003833*ALT - 1.583171*TNAUT + 1.759315*TXAUT - 0.140889*DTX25AUT + 1.080298*TJAN - 0.144765*DTN0SPR + 0.176961*DTN0AUT + 9.759468*SID + 0.250747*DP01AUT + 1.194557*TNSUM + 0.210855*DP1WIN - 0.07553*DP10 + 0.019539*PAUT - 0.004905*PANN + 0.427893*DP10SUM - 6.492947*SIS

Laeosopis roboris Y=72.845405 + 0.002807*AET + 0.000684*HPD + 14.780087*SISSPR + 43.375529*SID - 0.474729*DP10WIN + 0.220072*SLOP - 0.020012*DHI - 0.392843*TXWIN - 34.733173*SIS - 17.824501*SIDSPR + 0.031288*DTX25SUM - 0.764211*LAT + 0.317954*CLAY + 0.427329*SIL + 0.021778*DTN0 + 5.632846*TANN - 0.557916*DP01SUM + 0.147196*DP10 - 8.130526*SISAUT + 0.000781*AR - 0.130896*DP1SPR - 3.055468*TNJUL + 3.239327*TNSUM - 3.194622*TAUT - 1.739706*TSPR - 3.390648*SIDSUM - 0.542989*DP01AUT + 0.31934*DP01 + 0.292797*DTN20AUT - 0.063023*DTN20 - 0.221662*DP01WIN - 0.053558*DTX25SPR

Lampides boeticus Y=22.825551 + 0.00157*AR + 0.001316*HPD - 2.345284*SISWIN + 6.094862*SIDAUT - 6.676973*SISAUT + 0.001644*AET - 0.505372*DP10WIN + 0.352344*SIL + 1.704023*TNSUM - 1.138393*TXSPR + 0.599952*PCLAY + 0.17419*DP01WIN - 0.138518*DP01AUT + 0.165874*DTN0AUT - 0.061173*DTN0SPR - 0.202862*DP1SUM + 1.665354*TXANN - 1.41609*TNJUL + 0.237133*DP10 + 1.437854*SISSPR + 0.008351*SE

- 4.56409*SISSUM + 3.145817*SIDSUM - 0.653351*TXAUT - 0.376003*DP10SPR - 0.273768*CTI + 0.718201*PGRAV + 0.378846*DP30WIN - 0.340841*DP30SPR + 0.011445*PSPR - 0.006499*PWIN

Lasiommata maera Y=0.978372 + 4.900298*TXANN + 0.00261*AR - 0.005078*U500 + 17.87504*SIDAUT + 0.000369*HPD + 9.491512*SISSUM - 0.005322*U100 - 8.343672*SIDSUM - 2.160892*TXSPR - 19.604126*SISAUT + 0.259034*DP01SPR - 0.579052*DP30WIN + 0.397702*DP30 + 0.0151*PSPR - 0.00849*PWIN + 0.00171*AET - 0.491976*DP30SPR + 0.027619*DTX25SUM - 2.476078*TAUT - 1.557075*TXJAN - 3.342476*TNJUL + 4.010638*TNSUM - 0.790685*TXJUL + 1.41075*TWIN - 0.017106*DTX25 - 0.259615*DP01SUM - 0.161821*DP1SPR

Lasiommata megera Y=17.631347 + 0.001712*AR + 4.984995*TSUM - 0.853136*TXSPR + 0.223771*DTN0AUT + 0.00065*HPD - 1.648425*SISSUM + 0.272867*CALC - 1.455482*TJUL - 0.015323*DHI + 0.001911*AET - 0.243136*DP10WIN - 3.825263*SISWIN + 0.002509*PET + 0.813266*PGYP + 0.008352*SE + 0.287145*SIL + 13.34886*SIDAUT + 0.337427*DP10SUM + 0.246338*DP1WIN + 0.330981*DP30AUT - 0.2466*CTI - 0.004633*U100 + 0.002899*U500 - 0.090547*DTN0SPR - 15.32462*SISAUT + 0.210027*GRAV + 0.346934*PCALC - 1.496859*TXSUM + 1.266807*TJAN + 0.350511*DP01SPR - 0.089364*DP01SUM + 5.423856*SIS - 1.876418*TNSUM - 0.597204*DTN0SUM - 0.362021*DP1SPR - 0.072532*DP01 + 0.013159*PSPR - 0.007225*PWIN + 0.5307*DP30WIN - 0.215282*DP30 - 0.839227*TXWIN - 0.684727*TNJAN + 1.076318*TANN

Lasiommata petropolitana Y= - 74.805676 + 1.290346*SLOP + 0.340399*U100 + 0.229491*DHI + 3.437356*DP30SUM

Leptidea reali Y= - 4.103299 + 0.763369*LONG - 0.014522*PET + 0.009172*AET + 2.264049*SIDWIN + 0.534396*DP1SPR - 0.135657*DP1

Leptidea sinapis Y=51.574399 + 0.002087*AR + 0.00419*AET - 0.400745*DP10WIN - 0.428766*LAT - 0.604522*TXWIN + 0.000665*HPD + 0.01379*PSPR - 0.00279*ALT - 0.493265*CTI - 0.008701*PWIN + 0.3911*DP1WIN + 0.358721*DP30AUT + 0.618516*DP01SPR + 0.203602*DTN0AUT + 0.040615*DTX25SUM - 1.180053*TXSPR + 1.165462*TXANN - 0.020191*DTX25 + 0.367752*TAUT - 0.118465*DTN0SPR - 0.002535*PET + 0.824395*PGYP - 39.023515*SISAUT + 31.317012*SIDAUT + 0.303685*CLAY - 0.0045*U100 - 0.578629*DP1SPR - 0.146181*DP01 + 22.311055*SIS - 15.237789*SID - 2.302093*SISSUM + 0.173145*DP10 - 0.250934*DP10AUT

Leptotes pirithous Y=16.891349 + 0.001772*AR + 0.001129*HPD + 0.008289*U100 + 0.001151*AET - 0.011632*DHI + 5.704601*SIDSUM - 0.739169*TXSUM - 1.100059*GYP - 1.574639*TNJAN - 25.731362*SIS - 0.102378*DP1SUM + 0.002105*ALT - 0.85881*TNJUL + 0.043235*DP01 - 0.057214*DP1 + 0.005201*PAUT + 6.750623*SISSPR + 0.784627*DP30WIN - 0.451306*DP30 + 0.149372*DP10AUT + 7.953127*SISWIN + 0.330878*CALC - 0.765604*PCALC + 2.19908*SIDSPR + 7.50852*TANN - 2.634003*TXJAN + 2.380538*TXWIN - 3.683891*TSPR + 1.683791*TNSPR - 1.688552*TAUT - 0.025278*DTX25AUT

Libythea celtis Y= - 18.448863 + 0.002179*AR + 0.16462*LONG + 0.006615*AET + 1.298249*SIDSUM + 0.000913*HPD - 0.191746*DTN20AUT - 0.069267*DP1WIN + 0.739272*SIL + 0.210721*DP30 - 0.623633*TXJUL - 0.006033*PET - 0.004049*U500 + 1.344022*TJUL - 0.001325*ALT - 3.955803*SIDAUT + 2.557364*SISSPR - 0.761955*TNANN

Limenitis camilla Y= - 34.194386 + 0.361434*LONG - 20.928587*SISSPR + 0.000585*HPD + 0.00185*AR - 0.012962*U100 - 0.271579*DTX25SPR + 1.719715*TXSPR - 1.073985*TXANN - 0.082156*DTN20 - 0.507657*CALC + 0.042411*DTX25 + 32.819813*SIS - 6.957453*SIDAUT + 10.773478*SIDSPR - 0.180771*DP1AUT + 0.008244*PSPR + 0.934866*LAT - 17.689438*SID - 0.004433*U500 - 0.771393*TXJUL

Limenitis reducta Y=14.382842 - 0.558031*TXWIN + 0.004605*AET - 0.005927*U500 + 0.002085*AR - 0.08788*DTX25SPR + 0.057141*DTX25SUM - 4.640025*SISSPR + 12.044885*SIS + 0.000501*HPD + 0.247553*LONG - 0.15658*DTN20AUT + 0.052348*DTN0AUT + 0.70551*DP01SPR + 0.009991*PSPR - 1.742824*TXJUL - 0.517677*DP01AUT - 0.004237*PET - 2.988505*TNAUT + 2.985684*TSUM + 0.999431*TNJAN - 22.508827*SISAUT + 12.423339*SIDAUT - 0.834552*PCLAY + 0.35154*CLAY - 0.132635*DP10WIN + 0.536934*DP30AUT - 0.396164*DP30WIN - 0.275204*DP01SUM - 0.472365*DP1SPR + 0.396166*DP1AUT + 1.518497*TAUT - 0.085447*DTX25AUT

Lopinga achine Y=1206.431578 - 602.847749*SISAUT + 0.012721*AR - 1.519147*DTX25AUT - 2.482141*DP01WIN + 414.134823*SIDAUT - 1.607089*DP10

Lycaena alciphron Y=4.051075 - 0.230263*TXSPR + 0.002421*AR + 0.003428*AET + 1.9742*SIDSUM - 5.308656*SIDAUT - 0.14635*DP1 + 0.579735*DP01SPR + 0.008865*PSPR - 0.010942*DHI - 0.004715*PET - 0.09858*DTX25SPR - 0.436013*DP01AUT - 0.385994*DP1SPR + 0.438203*DP1AUT - 0.111495*DTN20AUT - 0.439303*CALC + 0.282642*CLAY - 0.855825*GYP + 0.179029*LONG + 0.148163*DP1WIN + 0.024453*DTX25

Lycaena bleusei Y=145.717201 + 37.052611*SIDSUM - 41.813401*SISSUM + 1.238929*LONG - 1.214545*CTI - 0.014456*PET - 4.280167*PCALC + 0.004136*AET - 2.848438*DP30SUM + 0.023676*PAUT + 0.454949*DTN0WIN + 0.000764*HPD - 0.221479*DTN0 + 0.116477*DTX25SUM + 2.326988*TNWIN - 10.684851*TAUT + 16.139711*TANN - 8.402609*TSPR - 0.545267*DP10 + 0.627832*TXJAN + 0.490285*DP1WIN + 0.888173*CALC + 0.010159*U100 + 1.27163*DP10AUT - 0.453956*DP1AUT

Lycaena helle Y=10.031859 - 0.993756*TJUL

Lycaena hippothoe Y=52.336883 + 0.003018*AR - 0.011652*PET + 0.497336*DTN20AUT - 33.282328*SISAUT + 20.67317*SIDAUT + 0.771058*DP01SPR - 0.23804*DP01 + 0.985341*DTN0SUM + 0.783751*DP10SUM + 3.091045*SISSUM - 0.302625*DP10AUT - 0.007256*AET - 0.193292*SLOP

Lycaena tityrus Y=38.885351 - 0.006945*U500 - 0.122422*DTX25SPR + 3.34311*TXWIN - 0.129826*DP10AUT + 0.08094*DP30 - 0.099269*DTN20 + 0.446333*DTN20AUT - 2.522744*PGRAV - 3.558694*TXSUM - 2.587365*TXJAN + 0.034348*DTX25SUM + 5.120057*TJUL - 2.680398*TNJUL + 0.824504*DTN0SUM - 0.068806*DTN0SPR + 1.962863*TNSPR - 4.108633*TNWIN - 0.008847*PET + 3.478425*SIDSUM + 2.341249*TNJAN - 9.76631*SIS + 0.001308*AR

Lycaena virgaureae Y=48.39197 - 1.336544*TXSPR - 0.019937*PET + 0.002161*AR - 19.495494*SIDWIN - 32.99097*SISAUT + 7.427395*TSUM - 0.138755*DP1 + 21.831613*SIDAUT + 0.276338*DP01SPR + 28.354324*SISWIN - 0.191014*DTX25SPR + 0.050045*DTX25SUM - 2.533583*TXJUL - 0.020277*DHI - 0.737665*TNWIN - 0.658763*DP30SUM + 0.519952*DP30SPR - 2.725699*TNJUL + 5.321504*SISSUM - 13.953287*SIS

Lycaena. phlaeas Y=12.14422 + 0.002105*AET + 0.34398*SIL + 0.001035*HPD + 5.575259*SIDSUM - 0.214536*TXWIN - 5.641347*SISSUM + 0.001544*AR - 0.216439*DP10WIN + 0.375736*DTN0SUM + 0.269756*CLAY + 0.685404*TSPR - 0.017465*PWIN - 1.389246*TXSPR + 1.112274*TXANN + 0.080906*DTN0AUT - 0.002594*U100 + 0.006888*SE + 0.353483*CALC - 0.441258*PCALC + 1.111804*SIDSPR + 0.29356*DP30AUT - 0.096772*DP01 + 0.007928*PANN + 0.535268*DP30WIN - 0.213075*DP30 + 0.374247*DP01SPR - 0.355114*DP1SPR + 0.282703*DP1WIN - 3.040294*SISAUT

Maniola jurtina Y=14.309799 - 14.451781*SISWIN + 0.001969*AR + 7.415994*SIDAUT - 6.150808*SISSUM + 0.000883*HPD - 0.301015*DP10WIN + 0.00308*AET - 0.019752*DHI + 8.035704*SIDSUM + 0.012974*PSPR - 0.008136*PWIN + 0.192157*DTN0AUT + 0.19732*DP10SUM + 0.318928*DP30AUT + 12.398942*SIDWIN - 2.988278*TXWIN + 1.801788*TXJAN - 0.097069*DTN0SPR + 0.753617*TXAUT - 0.920569*TJUL + 4.883546*SISSPR + 0.739388*PCLAY + 0.087707*SLOP - 14.089997*SID + 0.194964*DTN20AUT + 0.774475*TSUM - 0.034764*DTN20 + 0.002503*PET - 0.056179*DTX25AUT + 0.329405*SIL + 0.626148*PCALC + 0.95351*PGYP + 0.012764*DTX25 + 0.005743*PAUT + 0.47045*DP01SPR - 0.388986*DP1SPR + 0.315975*DP1WIN + 0.598059*TJAN - 0.132395*DP01

Melanargia galathea Y= - 408.415938 + 9.051605*LAT + 0.337735*LONG - 3.099249*TXJAN + 0.002667*AR + 1.423007*TNSUM - 0.213469*DTX25SPR + 0.160968*DTX25SUM + 1.367968*GYP - 4.647959*TSPR + 3.678302*TXAUT + 2.45148*TJAN - 2.813092*PGRAV - 0.889293*DP30WIN + 0.020454*PSPR - 0.413918*DP1AUT + 0.300045*DP1WIN - 0.234776*DTX25AUT - 0.014399*PWIN + 0.016677*PET + 0.800775*CTI + 0.673555*DP30AUT - 9.661914*SISSPR + 0.010142*U100 + 7.034962*SIDSPR

Melanargia ines Y=3.975853 + 0.002395*AR + 0.000798*HPD - 5.576053*SISSUM + 0.620537*CALC + 3.791377*TNWIN - 10.242078*SIDAUT + 6.498261*SIDSUM + 0.251248*DP30 - 0.011911*DHI + 2.610876*TNSUM + 0.433467*SIL - 2.483128*TNJUL + 0.028351*DTX25SUM - 3.897719*TAUT + 4.043769*TJAN - 3.088194*TSPR - 0.002578*ALT + 0.076616*DP01WIN - 3.147973*TNJAN - 4.791941*TWIN + 6.913796*TANN - 0.173443*DP10 + 0.009418*SE + 0.003024*PET + 6.738265*SISAUT + 0.120961*DTN20AUT - 0.028216*DTN20 - 0.600878*DP30SUM

Melanargia lachesis Y= - 25.285624 - 0.002618*U500 + 0.003328*AET - 0.007846*U100 - 0.478145*DP1SPR + 0.000649*HPD + 0.428905*LAT + 0.476146*DP30WIN - 0.091975*DP10 + 0.00164*AR - 0.0565*DTX25SPR - 0.439286*TNJAN + 0.666286*PCLAY + 0.691383*DP01SPR - 0.221321*DP01 - 0.650221*DTN0SUM + 0.010022*SE + 0.017281*PSPR - 0.006456*PANN + 0.169548*DTN0AUT - 0.08769*DTN0SPR + 0.258994*TXJAN - 14.700456*SIDAUT + 0.115602*DP1 + 0.504041*DP30SUM + 0.001375*ALT + 0.872728*PGYP + 0.80946*TNSPR - 0.692651*TAUT - 14.514559*SISWIN + 11.570988*SIDWIN + 15.746866*SISAUT - 0.008093*WE + 0.141516*DP01AUT + 0.010847*PAUT + 3.834638*SIDSUM - 2.361039*SISSUM

Melanargia occitanica Y= - 21.718229 - 0.016478*U100 + 0.185259*LONG - 0.774888*TXWIN - 0.030503*DHI - 0.374153*CTI + 0.259976*DP01WIN + 0.450344*GRAV + 0.000571*HPD + 0.700224*LAT + 0.001549*AET + 0.325152*CALC + 0.000961*AR - 0.954759*TNAUT + 0.460323*DP1AUT - 0.467206*DP1SPR - 0.002129*U500 + 0.011029*SE + 1.39892*TJAN + 0.037631*DTX25SUM - 0.971428*PSIL + 0.629547*SIL - 0.039037*DTX25SPR + 1.023407*PGYP - 0.505545*DP10AUT + 0.817404*DP30AUT + 0.79808*DP01SPR - 0.286428*DP01 + 2.122626*SIDAUT - 0.010843*DTN0

Melanargia russiae Y= - 68.684766 + 1.54379*TXWIN + 0.008466*AET - 0.011876*PET + 0.381918*LONG - 0.031965*DHI - 0.211889*DP01SUM + 0.001931*AR + 1.58387*LAT - 7.481498*SISSPR + 0.75484*PCALC + 0.535206*DP30SPR - 0.053659*DTN0WIN + 0.004934*ALT + 0.163564*DP01SPR + 0.01036*U100 - 0.192942*DP1AUT + 12.673764*SIS - 1.438564*TXJAN + 0.057658*DTX25SUM - 0.031544*DTX25 - 13.779855*SISWIN + 10.34523*SIDWIN

Melitaea aetherie Y=57.914611 - 4.185016*LAT - 1.409893*LONG + 1.319119*CLAY + 1.491906*SIL + 0.000813*HPD - 1.346056*TXJAN + 1.505341*TSPR - 0.03909*DHI + 1.08843*DP1SUM + 10.300761*SISSUM - 11.452201*SIDWIN + 4.808332*SIDSPR + 1.190259*DP10SPR - 0.472927*DP10WIN

Melitaea athalia Y=31.90144 - 1.812105*TXSPR + 0.005367*AET + 0.00163*AR - 1.283647*TXJAN - 0.079789*DTX25SPR + 0.054315*DTN0AUT - 0.019068*DHI - 1.144083*TNJAN + 1.837778*SISSUM - 0.278518*DP1AUT + 0.214023*DP01SPR - 18.519165*SISAUT + 0.003307*ALT - 1.109478*PGRAV + 6.253449*TJUL - 2.541157*TNAUT - 4.450976*TXJUL - 3.214467*TNJUL - 0.088867*DP01SUM - 0.026059*DTX25 + 3.412611*SIDWIN + 4.557585*TXANN + 0.553734*DP30SUM + 4.109582*TNANN + 0.042064*DTX25SUM + 7.320685*SIDAUT - 0.448971*LAT

Melitaea cinxia Y=1.246481 - 2.871558*TWIN + 0.006579*AET + 1.613031*TNSUM + 0.001203*AR + 0.45237*SIL - 0.221373*DTN20AUT - 0.018435*DHI - 0.122579*DTX25SPR - 4.138538*SISSPR + 0.00426*ALT - 18.397915*SISAUT + 10.218257*SIS - 0.44015*DP1AUT + 0.541525*DP01SPR + 0.007489*PSPR - 0.364592*DP30WIN + 0.534198*DP30AUT + 7.881746*SIDAUT + 0.026482*DTX25SUM + 0.00555*U100 - 0.577057*DP10WIN + 0.333806*DP1WIN + 0.030348*DTN0WIN - 0.333829*DP01SUM - 0.434427*DP1SPR - 0.178061*DP01WIN + 0.347536*DP1SUM + 2.463198*TNWIN - 5.88033*TNANN - 0.643487*DP10SUM + 0.232552*DP10 - 1.642359*TXSUM + 6.942464*TANN

Melitaea deione Y=8.733995 - 0.511928*CTI + 0.002271*AET + 0.001703*AR + 1.218718*SIDSUM - 0.011141*PWIN + 0.000499*HPD - 0.016195*DHI + 0.006226*U100 + 5.462925*SISWIN - 0.183839*DP1AUT + 0.168155*DP01SPR - 7.757192*SISAUT - 0.737027*GYP + 0.61554*DTN0SUM + 0.042997*DTX25SUM + 0.016524*PAUT + 0.301728*SIL + 0.149953*DP30 - 0.320774*DP10AUT + 0.198203*DP10WIN - 0.106173*DP01SUM + 0.148757*DTN0AUT + 3.708789*TNSUM - 2.740812*TNJUL - 0.403926*TSPR - 0.047997*DTN20 - 0.168277*LONG - 0.589217*TNAUT - 0.026805*DTN0 - 0.05342*DTX25SPR

Melitaea diamina Y= - 30.78759 + 0.002841*AR - 0.203064*DP1SUM + 1.128314*LAT + 0.555613*LONG + 0.186581*DP01SPR + 0.013933*U500 - 0.014884*PET - 0.505621*DP1AUT + 0.157065*DP10 - 15.432277*SISAUT + 9.890853*SIS

Melitaea didyma Y= - 55.633873 + 0.27074*LONG + 0.002632*AET + 0.000694*HPD + 0.000963*AR - 0.015952*DHI + 1.886227*SIDSUM + 0.827714*LAT + 0.479955*DP30SPR + 0.115243*DTN0AUT - 0.035038*DTN20 + 0.164308*SLOP + 0.215211*DP01SPR + 1.729544*TWIN + 0.011128*SE + 0.029501*PAUT + 0.032207*DTX25SUM + 0.019726*PSUM - 0.013528*PANN + 0.021833*PSPR - 0.16181*DP10AUT - 0.274859*DP01SUM - 0.098774*DP1SPR - 0.051398*DTX25AUT - 1.27919*TJAN + 0.001412*ALT

Melitaea parthenoides Y=15.980798 - 0.716562*TXSPR + 0.010023*AET + 0.425442*LONG - 0.011205*PET + 0.001559*AR - 0.417193*DP1SUM + 3.977896*TXSUM - 3.067418*TXJUL + 0.279953*DP10SUM - 0.024223*DHI + 0.006286*U100 - 0.419332*DP01AUT - 0.10028*DTX25SPR - 12.112662*SISAUT + 0.134833*DP01 + 8.157804*SIDAUT - 1.926018*PGYP + 0.001796*ALT + 0.404688*DP30SPR - 0.454781*DP30WIN + 0.468409*DP30AUT

Melitaea phoebe Y= - 41.195014 - 0.010979*U100 + 0.001293*AR - 0.002341*U500 + 0.001246*ALT + 0.000452*HPD + 0.694687*LAT + 0.106296*LONG + 0.489394*DP01SPR + 0.001618*AET + 0.009904*SE + 0.108433*SLOP - 0.010726*DTX25 + 0.160328*TXAUT - 0.009877*WE - 1.666426*SISWIN -

0.159178*DP01 + 0.214095*DP10SUM - 0.225199*DP1SPR + 0.170902*DP01WIN + 0.022889*PSPR - 0.007353*PANN + 0.008554*PAUT + 1.714552*SISSUM - 0.400008*DTN0SUM - 0.434404*PCALC + 0.253793*CALC

Melitaea trivia Y=40.531396 + 0.500778*TXWIN + 0.006635*AET - 1.190557*PCALC - 0.008894*PET + 0.070893*DTX25SUM - 0.137106*DTX25SPR - 0.393591*CTI - 11.926029*SISAUT + 6.585952*SIDSUM + 6.215503*SISWIN + 0.6618*CLAY + 0.001039*AR - 1.564715*TAUT - 0.271447*DP10SUM - 5.032419*SISSUM + 0.448038*DP01SPR - 0.426374*DP1SPR + 0.00778*PSPR - 0.236301*DP01AUT + 0.770549*TNSUM - 0.92044*PCLAY

Minois dryas Y=87.454248 - 41.407154*SIS - 0.014271*U500 + 13.379848*SIDSPR - 0.329665*DP1WIN + 7.10589*SISSUM - 0.631087*DTX25SPR + 1.299084*DP30SUM + 0.272071*DTX25AUT

Muschampia proto Y=44.576289 - 0.005669*U500 - 0.465886*DP10AUT - 0.3881*CTI + 0.000851*HPD + 0.737349*CALC + 0.046821*DTN0AUT - 0.019231*DHI + 0.556473*GYP - 12.604469*SIDSPR - 8.360209*SISSUM + 0.512007*LONG + 0.498928*DP01WIN - 0.211455*DP01 + 8.225248*SIDSUM - 0.110139*TXJUL + 0.198497*DTN20AUT + 0.012636*PSPR + 0.453949*GRAV + 0.386441*DP30AUT + 17.255445*SIDWIN + 12.623787*SISSPR - 18.843879*SISWIN + 0.209601*DP01SPR - 0.027742*DTN20 + 0.000786*AR - 0.565295*LAT - 0.17524*DP10WIN - 0.022106*DTX25 + 0.046824*DTX25SUM

Nymphalis antiopa Y= - 60.382565 - 0.236014*DTX25SPR + 0.028936*DTX25 - 12.933622*SISAUT + 0.000548*HPD + 8.607596*SID - 0.005946*U500 + 0.25663*SLOP + 1.798276*PGRAV + 1.013221*PSIL - 0.304644*DP1AUT + 0.149684*DP01SPR + 0.235351*DP10AUT + 0.00551*AET - 0.010003*PET + 0.371674*TNSUM - 0.015117*PAUT + 0.020157*PSPR + 1.527059*LAT + 26.641727*SISWIN - 19.26651*SIDWIN - 4.339184*SISSPR + 0.400125*GRAV - 0.018504*PSUM + 0.626892*DP30SUM + 0.057092*DTN0AUT

Nymphalis polychloros Y=15.273695 + 0.001074*AR + 0.002455*AET + 0.000745*HPD - 0.2304*DP10SUM - 0.00472*U500 + 0.357838*DP01SPR + 0.026392*DTN0WIN + 0.448879*DP30SPR + 0.298637*SIL - 0.280311*DP10WIN + 6.68651*SIDSUM - 7.181552*SISSUM + 0.819702*TNSUM + 0.127957*SLOP - 0.047098*DTX25AUT - 0.210796*DP01AUT + 0.371015*DTN20AUT - 0.074711*DTN20 - 0.658249*TNAUT + 0.200495*DP10AUT - 0.301693*DP1SPR + 0.131507*DP1WIN + 0.005498*PSPR - 0.129928*DP01SUM - 2.780378*SIDAUT

Ochlodes venata Y=18.434083 + 0.266213*LONG + 0.0082*AET + 1.703773*SIDSUM + 0.000565*HPD + 0.0017*AR + 0.251761*DP01SPR + 0.520432*DP30SPR - 0.435835*DP30WIN + 3.03182*TSUM - 0.005256*PET + 0.575899*DP10SUM - 0.020081*PSUM + 0.005774*PSPR - 0.182439*DP10WIN + 0.307775*DP1WIN - 0.151253*DP1 - 0.121032*DP01AUT + 0.704623*DP30AUT - 0.057462*DTN20 - 5.483078*SIS - 0.012895*SE - 0.888845*TXSPR - 2.097266*TJUL - 1.107783*TNAUT + 0.837635*TNSPR - 0.002265*ALT + 0.200048*DTN20AUT

Papilio machaon Y= - 26.141708 + 0.001308*HPD + 0.001719*AR - 6.082659*SIDWIN - 1.956992*SIDSPR + 0.072968*DTN20AUT + 0.498631*CALC + 0.342051*SIL + 0.006696*SE + 0.001873*AET - 0.012989*PWIN - 0.011043*DHI + 8.350369*SID + 0.308917*TJAN + 0.556753*DTN0SUM + 0.534765*LAT + 0.019938*PSPR - 0.003631*U100 + 0.367962*DP10SUM + 0.066834*DTN0AUT + 0.258952*DP01SPR - 0.141442*DP01AUT - 0.813825*DP30SPR + 0.437496*DP30WIN - 0.133389*DP10 - 1.570668*SISSUM - 0.128058*CTI + 0.30981*DP10AUT + 0.002911*PET - 0.023546*DTX25SPR + 0.068117*DP1WIN - 0.191713*DP1SPR - 0.185683*DP01SUM + 0.268408*DP30AUT

Pararge aegeria Y=12.834127 + 0.016742*PSPR + 0.001205*HPD + 0.002508*AET - 0.020577*DHI + 0.001962*AR - 2.962226*SISWIN + 3.052225*TSUM - 0.012062*PWIN + 5.379079*SIDSUM - 5.892145*SISSUM + 0.657317*DP30WIN + 0.017818*DTN0WIN + 0.018813*DTN20 + 0.130935*DP01WIN - 0.35376*DP1SPR + 0.247842*CLAY + 0.005177*PAUT - 1.824145*TSPR - 1.913907*TAUT + 4.36499*TANN - 0.36226*DP01AUT + 0.256998*DP01SPR + 0.307321*DP1AUT + 0.002954*PET - 0.035174*DTX25SPR - 1.66131*TNJUL - 1.662314*TXSUM - 0.201335*DP30 - 0.125301*DP10WIN

Parnassius apollo Y=18.723997 + 0.594633*LONG + 0.00282*AR + 13.707323*SIDWIN - 0.300079*DP1SUM - 0.19235*DTX25SPR - 0.744648*DP30WIN + 0.757512*DP30SPR + 0.188499*DP01SPR - 0.023149*DHI + 0.006236*ALT - 17.307264*SISWIN - 0.031468*WE - 0.943261*PCLAY - 0.141524*DP01AUT - 6.03349*TJUL + 3.324777*TXSUM + 2.745956*TNJUL

Parnsassius mnemosyne Y= - 3151.61789 + 0.016032*AR + 0.903008*DTN0AUT + 4.824807*DP01SPR + 9.692393*LONG - 2.707953*DTX25SPR + 70.570057*LAT - 4.965823*DP1SUM - 4.348843*DP01AUT + 0.120962*PSUM + 0.091136*AET + 6.788349*GRAV + 0.314881*DTX25 + 0.158134*PAUT - 2.905732*DP10SPR + 2.536277*TNJUL + 0.016769*ALT

Phengaris alcon Y=15.762096 + 0.805818*DP10SUM + 0.002253*AR - 0.353745*SLOP + 0.010957*AET - 0.01845*PET + 2.773588*TNJUL - 7.914079*TXSPR + 19.439379*TXANN - 1.486558*SISSUM - 0.163203*DP1SPR - 9.977653*TXJAN + 7.228258*TXWIN - 5.443497*TXAUT + 1.578548*TNSPR - 7.534169*TSUM - 0.025792*PSPR + 0.017749*PAUT + 0.644447*DP10SPR - 0.53474*DP10AUT + 0.739154*SIL

Phengaris arion Y=37.692258 + 0.344451*DP10SUM - 2.567661*TNANN + 0.504503*CALC - 6.232823*SISSPR + 0.636501*LONG - 0.169847*DP1SUM - 0.004591*PSPR + 0.500086*DTN0AUT - 0.133483*DTN0 + 0.001071*AR - 0.082438*DTN20 + 2.031915*SISSUM - 0.00829*PET + 0.858487*TXSPR + 2.672972*TNAUT + 0.010993*U100 - 1.492496*TAUT - 0.538237*CTI

Phengaris nausithous Y=73.975916 - 0.059108*PET + 0.07639*DHI - 1.54515*DP10SUM - 1.113831*TNJAN + 0.027794*PSPR - 0.497338*DP1SPR - 15.647621*SISAUT + 1.011973*TJUL

Pieris brassicae Y= - 4.067592 + 0.006414*PAUT + 0.001496*HPD + 0.002176*AET + 0.002099*AR - 0.016931*DHI + 0.228689*LAT + 7.610285*SIDSUM - 7.930839*SISSUM - 1.762553*TNJUL - 2.918159*TXJAN + 0.561276*DTN0SUM + 1.099026*PGYP + 0.320745*SIL + 0.25507*CALC + 3.728319*TXWIN - 1.338637*TXSPR + 4.207111*TSUM - 0.014186*DTX25 + 0.007187*SE - 1.067858*TAUT + 0.85448*TSPR + 0.284425*DP30WIN + 0.229655*DP10SPR + 0.150406*DTN20AUT + 0.215119*LONG + 0.141791*DP01WIN - 0.050044*DP1 + 0.011107*PSUM - 0.243065*DP30 - 1.430429*TXJUL + 1.093055*SISSPR + 0.001025*ALT - 0.003819*PWIN - 0.030167*DTN20 - 0.015287*DTN0WIN + 0.02738*DTX25SPR - 0.21979*GRAV + 0.53126*PGRAV

Pieris ergane Y= - 107.830428 + 0.046737*DTN0 + 1.386306*CALC + 0.007699*AET + 5.52793*SISAUT + 0.565945*DP10AUT + 1.790091*LAT - 2.05262*TNSPR + 1.659616*TNAUT - 1.687837*DP30SUM + 0.625401*LONG - 0.345476*DP1SUM

Pieris manni Y= - 0.860334 + 0.543289*LONG + 0.001698*AR - 0.006271*U500 - 0.25536*DP1SUM + 1.537876*DTN0SUM - 0.071036*DTX25SPR + 0.121666*DP1AUT + 0.000381*HPD - 2.901238*TNWIN + 1.214614*TJAN - 0.147387*DTN0SPR + 0.087708*DTN0WIN - 0.367055*TXSPR + 2.060012*TNJAN

Pieris napi Y=25.9759 + 0.001156*AR - 0.004246*U500 + 0.000677*HPD + 22.551057*SIDAUT + 0.368519*CALC - 0.021575*DHI + 0.003535*AET - 0.165457*LONG - 26.724009*SISAUT - 0.386655*DP30WIN + 0.619224*DP30SPR + 0.351319*DTN0AUT - 0.206089*DTN0SPR - 1.466092*TXSPR - 0.009278*U100 - 1.144722*TNAUT - 1.614255*TXJUL + 0.70297*DP30AUT + 0.123209*SLOP - 2.26994*TNJUL + 0.024173*PSPR - 0.00645*PANN - 0.153943*DP01WIN - 0.18318*DP1AUT + 0.327321*DP1WIN - 0.214639*DP10SPR + 3.816273*SISSPR + 0.231827*DP01SPR - 0.191609*DP1SPR - 0.064371*DTN20 + 0.249118*DTN20AUT - 3.351185*SID - 0.12741*DP1SUM + 3.666248*TNSUM + 2.304146*TXSUM - 2.166997*TJAN + 3.63984*TNJAN - 5.49222*TNWIN + 5.78843*TWIN - 1.331066*TXWIN

Pieris rapae Y=48.608018 - 11.678339*SISWIN - 0.00267*U500 + 0.256671*SIL + 7.91083*SIDAUT + 0.002201*AR + 1.780914*TNSPR + 0.001377*HPD - 8.656665*SISAUT - 0.792825*TXSPR + 0.573058*DP30WIN - 0.014604*DHI + 0.000856*AET + 4.625453*TSUM + 0.256754*CALC - 1.422399*TNJUL - 1.33334*TXSUM + 0.003984*U100 - 0.64871*LAT + 0.010216*SE + 0.421674*DP01SPR - 0.335125*DP1SPR - 0.750679*TXJUL + 3.410832*SISSPR - 0.015012*PWIN - 0.317388*DP30 - 0.007851*WE + 0.161549*DTN20AUT - 0.031497*DTN20 + 5.853883*SIDWIN - 3.630587*TNANN - 0.927178*TXJAN + 0.987537*TAUT + 1.534379*TWIN + 0.006678*PANN - 3.827728*SISSUM + 3.063657*SIDSUM - 0.252877*DP10WIN + 0.159119*DP1WIN - 0.085498*DP01 + 0.609995*PGYP + 0.122094*DP10

Plebejus argus Y= - 1.110513 - 0.005314*U500 + 0.002355*AET - 0.177689*DP1SUM + 0.001774*AR - 0.023115*DTN20 + 0.000384*HPD + 1.199938*PGRAV + 0.121732*DP01WIN + 8.97696*SISSUM + 0.688998*DP30SUM + 0.416395*TXAUT - 0.182755*DP1AUT - 6.141916*SIDSUM - 0.066904*DTN0SPR + 0.100717*DTN0AUT + 0.014218*PSPR - 0.006687*PWIN - 14.160431*SISAUT + 8.951331*SIDAUT - 0.522124*DTN0SUM + 0.33238*PCALC + 1.851301*TNSPR - 0.885991*TNWIN - 2.386612*TNJUL + 2.277212*TNSUM - 1.575794*TSPR

Plebejus hespericus Y=196.89232 + 40.015738*SID - 0.019928*U500 - 0.311434*DP01SPR + 0.269064*DTN0 + 0.901994*GRAV - 2.479523*LAT - 0.64933*DTN0SPR - 48.993046*SIS - 2.577411*DP30SPR - 2.916581*PSIL + 0.004549*ALT + 1.419422*DTN0SUM

Plebejus idas Y=18.637249 + 0.005404*AET - 0.045749*DHI + 0.001462*AR - 0.268263*DP01SUM + 0.240362*DP01WIN + 0.478036*DP10SUM - 0.460522*DP30WIN + 0.654309*DP10SPR + 0.003702*ALT - 0.065813*DP1 - 2.546822*TNJUL + 1.247011*PGYP + 2.802568*TNSUM - 35.037547*SISSPR + 0.04928*DTX25SUM - 0.136536*DTX25SPR - 0.332039*DTN20AUT + 73.646067*SIDAUT - 77.37744*SISAUT + 88.054828*SIS + 30.860854*SIDSPR - 0.195196*DP10 + 0.010082*U100 - 81.260342*SID + 0.000483*HPD + 0.072724*DTN0AUT

Polygonia c-album Y=12.621547 + 0.003527*AET + 0.000743*HPD + 0.001915*AR - 0.012453*U100 + 1.689439*TSUM - 0.00557*PET - 8.266259*SIDWIN - 0.646088*GYP + 0.130798*DTN0AUT - 0.07749*DTN0SPR - 0.409136*TXSPR + 8.610936*SIDAUT - 0.082981*DTX25AUT - 11.464129*SISAUT + 0.029274*DTX25SUM - 0.713659*TXJUL - 1.070355*TNAUT + 7.552345*SISWIN + 0.528007*DP01SPR - 0.131447*DP01 - 0.235628*DP1SPR + 0.110262*DP1WIN + 0.005236*PSPR - 0.618041*DP30WIN + 0.224184*DP30 + 0.285978*GRAV + 3.130769*SIDSUM - 2.964838*SISSUM + 0.632081*TAUT

Polyommatus amandus Y=31.286379 + 0.588037*LONG + 0.0027*AET + 0.002205*AR - 0.147049*DTX25SPR - 0.391261*TXWIN - 0.01798*DHI - 4.175351*SISSPR - 0.533792*DP1SUM + 0.011082*U100 - 0.582991*DP10AUT + 0.18278*DP01SPR + 0.58802*DP10SUM + 0.009825*PAUT - 13.654161*SISAUT + 16.70169*SIDAUT + 0.032852*DTX25SUM

Polyommatus bellargus Y= - 23.442164 - 0.575537*TXWIN - 0.00305*U500 + 0.001342*AR + 0.64601*CALC + 0.158882*DTN0AUT + 0.000687*HPD - 0.314029*DP01SUM + 0.010033*PSUM - 0.143729*DTN0SPR + 0.002412*AET + 0.112054*DP01WIN - 0.280483*DP10WIN + 0.526019*LAT - 0.133089*DP1AUT + 0.277555*DP10SPR + 0.00895*SE + 0.63413*PGYP + 0.289845*GRAV + 0.094988*SLOP + 0.227063*DP30AUT + 9.278269*SIDAUT - 1.439293*TNJUL + 0.033969*DTN0 - 6.992546*SISAUT - 3.806743*SID + 1.473015*TNSUM + 0.259942*TXAUT + 2.042571*SISSPR + 2.06865*TJAN + 0.093351*DP01SPR + 0.040703*DTX25SUM - 0.01983*DTX25 - 1.59313*TWIN

Polyommatus coridon Y= - 36.610814 + 0.999381*CALC + 0.556791*DP10SUM + 0.004593*AET - 0.555886*DP30WIN + 0.383454*LONG - 0.431624*DP1SUM + 0.269373*DTN0AUT - 0.224025*DTX25SPR + 0.001143*AR + 0.206412*DP01SPR - 0.24277*DP01AUT + 2.115895*PGRAV + 0.070586*DTX25SUM - 0.042377*DTX25 + 0.865521*DP30SPR + 0.926688*LAT + 0.716516*PCALC + 0.00287*ALT - 0.063254*DTN0 - 4.200919*SISAUT + 0.133337*DP01WIN - 4.063141*TWIN + 3.387909*TJAN + 0.371053*CLAY + 0.774078*TXSPR

Polyommatus damon Y=30.876785 + 0.810349*CALC + 0.007596*AET - 0.453552*DP30 + 3.137057*TXSPR + 0.572617*DP10SPR - 0.418595*DTX25SPR - 2.978905*TXANN - 0.695536*DP1AUT - 8.322394*SISSPR + 6.788026*SIS + 1.247447*PCALC + 0.666511*LONG - 0.405483*DP01SUM + 0.002143*AR - 0.205678*SLOP + 0.136601*DP01 - 0.585218*TNAUT + 0.052743*DTX25SUM - 0.582042*TXSUM

Polyommatus daphnis Y= - 38.909208 + 0.252613*LONG + 1.264914*CLAY - 0.022268*U500 + 1.389729*CALC - 2.928613*PGRAV - 0.234819*DTX25SPR + 0.061158*DTN0WIN + 0.916675*LAT + 1.381597*DP30SUM + 0.161164*SLOP - 0.007743*PET - 0.962901*DP10SUM + 0.052323*DTX25

Polyommatus dorylas Y= - 19.028975 + 0.495079*CALC - 0.009312*U500 + 0.003175*ALT + 0.007039*AET - 0.242021*DP1SUM - 0.128495*DTX25SPR - 13.521918*SIDSUM + 0.170675*SLOP + 0.609512*DP30SPR + 18.4058*SIDAUT + 15.740771*SISSUM + 0.125924*DP01SPR + 0.625011*DP10SUM - 0.027061*PSUM - 0.031475*DHI + 0.399353*CLAY - 26.089912*SISAUT - 0.0253*PWIN + 0.006257*PANN + 0.095134*DTN0SPR - 4.064601*TNJUL - 0.359421*LONG - 3.336993*TNAUT + 3.724802*TAUT - 0.078197*DTN0WIN - 2.565865*TWIN - 0.108372*DTN20 + 0.415382*DTN20AUT - 6.509069*TXSUM + 12.01808*TSUM + 0.941946*TNJAN

Polyommatus eros Y= - 377.487253 + 0.149317*DTN0SPR + 0.082129*PSUM + 6.012762*PCALC + 4.654337*SIL + 0.089335*SE - 0.530802*DTN20 + 3.049132*DTN20AUT - 1.617206*DP10SUM + 0.025545*AET + 6.72995*LAT + 0.640728*SLOP + 7.660902*SISSPR - 0.98382*DTX25SPR + 0.402915*DTX25AUT

Polyommatus escheri Y= - 38.773632 + 0.23654*LONG - 12.421123*SISAUT + 0.003044*AET - 0.008957*U500 + 0.001439*AR - 0.473316*DP30WIN + 10.860384*SISSUM - 0.029068*DHI + 0.012068*PSPR - 8.795452*SIDSUM + 11.021463*SIDAUT + 0.77527*DP30SUM + 0.104969*DTN0AUT + 0.005192*PET + 0.181797*DP01SPR - 0.294394*DP01SUM - 0.352827*CTI - 0.290408*DP10AUT + 0.430116*LAT - 0.59903*GYP - 1.013003*PSIL + 0.058753*DTX25SUM - 0.166118*DTX25AUT - 2.489751*TXJUL + 2.73979*TXSUM + 0.001338*ALT

Polyommatus fabressei Y=13.411558 + 0.003088*ALT - 3.572399*PSIL + 0.017972*U100 + 0.69737*LONG - 1.653909*SISSPR - 3.744621*PGRAV + 0.615861*GRAV - 0.042223*PSUM - 0.009575*PET + 0.491664*DTN20AUT + 0.001015*AR - 0.077985*DTN20

Polyommatus fulgens Y= - 233.135887 + 4.851424*LAT + 0.006827*AET - 0.783722*TXJAN - 0.025057*U500 + 3.103055*SISSUM + 0.069394*PSPR - 0.512996*DTX25SPR + 0.866153*TXSPR - 0.370878*DTN0AUT - 1.809924*PGYP + 0.069798*DTN0 - 0.015438*PANN + 0.391805*LONG - 1.002762*DP30WIN - 0.038001*DP1 + 0.001679*AR

Polyommatus golgus Y=61.331746 + 0.027602*ALT + 2.976672*TJUL - 1.315557*DTN20 + 52.33188*TNWIN - 3.889709*LAT + 48.845642*TXWIN - 100.889779*TWIN

Polyommatus hispanus Y= - 94.593763 - 2.144431*TNWIN - 0.742235*PSIL + 0.001972*AR - 0.008409*U500 - 8.580761*SIDSUM - 1.909577*SIDAUT + 13.364508*SISSUM + 0.963049*LAT + 0.09801*DP01WIN - 0.232096*DP01SUM - 0.134365*DTX25SPR + 0.074174*DTN0WIN + 0.005473*PET - 0.173014*DTN0SPR + 0.353106*CALC + 0.000445*HPD + 0.182095*DTN0AUT + 2.090543*TNJAN + 0.370149*DP10SUM + 0.027831*PANN - 0.051704*PSUM - 0.037078*PWIN - 0.555491*DP10AUT - 0.019981*PAUT + 0.533823*DP30SUM + 0.163486*TXWIN - 0.753254*DP30WIN + 0.387318*DP30

Polyommatus icarus Y=13.482447 - 0.015114*PSUM + 0.00217*AR + 0.00084*HPD + 5.148026*SIDAUT - 0.018649*DHI + 0.316209*CALC - 1.817078*TNSUM - 0.027242*PWIN + 0.002299*AET + 0.016248*PANN + 0.011505*SE + 3.921355*TSUM + 0.57518*TXJAN - 0.215662*DP10WIN + 0.133403*DTN0AUT + 0.820979*PGYP + 0.715146*TXAUT - 3.504114*TXANN + 0.514158*DP30AUT - 0.094481*DP01AUT + 1.788805*SIDWIN + 0.003535*PET - 0.322485*DP1SPR - 0.015726*PAUT + 0.212544*DP1WIN + 0.231364*DP01SPR + 0.558063*DP30WIN + 7.180144*SISSPR - 0.139404*DP01SUM - 19.298688*SIS - 1.313228*TJUL - 0.249075*DP30 - 0.009982*DTX25 + 0.30705*DP10SUM + 0.055754*DTN20AUT + 3.006072*SIDSUM - 0.046225*DTN0SPR + 1.530647*TANN + 0.087476*LONG

Polyommatus nivescens Y=28.241864 + 2.482516*SIDWIN + 0.772297*CALC - 1.731076*CTI + 0.051925*DTX25SUM - 0.014363*PET + 0.574503*GRAV - 0.721053*DP10SUM + 0.655612*LONG - 0.010119*U500 + 0.238789*DTN0SPR + 0.283645*DP01WIN - 0.190921*DP1 + 0.664408*DP30SPR - 0.218576*DTN0AUT + 7.12519*TSUM - 6.306382*TJUL - 4.714558*TAUT + 0.350003*DP1SPR - 2.304961*TSPR + 6.348781*TANN

Polyommatus ripartii Y= - 205.076323 + 4.979747*LAT + 0.28444*LONG + 4.086022*SISSUM + 0.665924*CALC - 0.641586*DTX25SPR - 0.02215*U500 - 0.605824*CTI - 0.011461*PANN + 0.041188*PSPR + 0.075835*DTX25 - 0.301729*DP1AUT - 0.01222*PET - 1.288921*DP30WIN - 0.630014*DTN0AUT + 0.002044*AR - 1.385766*PGYP + 0.296363*DP10WIN + 0.215103*DTN0 - 0.225506*DTN0WIN + 1.757128*TXSPR - 1.514737*TXANN - 3.864008*SISSPR + 1.009526*TNJUL - 1.258268*TNSPR

Polyommatus thersites Y= - 32.102298 - 0.012895*U500 + 0.143078*LONG + 0.004814*AET - 0.009366*U100 + 0.000838*HPD + 0.015967*SE + 0.351*CALC + 0.002099*AR - 0.158052*DTN20AUT - 0.180021*DTX25SPR + 0.218066*DTN0AUT + 0.381213*DP10SPR + 0.585631*LAT + 0.595255*DP01SPR - 0.345528*DP10WIN + 0.004971*PET - 0.166063*DP01SUM - 0.570585*DP1SPR + 0.309926*DP1WIN - 0.695593*PSIL + 0.144451*DTN0WIN - 0.420471*CTI + 3.179206*TJAN - 1.41698*TNWIN - 0.10482*DP01 - 1.73948*TXJAN + 0.002029*ALT - 0.123096*SLOP + 0.01182*PSPR - 3.143261*TXJUL + 3.596066*TXSUM - 0.07968*DTN0 - 0.007181*PWIN

Pontia callidice Y=110.085955 + 1.021601*DP10SUM + 0.005927*AR - 0.178314*DTX25 + 2.56003*TXWIN - 0.093854*DHI + 5.154026*LONG + 59.279366*SIDSUM - 3.376069*PSIL - 2.452186*TNJUL - 43.839204*SISSUM - 27.363488*SID

Pontia daplidice Y= - 12.616263 + 0.001804*AR + 4.316602*SIDSUM + 0.393267*LAT + 0.001023*HPD + 0.299497*GYP - 0.015606*DHI + 0.001013*AET - 0.094793*DP10WIN + 0.432865*SIL + 0.154196*DP01WIN + 0.467447*DP30SUM + 0.207103*LONG - 3.910837*SISSUM + 0.006315*SE - 0.416552*TXSPR - 0.04041*DP01 + 0.083583*DTN0AUT + 1.136086*TXSUM + 0.322734*TNJAN - 0.754129*TXJUL - 0.413582*PSIL - 0.386495*PCLAY - 0.142699*CTI

Pseudochazara hippolyte Y=117.587503 + 0.003589*AR + 0.052605*U500 - 3.0682*LAT - 0.913097*TXAUT

Pyrgus alveus Y=19.579929 - 4.629105*TANN + 0.340177*LONG + 0.007199*AET - 0.03664*DHI + 0.734733*CALC + 0.001587*AR - 0.007844*PET - 0.264474*DP01SUM - 8.971151*SIS + 1.018847*DP30SPR - 0.006992*PWIN + 0.330107*DP01SPR + 0.469887*SIL + 3.236841*TNSUM - 0.339589*DP1SPR + 0.180017*DP1WIN - 0.139366*DP01AUT + 1.323579*TXSPR - 2.196124*TNJUL + 0.002901*ALT + 1.99546*TAUT + 0.080454*DTX25SUM - 0.055978*DTX25 + 5.726829*SID

Pyrgus andromedae Y= - 991457.887909 - 6265.312928*DP10SUM + 6.748562*AR + 224.983074*PSUM + 287.259716*U100 + 664.167546*PAUT + 7679.950721*LONG + 99.624449*DHI - 278.495085*DTX25 - 7901.968964*DP30AUT - 521.978438*PSPR + 27231.787236*DP30SPR + 39868.984813*SISAUT + 18217.876096*SIL - 57359.531832*PGRAV - 11715.125795*DP30 + 27057.786899*PCLAY + 7298.720348*DP10SPR + 2542.690965*GRAV + 17831.135817*LAT - 6683.071109*SIDSPR

Pyrgus armoricanus Y= - 56.630331 - 1.477984*TXSPR + 0.005706*AET + 0.000719*HPD + 1.208981*LAT - 0.240184*DP1SUM + 0.001104*AR - 0.047207*DTN20 - 0.005367*U500 + 0.02801*DTX25SUM + 3.153573*TSUM + 0.411363*SIL + 0.406022*CALC + 0.77137*TXJAN - 2.126964*TJUL - 0.53208*TNWIN + 0.011434*PSPR - 0.266641*DP10AUT

Pyrgus bellieri Y= - 4.067592 + 0.006414*PAUT + 0.001496*HPD + 0.002176*AET + 0.002099*AR - 0.016931*DHI + 0.228689*LAT + 7.610285*SIDSUM - 7.930839*SISSUM - 1.762553*TNJUL - 2.918159*TXJAN + 0.561276*DTN0SUM + 1.099026*PGYP + 0.320745*SIL + 0.25507*CALC + 3.728319*TXWIN - 1.338637*TXSPR + 4.207111*TSUM - 0.014186*DTX25 + 0.007187*SE - 1.067858*TAUT + 0.85448*TSPR + 0.284425*DP30WIN + 0.229655*DP10SPR + 0.150406*DTN20AUT + 0.215119*LONG + 0.141791*DP01WIN - 0.050044*DP1 + 0.011107*PSUM - 0.243065*DP30 - 1.430429*TXJUL + 1.093055*SISSPR + 0.001025*ALT - 0.003819*PWIN - 0.030167*DTN20 - 0.015287*DTN0WIN + 0.02738*DTX25SPR - 0.21979*GRAV + 0.53126*PGRAV

Pyrgus cacaliae Y= - 3.145022 - 2.261075*TJAN - 7.017303*DTN0SUM

Pyrgus carthami Y= - 86.673844 - 1.597461*TXWIN + 0.007499*AET - 0.042618*DHI + 0.476682*CALC + 0.000787*HPD - 0.217619*DP01SUM + 1.199183*LAT + 0.00142*AR - 0.173432*DTX25SPR + 0.057707*DTX25SUM + 0.650474*DP01SPR + 0.076162*DTN0AUT + 0.679162*PCALC + 0.522886*DP1AUT - 3.552906*TJUL + 4.375969*TSUM + 0.829769*DP30SPR - 0.580476*DP1SPR - 0.549903*DP01AUT - 0.205832*DP10AUT - 7.259546*SIDWIN - 21.873783*SIDSUM + 17.934624*SISSUM - 0.38207*GRAV - 9.1432*SISSPR + 23.804133*SID + 1.515246*TJAN - 0.903491*TNAUT

Pyrgus cinarae Y= - 6.776602 + 0.086781*U100 + 2.06938*TXJAN - 2.434458*TANN

Pyrgus cirsii Y= - 41.391209 + 1.68859*TWIN - 0.009938*U500 + 0.008123*AET + 5.214916*TNSUM - 0.165277*DTX25SPR - 3.01338*TNAUT - 0.026042*DHI - 16.41608*SISSPR + 12.19058*SIDSPR - 0.392797*DP1SUM + 12.809445*SISSUM + 0.17077*DP01SUM - 9.999684*SIDSUM + 0.776503*LAT + 0.00332*ALT + 0.061088*DTN0AUT - 0.954624*TXJAN - 2.741121*TNJUL - 0.441106*SIL

Pyrgus malvoides Y= - 25.694134 + 0.417587*DP10SUM + 0.002412*AR - 0.004206*U500 + 0.000715*HPD + 0.37699*LAT + 0.336996*LONG + 0.060884*DP1WIN - 0.276259*DP1SUM + 0.102228*DP01SPR + 0.041176*DTX25SUM + 0.004135*AET + 0.032619*PSPR - 0.007809*PANN + 0.83849*SISSUM - 0.123155*SLOP + 0.331707*SIL - 0.089531*DTX25AUT - 0.13159*TXJUL

Pyrgus onopordi Y= - 8.785312 - 0.009595*U500 + 0.002617*AET + 0.000398*HPD - 2.108707*TAUT + 8.107643*SID + 2.861206*TNSUM + 0.74097*TXWIN - 5.828352*SIDAUT + 0.079598*DTN20AUT - 1.67748*TNJUL - 2.557449*SISSPR + 0.001342*AR + 0.315029*CTI + 0.535483*PCALC + 0.057756*DP01 + 0.657888*DP30AUT - 0.00949*PAUT + 0.007751*U100 + 0.0373*DTN0WIN - 0.181517*DP1SUM + 0.014257*WE + 0.034312*DTX25 - 0.105732*DTX25SPR

Pyrgus serratulae Y=8.949184 + 0.007128*AET + 0.266968*LONG - 0.049001*DHI - 1.67926*TXJAN - 6.604317*SISSPR - 0.144374*DP01SUM - 0.006719*PET + 0.001334*AR - 0.165854*DTX25SPR + 0.882768*DP30SPR - 0.65773*DP30WIN + 1.774471*SISSUM + 0.74082*DTN0SUM + 0.312228*DP01SPR + 0.038709*DTX25SUM + 1.859003*TXWIN - 0.20317*DP01AUT + 0.432631*DP30AUT + 0.808931*PCALC - 0.132097*DP1SPR - 4.026646*SISWIN + 0.003172*ALT + 5.404165*TSUM - 1.043212*TNANN - 3.189441*TJUL - 1.177834*TXSUM + 3.728111*SIDSPR

Pyrgus sidae Y= - 9.345332 + 0.003274*AR

Pyronia bathseba Y= - 31.074064 - 0.395259*TXJAN - 0.002905*U500 - 0.009198*U100 + 0.001545*AR + 0.146157*DP10 + 0.002879*AET - 0.066558*DTX25SPR + 0.016016*DTX25SUM - 0.016696*DHI + 0.000537*HPD + 1.309435*SISSUM - 0.529392*DP10WIN - 0.263967*DP01SUM + 0.139407*DP01WIN - 0.488985*CTI + 0.090531*DTN0AUT + 0.342342*DP30SPR - 0.738369*PSIL + 0.438445*SIL + 0.68517*LAT + 0.006886*PAUT + 0.185798*LONG + 0.375148*TWIN - 0.251307*DP10AUT - 0.574672*DTN0SUM - 2.01071*TJUL + 2.156677*TSUM + 0.200049*GRAV + 3.578957*SIDAUT - 2.307284*SIDSPR

Pyronia cecilia Y=2.65341 - 5.837778*SIDSUM + 0.199191*SLOP - 0.24261*DP10WIN + 0.002902*AET - 0.015815*DHI + 0.000618*HPD - 15.640013*SIDAUT - 1.336484*TNJUL + 0.006379*PSPR + 0.302198*SIL - 1.447516*TNAUT - 0.003921*PWIN + 0.007333*SE - 2.150983*TXWIN + 1.92928*TXJAN - 0.001551*ALT + 0.125024*DP30 + 0.044267*DTN20AUT + 0.170918*DP1WIN - 0.38426*DP01SUM + 0.244505*DP10SUM + 41.38812*SID - 8.16573*SISSUM + 0.317846*DP01SPR - 0.307325*DP1SPR - 0.107342*DP01WIN + 0.264987*DP1SUM + 4.117415*TANN - 2.647059*TXANN - 15.365212*SISWIN - 11.892603*SIDSPR + 15.424446*SIS + 0.034931*DTN0AUT + 2.111209*TSUM - 0.538199*TXJUL

Pyronia thitonus Y=4.664362 + 0.639073*TXANN + 0.003716*AET - 0.002591*U500 - 0.230051*DP10AUT + 0.001619*AR - 0.615453*TXSPR - 0.019188*DHI + 12.812908*SIDAUT + 0.022875*PSPR + 0.000534*HPD - 13.980917*SISWIN - 0.054592*DTX25SPR + 5.092552*SIDSUM + 3.395907*SISSPR + 0.25218*CALC + 0.216381*DTN0AUT - 0.11656*DTN0SPR - 0.664294*DTN0SUM + 0.016685*PAUT + 15.014556*SIDWIN - 0.163841*DP10WIN - 17.384129*SID + 0.404486*DP01SPR + 0.101625*SLOP + 0.253939*CTI - 0.034743*DTX25AUT - 6.363923*SISAUT - 0.104543*DP01 - 0.280042*DP1SPR + 0.242824*DP1WIN + 0.293601*DP30 - 0.389508*DP30WIN - 0.00932*PANN + 0.198788*DP10SUM

Satyrium acaciae Y= - 42.435409 - 0.011633*U500 + 1.384843*LAT - 0.159333*DTX25SPR + 0.546929*DP30SPR - 0.356699*DP01SUM + 0.412791*LONG - 6.025046*SISSPR + 7.148448*SID + 0.001388*AR + 0.034496*PSPR + 0.686261*DTN0SUM - 0.007806*PET + 0.0032*AET + 0.140219*DP01SPR + 0.014017*PSUM - 0.011958*PANN

Satyrium esculi Y=3.214811 + 0.004008*AET - 0.359312*DP10WIN + 0.00188*AR + 0.000549*HPD + 2.1243*TXJAN + 3.50521*TANN - 2.783815*TXWIN + 10.580493*SID - 14.715163*SIS + 0.004419*PET + 0.23238*DP10AUT + 0.156215*DTN0AUT + 0.209098*DP01WIN - 0.478292*DP1SPR + 0.599634*DP01SPR + 0.26455*DP1WIN + 0.621355*PCALC + 0.349748*SIL - 0.016423*PWIN + 0.811616*DP30WIN - 0.211831*DP01 + 0.007432*PANN - 0.419246*DP30 + 0.464842*DP30AUT - 0.057505*DTN0SPR - 1.767387*TSPR + 0.313592*LAT + 0.184471*GRAV + 0.411406*DP30SUM - 1.278044*TAUT + 2.067824*TWIN - 1.681977*TJAN - 0.234221*TNJUL + 2.216329*SISSPR + 0.007435*SE

Satyrium ilicis Y=50.293229 - 0.663192*TXANN - 0.004335*U500 + 0.006113*AET + 0.002101*AR - 0.27256*DTN20AUT - 0.20633*DP10WIN + 25.686932*SISWIN + 0.000554*HPD - 0.25722*DP1 + 0.006388*PSPR + 9.367753*SIDSUM - 32.403999*SIS + 0.38149*CALC + 0.493031*LONG + 0.613264*DP1WIN - 0.360923*TJUL + 0.25537*DP01SPR + 0.638748*TXSPR + 0.204444*DTN0AUT - 0.118411*DTN0SPR - 0.015629*PSUM - 0.005962*PET - 12.066326*SIDWIN + 5.64617*SIDSPR + 0.505872*DP10SUM - 0.909329*DP30WIN + 0.486513*DP30 - 0.139133*DP01WIN

Satyrium pruni Y=29.608393 - 14.790028*SISWIN + 0.952996*LONG - 0.866619*DP10SUM

Satyrium spini Y= - 12.140962 + 0.001575*AR - 0.004564*U500 + 0.438881*CALC + 0.000417*HPD + 0.053642*DTX25SUM - 2.080219*SISSPR - 0.378732*DP01SUM - 2.007263*TJUL + 0.190106*LONG + 0.075904*DP01 + 0.435201*LAT + 2.746479*SIDAUT + 1.916316*TSUM + 0.048344*DTN0SPR - 0.024043*DTX25 - 0.187834*DP1AUT + 0.01018*PSPR - 0.006711*PWIN + 0.416881*DP30AUT + 0.301525*SIL

Satyrium w-album Y= - 150.510135 + 0.692655*DP10SUM + 2.837302*LAT + 0.615472*LONG - 0.246738*DTX25SPR - 0.393649*DP1SUM - 0.237243*DTN20 + 0.00188*AR + 0.404948*DP30 + 0.75295*DTN20AUT - 0.231386*DP10 + 0.05524*DTX25SUM + 1.172574*CTI - 0.056013*DTN0AUT + 0.934972*DTN0SUM + 0.157052*SLOP + 7.216564*SISWIN - 14.712485*SIDAUT + 6.811914*SID + 0.372175*DP1SPR

Satyrus actaea Y= - 61.429767 + 0.001321*AR - 9.510417*SISSPR + 10.868565*SISSUM - 0.124483*DTX25SPR + 0.400486*CALC - 0.006531*U500 - 9.302323*SIDAUT - 7.030428*SIDSUM + 0.04873*DTX25SUM + 0.883432*LAT - 0.178911*DP1WIN + 0.302109*DP01SPR - 0.325775*DP01SUM + 0.021354*PANN - 0.025504*PWIN + 1.398524*TXJUL + 0.806295*TXSPR + 1.236852*TNJAN - 3.639385*TAUT - 0.028057*PSUM - 0.027077*PSPR + 13.21781*SISAUT + 0.001698*ALT + 0.79994*PCALC - 0.376297*CTI + 1.180426*PGRAV + 0.730422*PSIL - 0.157696*DP10WIN + 3.017585*TNSUM - 3.75793*TJUL + 1.791067*TXAUT + 3.917265*SIDSPR - 0.909057*TJAN

Satyrus ferula Y= - 408.135022 + 0.278144*DTN0WIN + 0.021959*AET + 7.921242*LAT + 2.404844*TNSPR + 1.724262*LONG - 3.332302*DP30SUM + 0.171931*DTN0SPR + 0.058782*PAUT - 1.766678*DP30 + 0.002009*AR + 8.798402*PGRAV + 3.518051*SIDSUM + 4.290158*DP30SPR

Scolitantides abencerragus Y=144.709151 - 2.135502*LAT - 2.734168*TAUT + 1.460731*CALC - 0.813986*DP10SUM + 30.868994*SID - 0.715019*CTI + 0.891823*GRAV + 1.162044*TJAN - 17.158284*SIDSPR + 4.478303*TSUM - 3.448339*TJUL - 19.128231*SIDAUT - 28.006747*SISSUM + 13.582234*SISSPR - 0.024411*DHI + 19.154988*SIDSUM + 0.874006*LONG - 0.607865*DP01AUT + 0.214676*DP01

Scolitantides baton Y= - 64.274676 + 1.279586*LAT - 0.005012*U500 + 11.061344*SISSUM - 0.117878*DTX25SPR - 1.221305*PCLAY + 0.000507*HPD - 10.565839*SIDSUM + 0.56922*SIL - 0.061142*DTN20 + 24.303384*SIDAUT + 0.040895*DP01 - 10.48676*SIDWIN - 23.154819*SISAUT + 1.870089*TNSPR - 1.308341*TNJUL - 2.0895*TWIN + 0.04549*DTX25SUM + 0.331382*DP30WIN + 12.891421*SISWIN + 0.002967*AET + 0.182449*SLOP - 0.364567*DP1SUM - 0.420494*DP10AUT + 0.451657*DP10SUM + 1.835743*TAUT + 2.595467*TNJAN - 2.605449*TNWIN - 0.36042*TXSUM

Scolitantides orion Y= - 20.385188 + 0.679339*LONG - 0.100177*DTX25AUT + 1.002128*CALC + 0.002844*AET - 0.181283*DP10SPR - 19.55226*SIDAUT + 5.119088*SISSUM + 16.427975*SISAUT - 0.010014*U500 - 6.194068*SISSPR + 0.441071*TXSPR - 1.305126*PSIL + 0.001711*AR + 0.043223*DTN20 - 0.007888*PET

Scolitantides panoptes Y= - 14.491002 - 0.017695*U100 + 0.053324*DTX25SUM - 0.093684*DTX25SPR - 0.004904*U500 - 0.820838*CTI + 0.446349*LAT + 0.276651*DP10SUM + 0.000346*HPD + 0.471524*CLAY + 0.427076*SIL + 0.51252*DP01SPR - 0.174039*DP1 + 0.021501*DTN0 + 3.476016*TANN - 1.296134*PSIL - 1.509929*TSPR - 1.620953*TAUT + 0.00188*AR - 0.194915*SLOP - 0.12365*DP10 + 0.396269*DP1AUT - 0.164311*DP01 + 0.214882*DP01WIN + 0.00396*PANN - 0.881698*PCLAY - 0.613076*PCALC

Spialia sertorius Y=22.680072 - 0.5254*CTI + 1.829388*TXJAN + 0.393603*TAUT + 0.002204*AET + 0.000514*HPD - 2.260232*TXWIN + 0.001203*AR + 0.004328*PET + 0.397496*DP01SPR + 0.113892*DTN0AUT + 0.418294*SIL - 0.426825*PSIL - 0.304882*DP10WIN - 0.383941*DP01AUT + 0.84929*DP30WIN - 0.010583*PWIN + 0.014221*PSUM + 0.054647*DTX25SUM - 0.08744*DTX25AUT + 0.292637*DP1AUT - 0.433559*DP1SPR + 0.320591*CLAY + 0.249644*DP1WIN + 0.012528*PSPR + 0.139189*DTN20AUT - 0.429422*PCLAY + 0.492234*DP30AUT - 0.337186*DP30 - 5.676087*SIDSPR + 20.891324*SIDAUT - 22.352767*SISAUT - 0.004059*U100 - 0.050804*DTN0SPR - 0.339302*DP1SUM + 5.621421*SISSPR + 0.494853*DP30SUM - 0.023909*DTN20

Tarucus teophrastus Y= - 329.915747 + 0.033018*PET + 0.035485*U500 - 3.747026*DP10AUT + 0.232956*PSUM + 1.655773*DP10WIN + 1.726033*TNWIN + 50.742877*SISWIN - 70.339854*SID + 49.127148*SISSUM + 0.240342*DTX25AUT

Techla betulae Y= - 30.77828 + 0.291965*DP10SUM - 0.027004*PET + 0.015817*AET + 0.443639*LONG + 0.880753*LAT + 0.001994*AR - 0.258949*DP1SUM - 0.001119*ALT + 0.057003*DP01SPR - 0.249322*DTX25SPR + 0.144959*DTX25AUT

Thymelicus acteon Y=2.386451 + 0.001999*AR + 0.002924*AET + 0.000583*HPD - 15.730448*SISWIN + 4.854186*SIDAUT - 0.019376*DHI + 9.100662*SIS - 0.010841*PWIN + 0.286686*SIL + 0.016343*PSPR - 2.641444*SISSUM - 1.405264*TXSPR + 8.643808*SIDWIN + 6.226526*TSUM + 0.004581*PET - 3.370772*TXSUM + 5.384736*TANN + 0.479674*DP10SUM + 0.262848*CALC - 0.13102*DP1SUM + 0.196003*DP01WIN - 0.180811*DP01AUT - 0.236072*DP10WIN + 0.849979*DP30WIN + 0.064143*DTN0AUT - 6.882981*SISAUT - 0.355894*DP30 - 4.108579*TNSUM + 0.009474*SE + 0.32285*DP30AUT - 0.880299*TAUT - 0.813051*TNJAN - 2.143699*TXWIN + 1.408375*TXJAN

Thymelicus lineola Y= - 5.424275 - 2.942734*TXWIN - 0.006411*U500 + 0.003065*AET + 0.000693*HPD - 0.186821*DP10AUT + 0.002469*AR + 2.462223*TXJAN - 0.23311*DP1SUM - 8.306731*SISWIN + 7.105449*SIDAUT + 0.569014*DP30SUM + 0.10268*DP01SPR + 0.013758*SE + 0.151602*LONG + 0.351031*LAT - 0.416976*CTI - 0.18356*SLOP - 0.17169*DTN0SPR + 0.073435*DTN0 + 0.261046*CALC + 0.500183*TJAN

Thymelicus sylvestris Y=19.479345 - 2.047575*TXWIN + 0.005123*AET - 1.218168*TAUT + 0.000579*HPD + 5.036301*SISSPR + 23.16192*SIDAUT - 23.614701*SISAUT - 0.017402*DHI - 3.858389*SIDSPR + 0.00101*AR + 0.30419*DP01WIN + 0.29203*CLAY + 1.527032*TXJAN + 1.9676*TXAUT + 1.089652*TNSPR - 0.782159*TXSPR + 0.704989*DP01SPR + 0.007016*PSUM - 0.636443*DP10WIN + 0.19371*DP10 - 0.274494*DP01 - 0.388062*DP1SPR + 0.094235*DTN20AUT - 0.643516*TJAN + 0.218717*DP1WIN - 0.072602*DTX25AUT + 0.002832*PET + 0.222155*DP30WIN - 0.229502*CTI - 2.812099*SID + 0.020405*DTX25SUM + 0.344185*SIL - 0.402175*PSIL

Tomares ballus Y= - 7.318633 + 1.039346*TNJUL + 0.000851*HPD + 0.348299*DTN0AUT + 6.63734*SIDSUM - 14.956448*SIDAUT + 8.079499*SISWIN - 0.009417*U100 - 6.077617*SISSUM - 0.034268*DTX25AUT - 0.012464*DHI + 0.036528*PSUM - 0.65101*DP10SUM + 6.378177*SID + 0.497558*PCLAY - 0.058183*DTN20 + 0.21002*DTN20AUT - 0.406598*DP01SUM - 1.097826*TNSPR + 1.062151*TJAN + 0.196735*DP01WIN - 0.215365*DP1SPR - 0.182925*DP10WIN + 0.01204*PSPR + 0.342155*DP1SUM - 0.113852*DTN0SPR - 0.466866*TXWIN

Vanessa atalanta Y= - 1.966898 + 0.01322*PSPR + 0.001245*HPD + 0.001801*AR + 0.002338*AET - 0.006534*PWIN + 0.547765*SIL - 0.015042*DHI + 0.130286*DP01WIN - 2.120071*TJUL - 3.363536*SIDWIN + 8.903517*SIDAUT + 0.330972*TNAUT - 9.339885*SISAUT - 1.457698*TSPR + 1.548652*TANN + 0.095012*DTN0AUT - 0.132949*DP01AUT - 0.494407*GYP + 1.625541*TXJUL - 0.032602*DTX25AUT + 0.502048*PGRAV - 0.002887*U100 - 0.130852*DP10WIN + 1.173024*SISSPR + 0.162154*CLAY + 2.192172*TSUM - 1.574815*TXSUM + 0.136545*DP30WIN + 0.451565*DTN0SUM

Vanessa cardui Y=10.217665 + 0.00763*PSUM + 0.001196*HPD + 0.001755*AR - 3.130092*SIDWIN + 17.598243*SIDAUT + 0.285472*TXSUM + 0.221669*DTN0AUT - 19.163516*SISAUT - 0.23893*DP10WIN - 0.014078*DHI + 0.00224*AET + 0.239333*TNJAN - 1.030773*TXSPR + 0.145321*DP1WIN - 0.27623*DP01AUT + 3.685747*SISSPR + 0.346591*DP01SPR + 0.011806*SE + 5.390226*TANN - 1.053165*SIDSUM - 0.097147*DTN0SPR - 0.006213*U100 + 0.002481*U500 + 0.312698*DP30WIN + 0.287952*DP10SUM + 0.293951*SIL + 0.202275*CLAY - 0.231794*DP01SUM - 0.318814*DP1SPR + 0.186181*CALC + 0.703496*PGYP + 0.192779*DP1AUT - 2.116785*TXANN - 2.511596*TNANN + 0.009857*PSPR - 0.006443*PWIN

Vanessa virginiensis Y= - 0.962113 + 0.000472*HPD + 0.312359*DP01WIN - 0.188592*TXSUM + 1.12811*DP10SUM - 0.973129*DP30WIN - 0.053841*PSUM - 0.193521*DP1 + 0.656005*DP10AUT + 0.025718*PSPR

Zegris eupheme Y= - 14.495769 - 0.026339*U100 + 2.889201*SISSUM - 3.134954*SIDAUT + 0.000291*HPD + 0.167659*SLOP + 3.052718*TSUM + 0.015043*SE - 1.147198*TAUT - 2.315106*TJUL + 0.987079*DP10AUT + 0.386816*CALC - 0.379079*DP10 - 1.830225*TNAUT + 0.257948*DP01SPR + 2.210571*TNANN - 0.066871*DP01

Zerynthia rumina Y= - 11.356622 + 0.003648*AET + 5.659886*SIDSUM + 0.001167*AR + 0.274198*LAT + 0.08529*DTN20AUT + 0.000801*HPD + 0.18988*DP01SPR + 0.341917*SIL + 0.432575*CLAY - 0.255356*CTI - 5.592273*SISSUM - 0.008032*PWIN - 5.137647*SIDAUT + 0.445026*DP30WIN +

0.35905*PCALC + 4.528288*SIS + 0.021125*PSUM - 0.160068*DP01SUM - 0.163641*DP1SPR - 0.253966*DP10SUM + 0.087626*TJUL + 0.017214*DTN0 + 0.188552*TNJAN

Zizeeria knysna Y= - 7.486388 + 0.782346*GRAV - 1.10022*DP10SUM + 1.395621*DP30AUT + 0.000467*HPD - 0.59205*DP30WIN - 1.290933*DP30SUM - 2.336424*TAUT + 2.096821*TANN + 1.144065*PGRAV + 0.495778*SIL - 0.035073*DTN0WIN + 0.011906*SE + 0.019056*PSPR - 0.345628*DP1SPR + 0.244863*DP01AUT + 1.67594*SIS + 1.307718*DTN0SUM

Table S3. Species butterflies with metapopulation structure.

**Fam. Hesperiidae**	*E. palarica* Chapman, 1903
Carcharodus flocciferus Zeller, 1847	*E. pandrose* (Borkhausen, 1788)
C. tripolinus Verity, 1925	*E. triaria* (Prunner, 1798)
Carterocephalus palaemon (Pallas, 1771)	*E. zapateri* Oberthür, 1875
Erynnis tages (L., 1758)	*Hipparchia fagi* (L., 1763)
Hesperia comma (Linnaeus, 1758)	*Hyponephele lycaon* (Kühn, 1774)
Heteropterus morpheus (Pallas, 1771)	*Lasiommata maera* (L., 1758)
Ochlodes venata (Bremer & Grey, 1853)	*Libythea celtis* (Laicharting, 1782)
Pyrgus alveus (Hübner, [1803])	*Limenitis camilla* (L., 1764)
P. bellieri (Oberthür, 1910)	*L. reducta* (Staudinger, 1901)
P. carthami (Hübner, 1813)	*Melitaea cinxia* (L., 1758)
P. cirsii (Rambur, [1840])	*M. athalia* (Rottemburg, 1775)
P. malvoides (Elwes&Edwards, 1897)	*M. aetherie* (Hübner, [1826])
P. serratulae (Rambur, [1840])	*M. diamina* (Lang, 1789)
P. sidae (Esper, [1782])	*M. parthenoides* Keferstein, 1851
**Fam. Papilionidae**	*M. trivia* (Dennis & Schiffermüller, 1775)
Parnassius apollo (L., 1758)	*Melanargia galathea* (L., 1758)
**Fam. Pieridae**	*M. russiae* (Esper, 1783)
Anthocharis cardamines (L., 1758)	*Minois dryas* (Scopoli, 1763)
Colias alfacariensis (Ribbe, 1905)	*Nymphalis antiopa* (L., 1758)
C. phicomone (Esper, [1780])	*Pseudochazara hippolyte* (Esper, 1784)
Colotis evagore (Klug, 1829)	**Fam. Lycaenidae**
E. ausonia (Hübner, 1803)	*Aricia montensis* Verity, 1928
E. tagis (Hübner, [1804])	*A. morronensis* Ribbe, 1910
Gonepteryx rhamni (L., 1758)	*A. nicias* (Meigen, 1829)
Leptidea reali Reissinger, 1989	*Callophrys avis* (Chapman, 1909)
L. sinapis (L., 1758)	*Cupido alcetas* (Hoffmannsegg, 1804)
Pieris ergane (Hübner, [1813])	*C. argiades* (Pallas, 1771)
P. mannii (Mayer, 1851)	*C. lorquinii* (Herrich-Schäffer, 1847)
P. napi (L., 1758)	*C. minimus* (Fuessly, 1775)
Zegris eupheme (Esper, [1805])	*C. osiris* (Meigen, 1829)
**Fam. Nymphalidae**	*Cyaniris semiargus* (Rottemburg 1775)
Aglais io (L., 1758)	*Eumedonia. eumedon* (Esper, [1780])
A. urticae (L., 1758)	*Hamearis lucina* (L., 1758)
Apatura iris (L., 1758)	*Iolana debilitata* (Schultz, 1905)
Aphantopus hyperantus (L., 1758)	*Lycaena alciphron* (Rottemburg, 1775)
Araschnia levana (L., 1758)	*L. bleusei* Oberthür, 1884
Arethusana arethusa (Denis & Schiffermüller, 1775)	*L. hippothoe* (L., 1761)

Argynnis adippe (L., 1767)
A. aglaja (L., 1758)
A. illia (Denis & Schiffermüller, 1775)
A. niobe (L., 1758)
A. paphia (L., 1758)
Brenthis daphne (Denis & Schiffermüller, 1775)
B. hecate (Denis & Schiffermüller, 1775)
B. ino (Rottemburg, 1775)
Boloria dia (L., 1767)
B. euphrosyne (L., 1758)
B. pales (Denis & Schiffermüller, 1775)
B. selene (Denis & Schiffermüller, 1775)
Chazara briseis (L., 1764)
C. prieuri (Pierret, 1837)
Coeonympha arcania (L., 1761)
C. glycerion (Borkhausen, 1788)
Danaus chrysippus (L., 1758)
D. plexippus (L., 1758)
Erebia epiphron (Knoch, 1783)
E. epistygne (Hübner, [1824])
E. euryale (Esper, [1805])
E. gorge (Hübner, [1805])
E. gorgone (Boisduval, [1833])
E. hispania Butler, 1868
*E. lefebvre*i (Boisduval, 1828)
E. manto (Denis & Schiffermüller, 1775)
E. meolans (Prunner, 1789)
E. neoridas (Boisduval, 1828)
L. tityrus (Poda, 1761)
L. virgaureae (L., 1758)
Phengaris alcon (Denis & Schiffermüller, 1775)
P. arion (L., 1758)
P. nausithous (Bergsträsser, [1779])
Plebejus hespericus (Rambur, 1839)
P. idas (L., 1761)
Polyommatus amandus (Schneider 1794)
P. coridon (Poda, 1761)
P. damon (Denis & Schiffermüller, 1775)
P. daphnis (Denis & Schiffermüller, 1775)
P. dorylas (Fruhstorfer, 1910)
P. escheri (Hübner, [1823])
P. fabressei (Oberthür, 1910)
P. fulgens (Sagarra, 1926)
P. nivescens (Keferstein, 1851)
P. ripartii (Freyer, 1830)
P. thersites (Cantener, 1834)
Scolitantides abencerragus (Pierret, 1837)
S. baton (Bergsträsser, [1779])
S. orion (Pallas, 1771)
Satyrium acaciae (Fabricius, 1787)
S. ilicis (Esper, 1779)
S. pruni (L., 1758)
S. w-album (Knoch, 1792)
Thecla betulae (L., 1758)

Family Hesperiidae

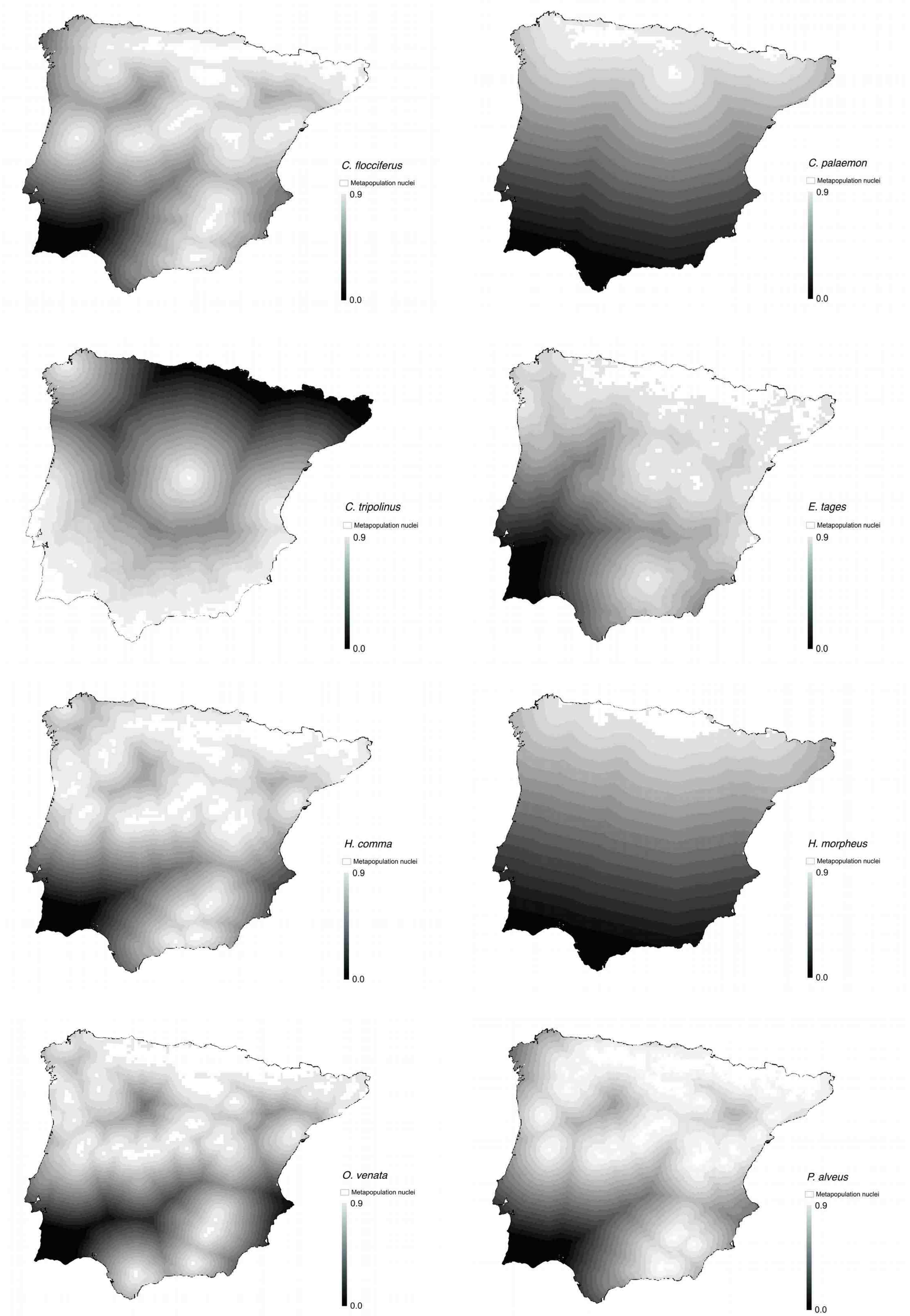

Family Hesperiidae

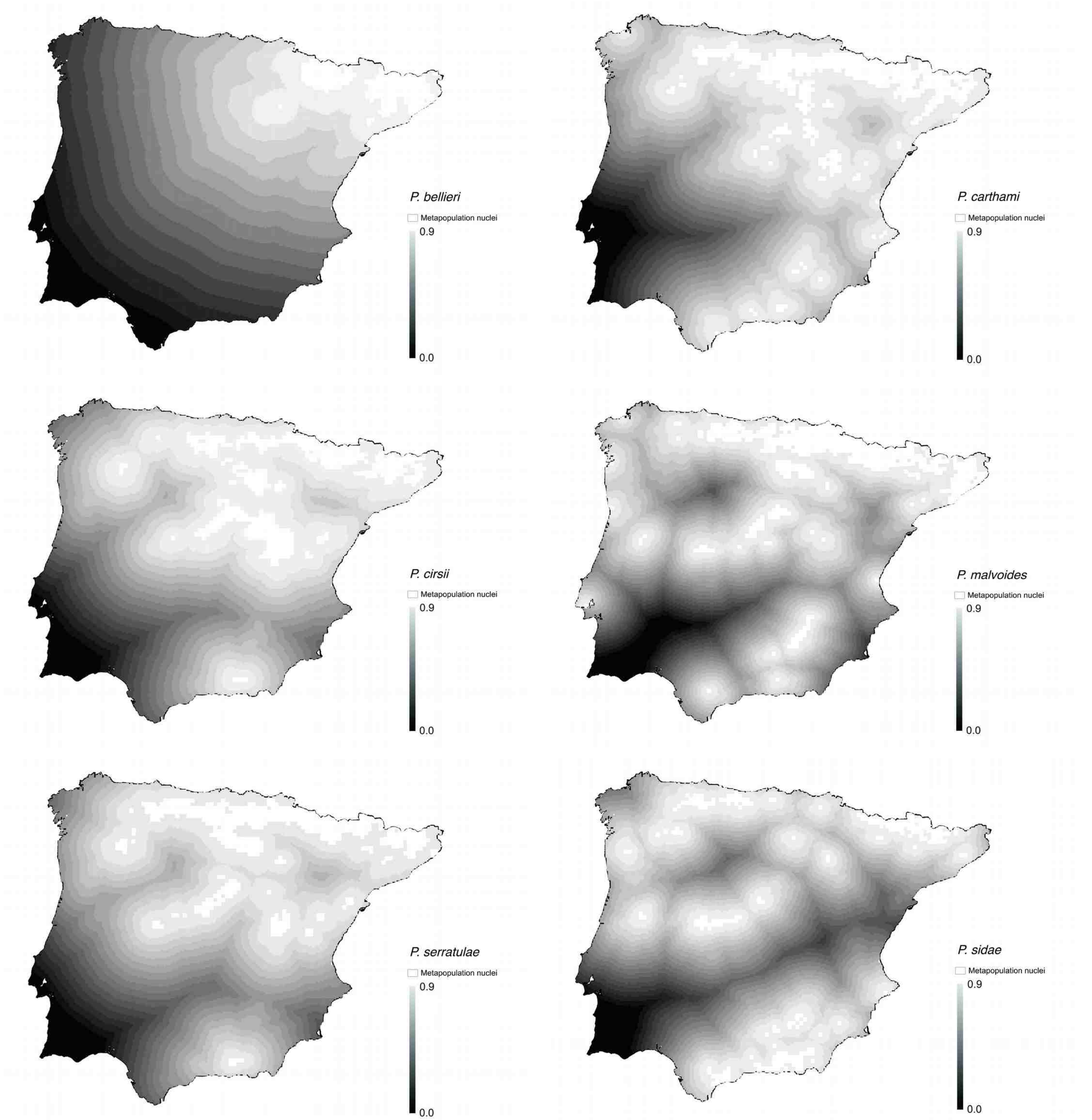

Family Papilionidae

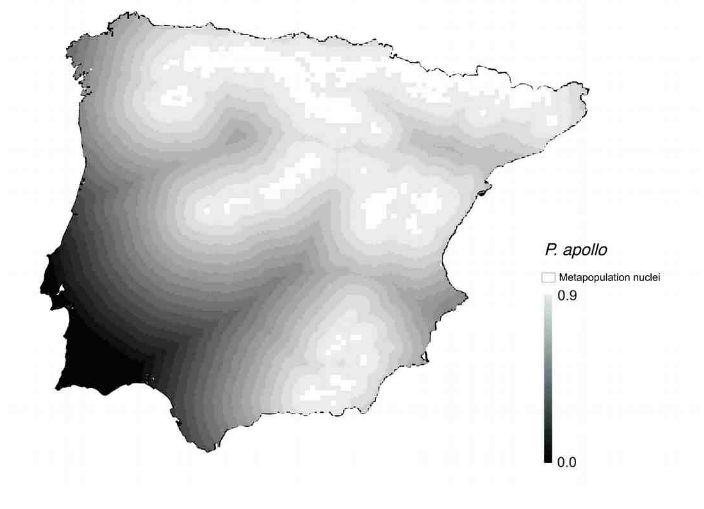

Family Pieridae

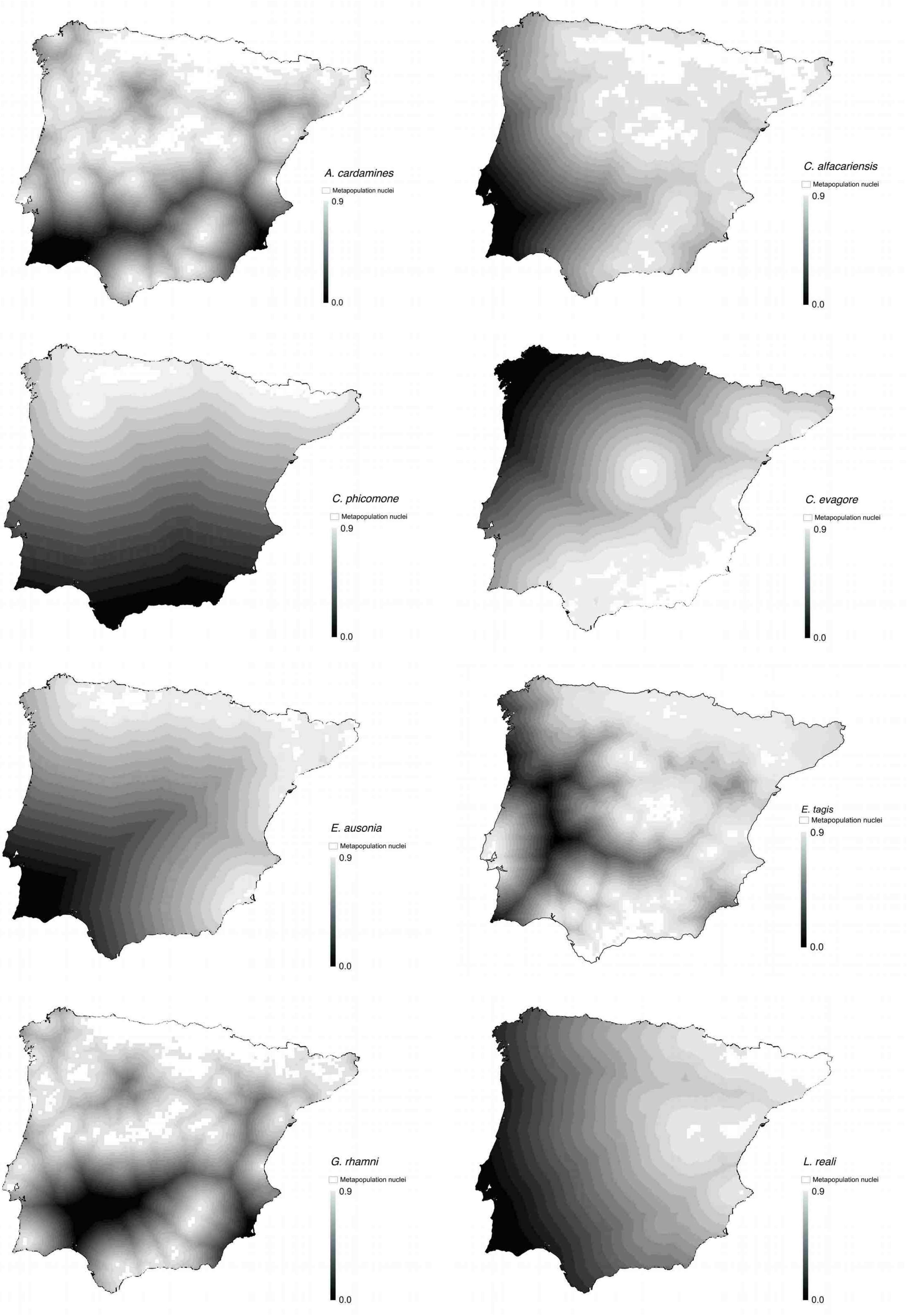

Family Pieridae and Nymphalidae

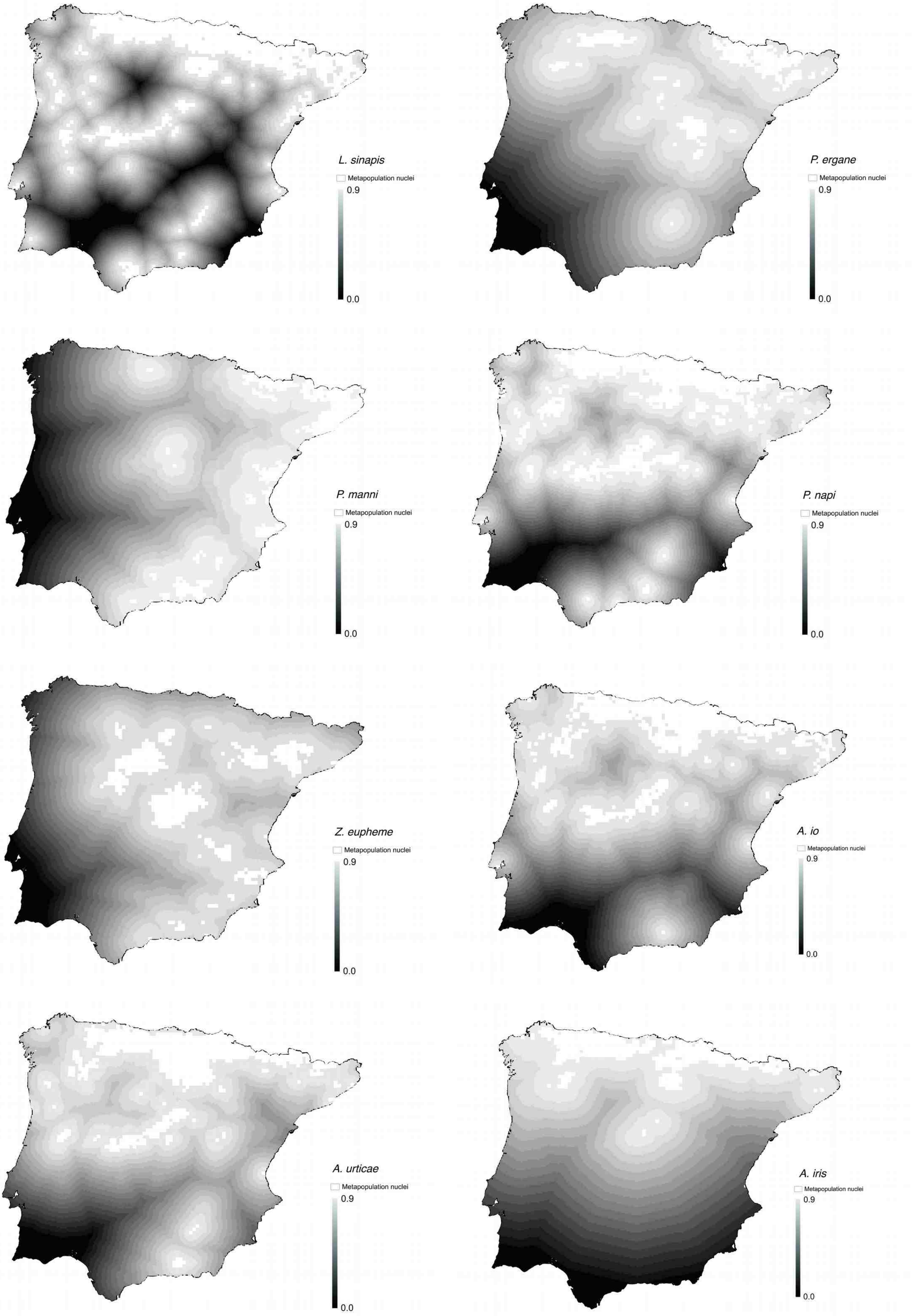

Family Nymphalidae

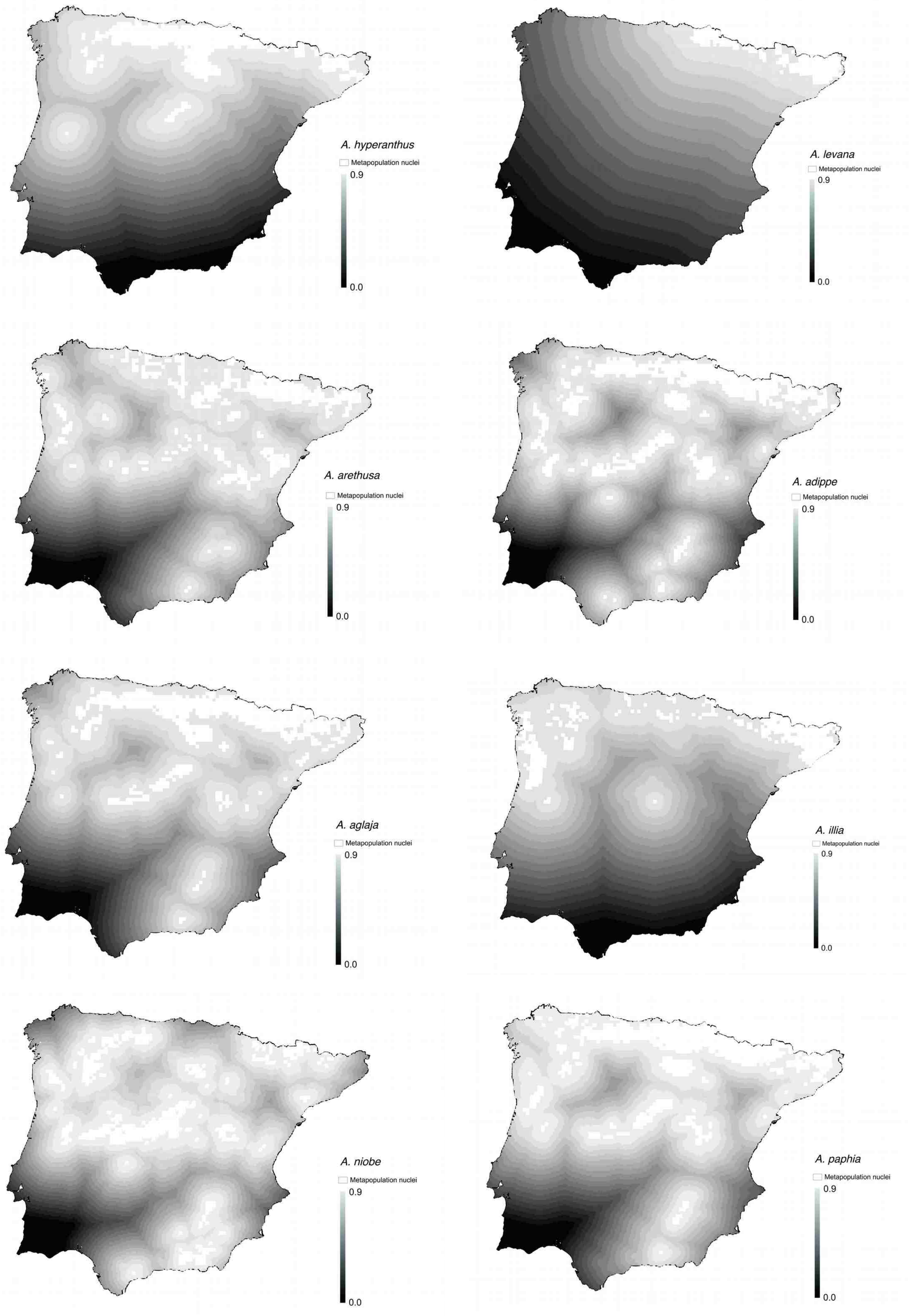

Family Nymphalidae

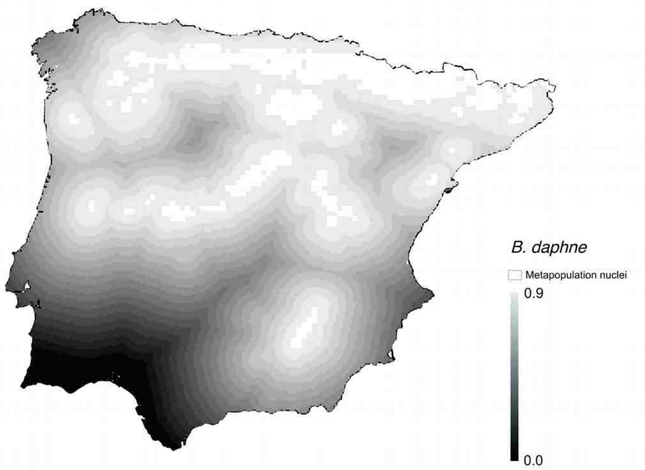

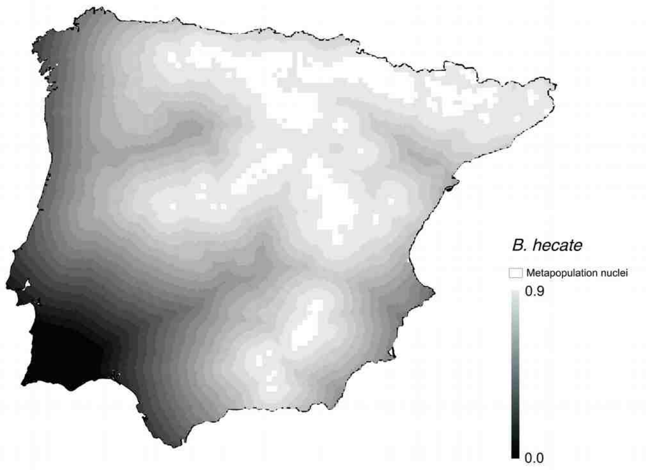

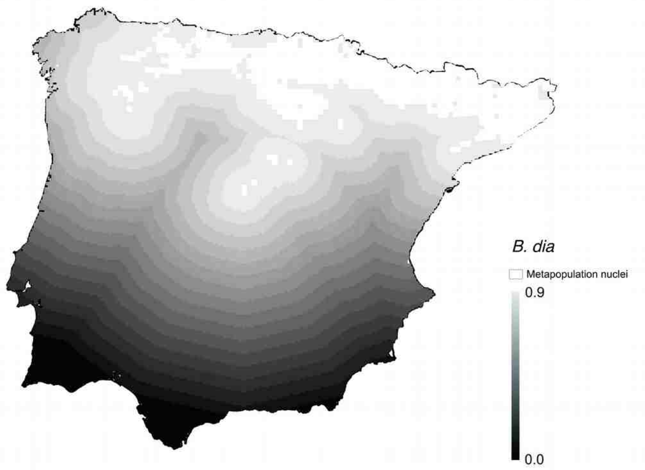

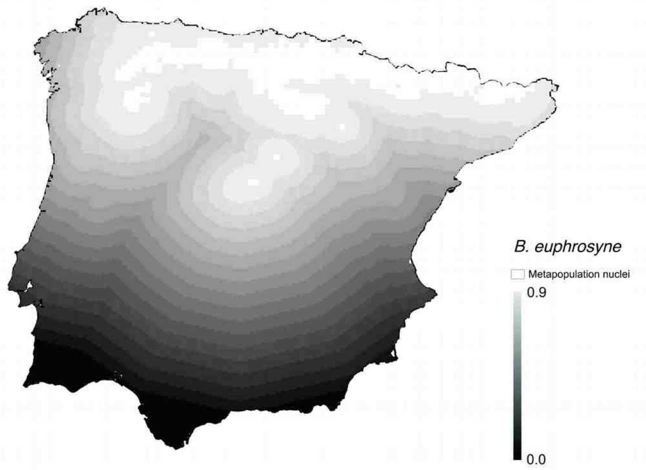

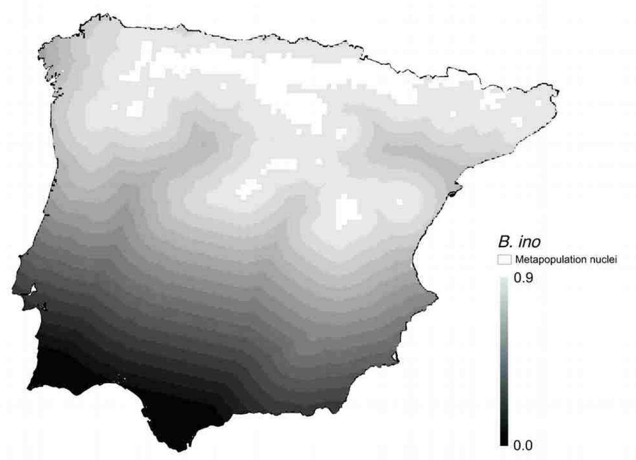

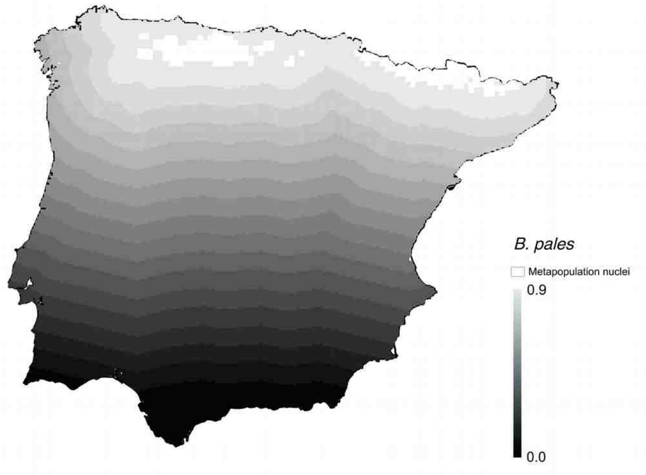

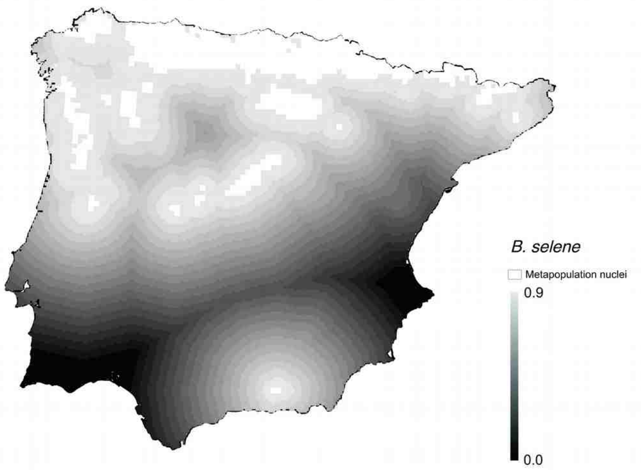

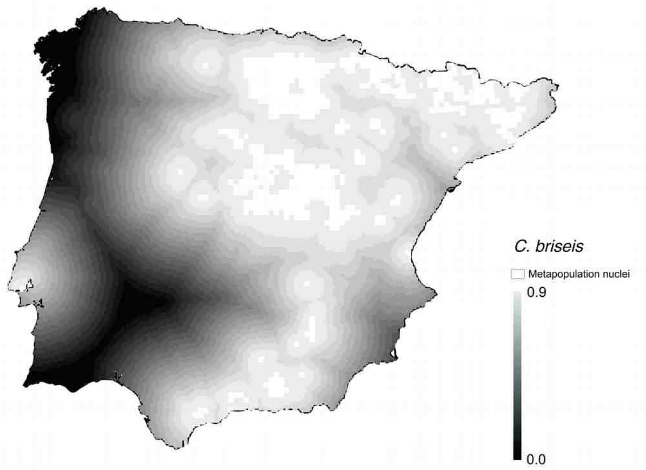

Family Nymphalidae

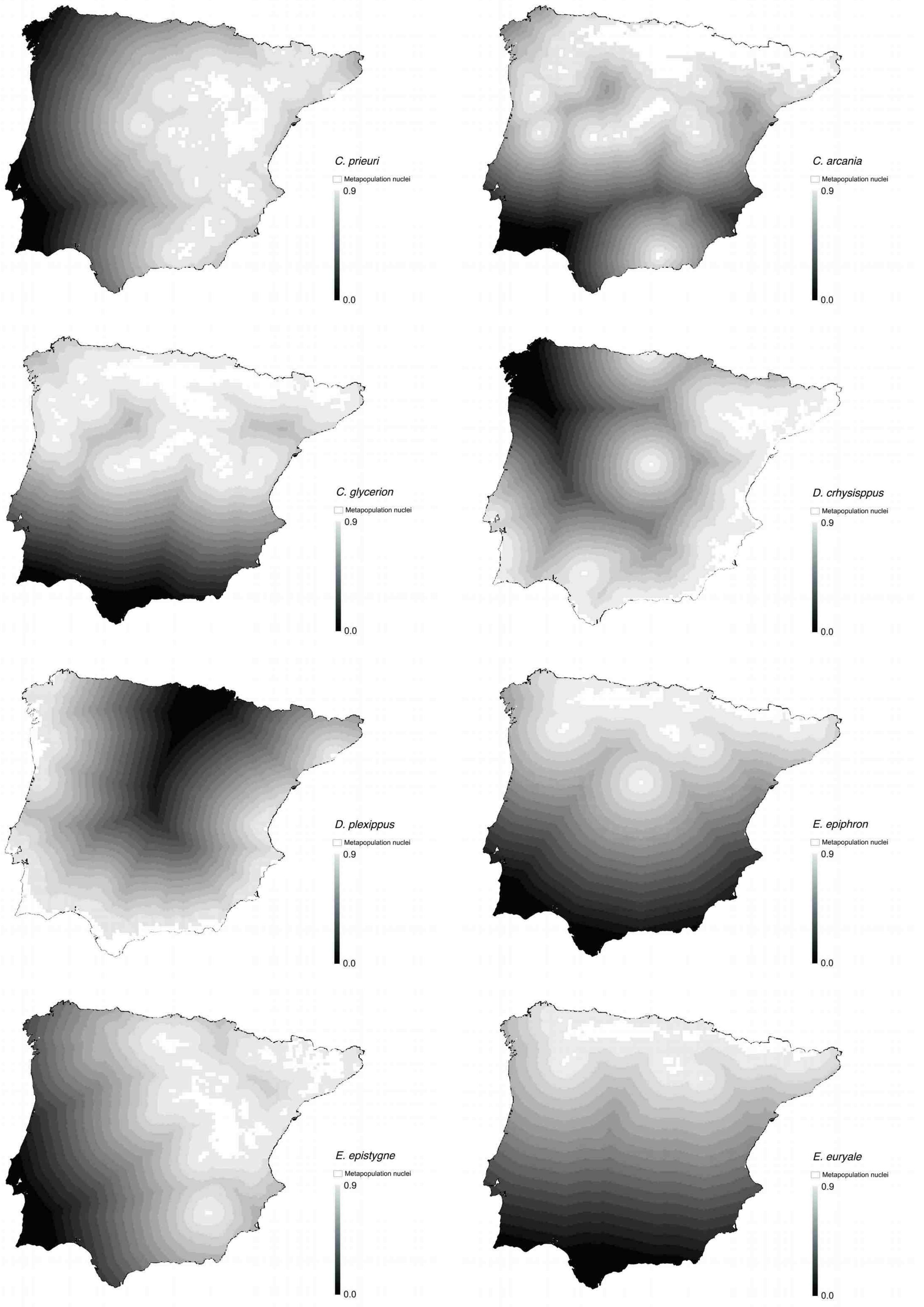

Family Nymphalidae

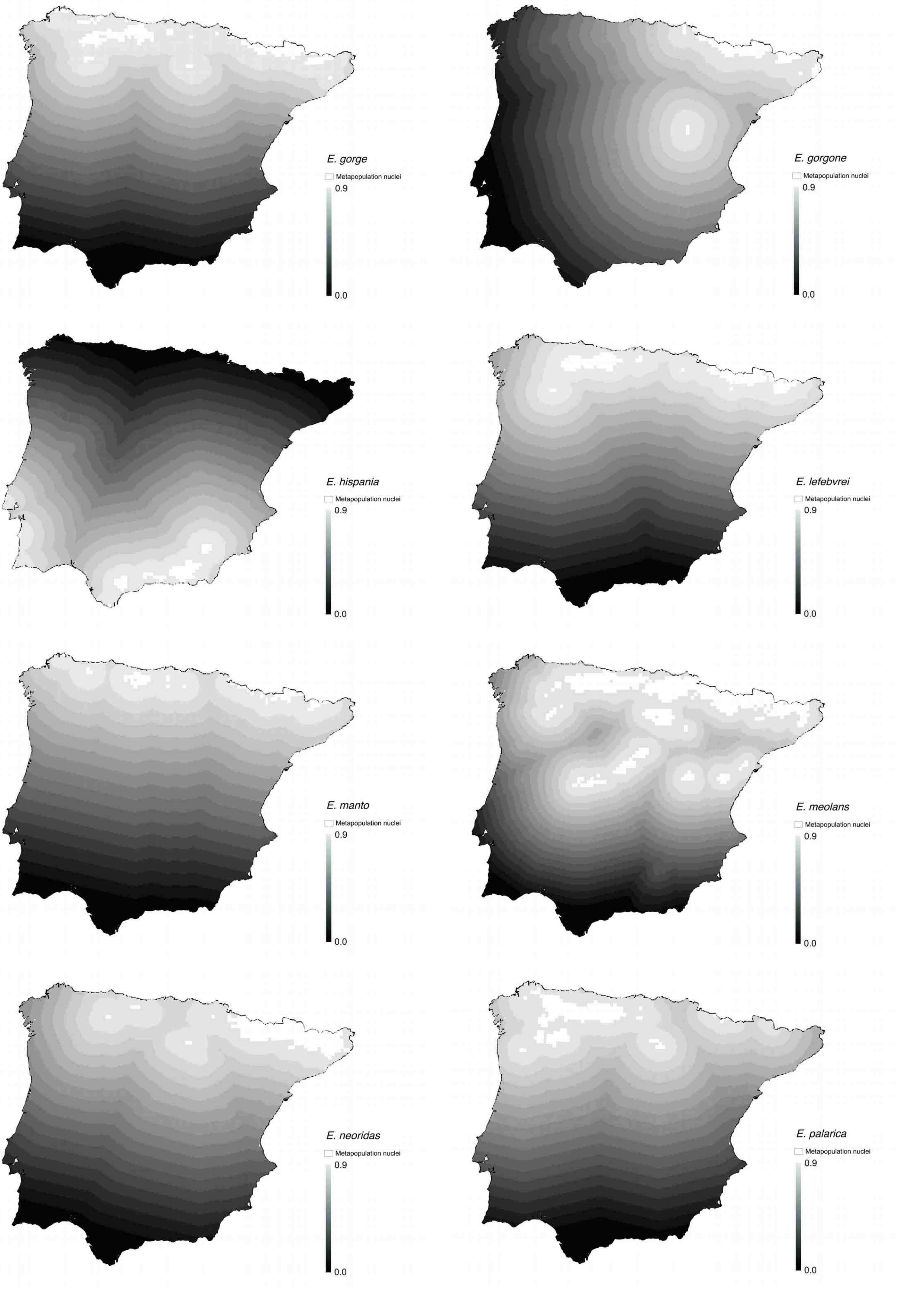

Family Nymphalidae

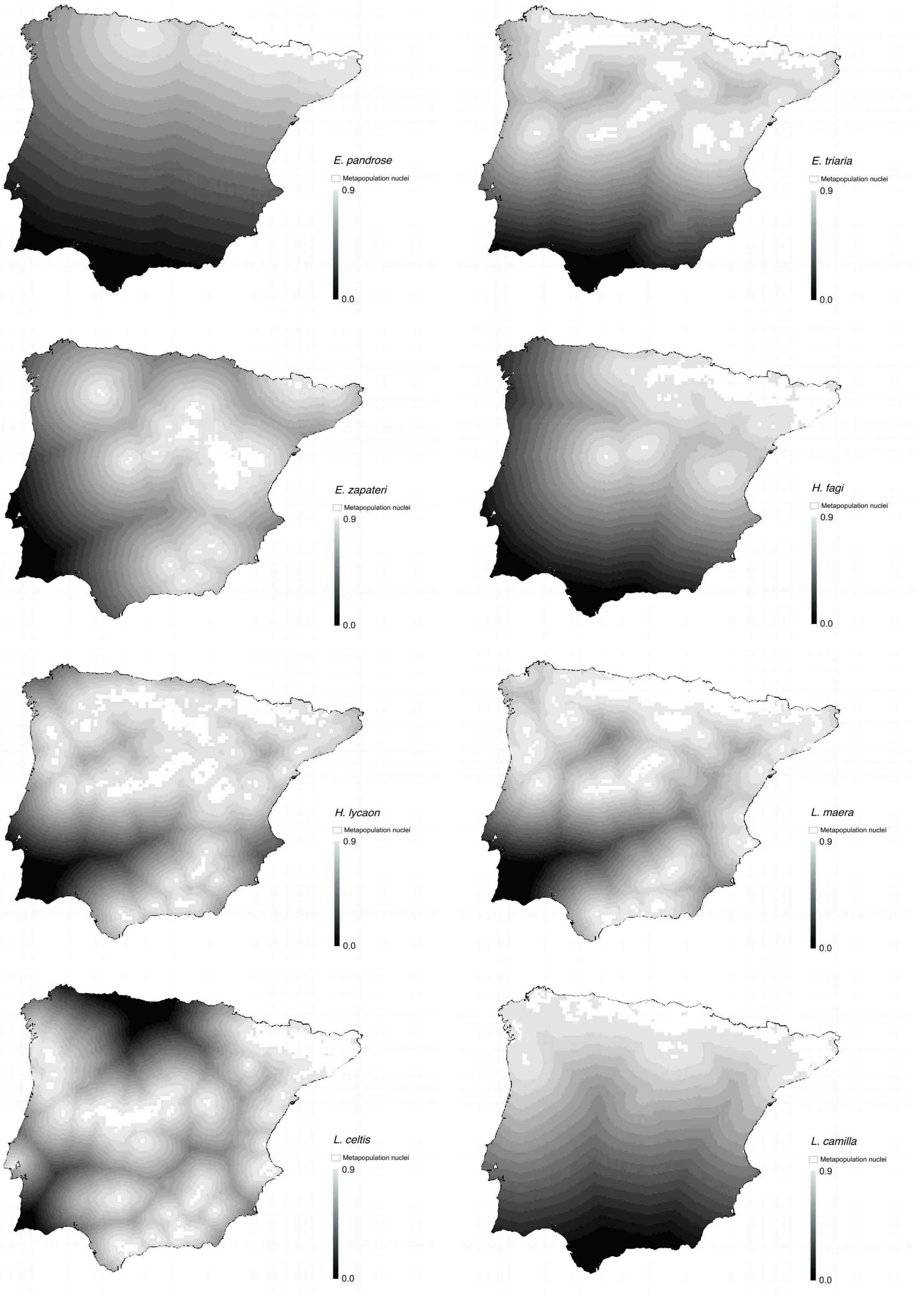

Family Nymphalidae

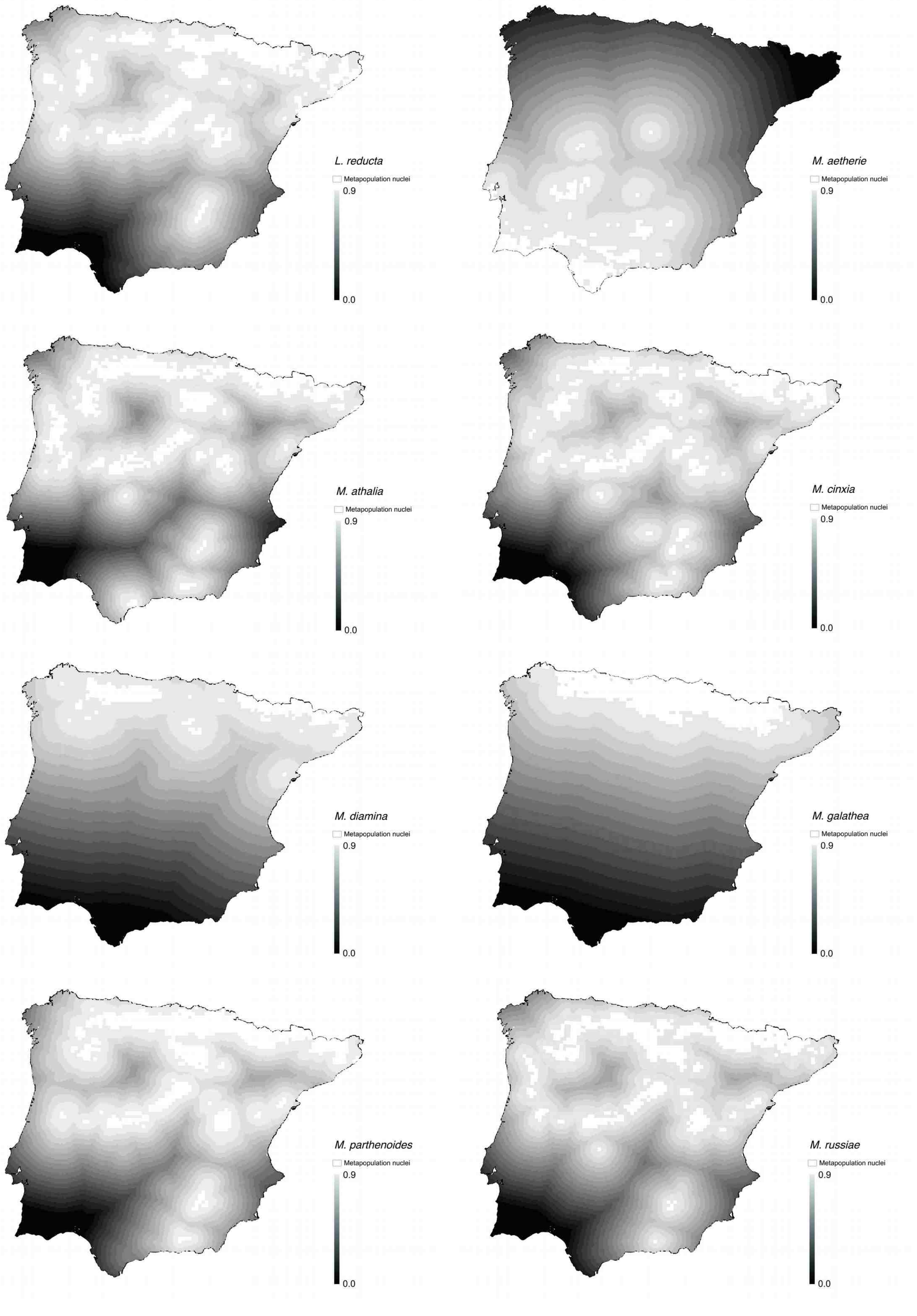

Family Nymphalidae

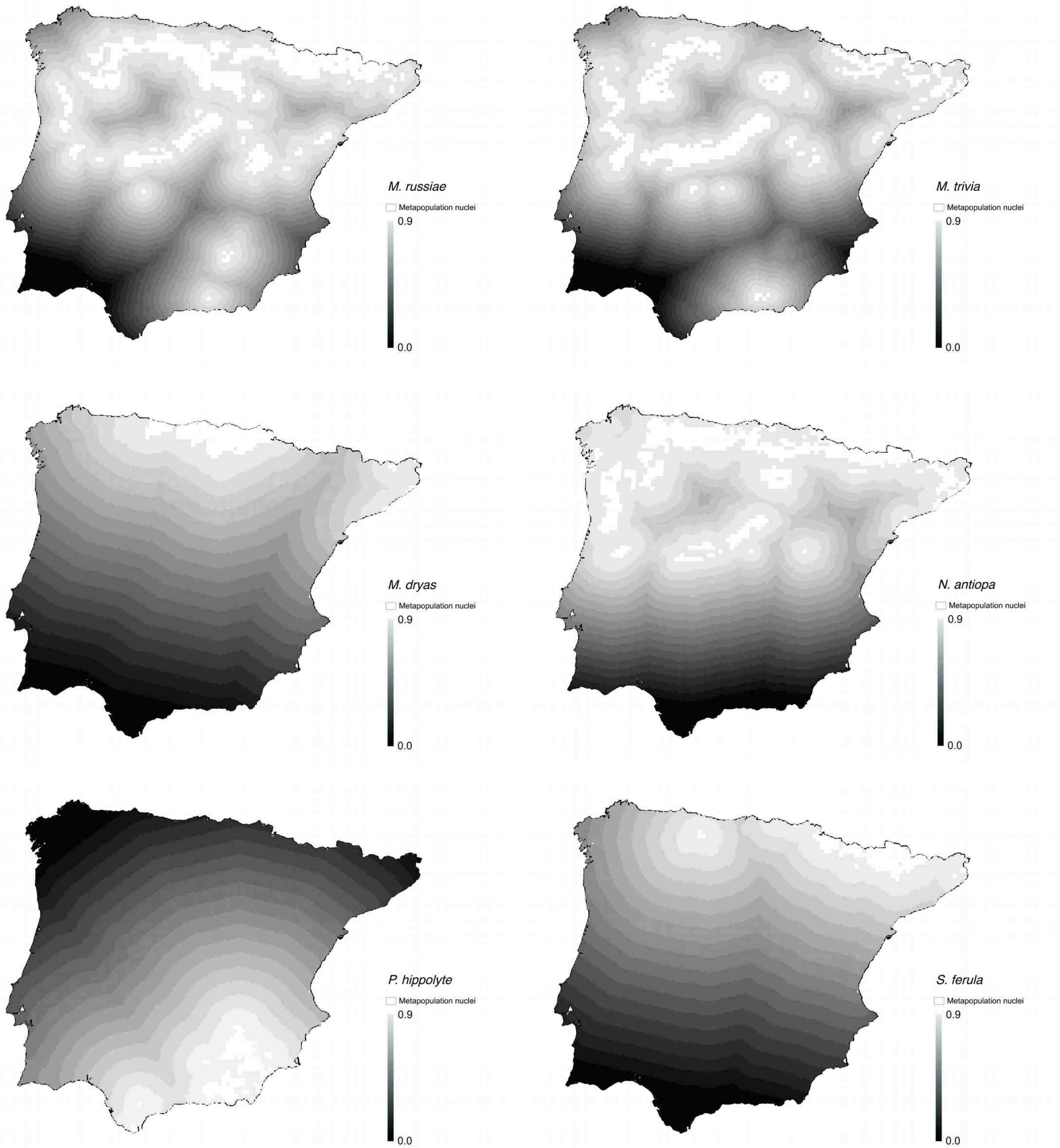

Family Lycaenidae

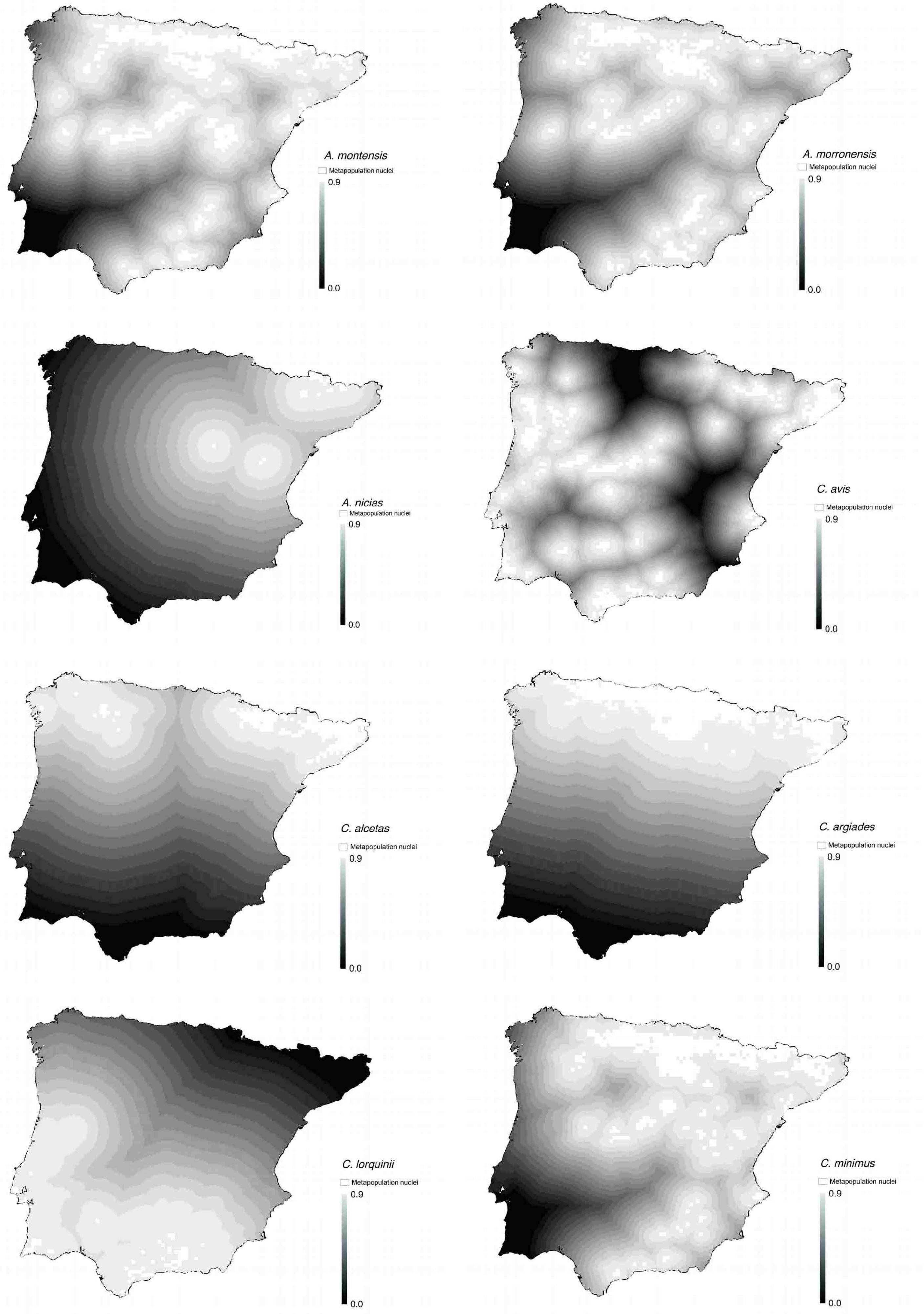

Family Lycaenidae

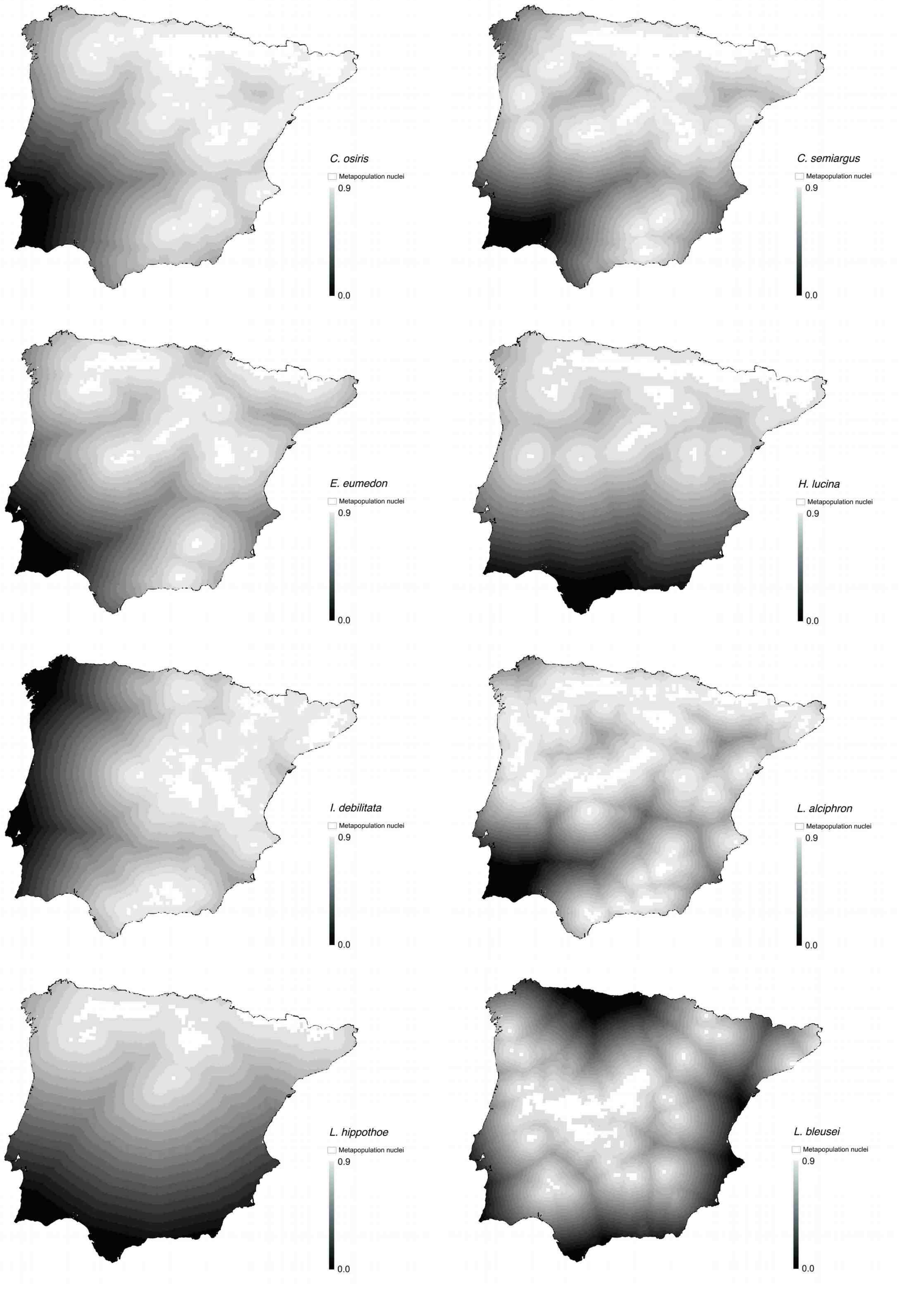

Family Lycaenidae

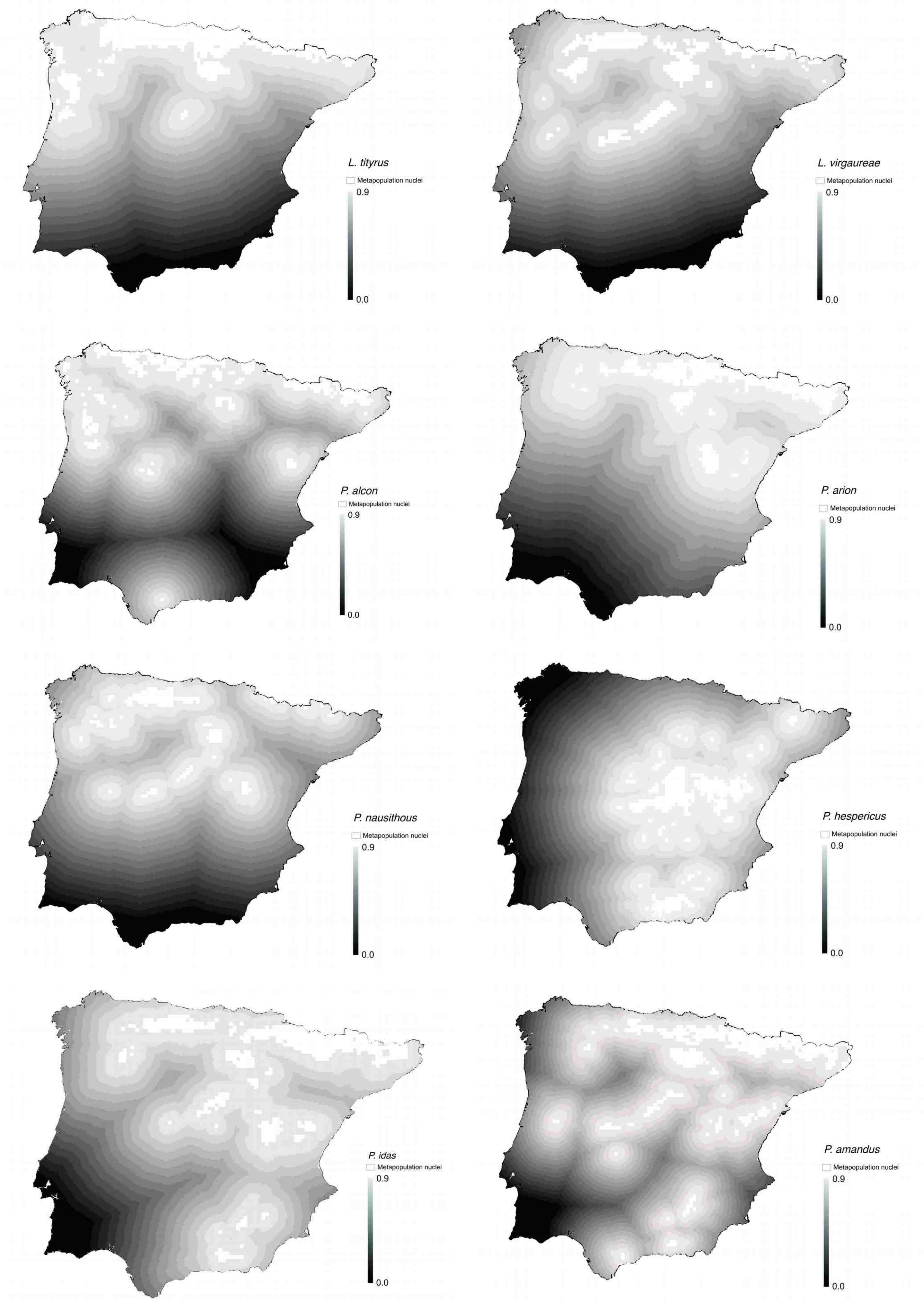

Family Lycaenidae

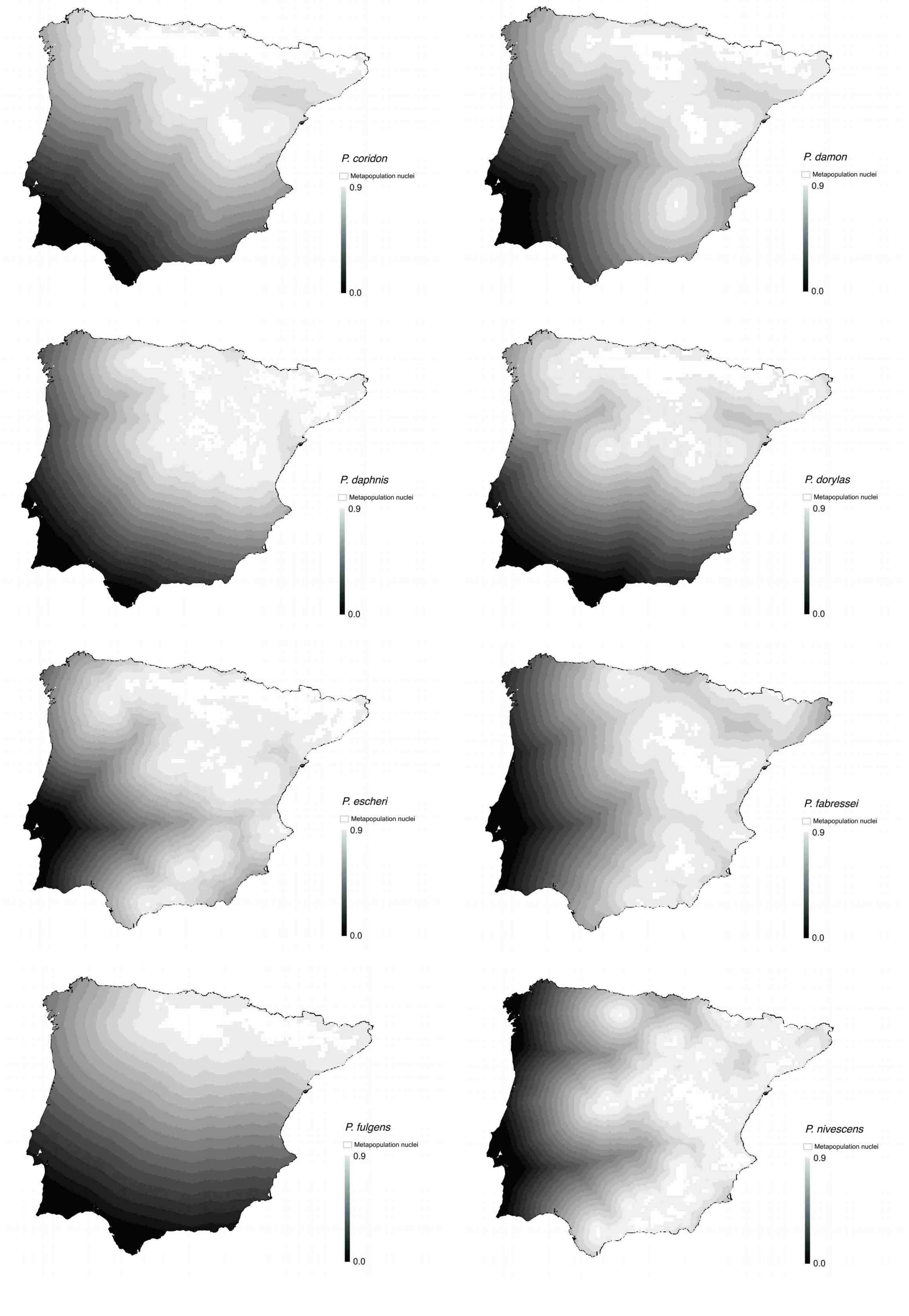

Family Lycaenidae

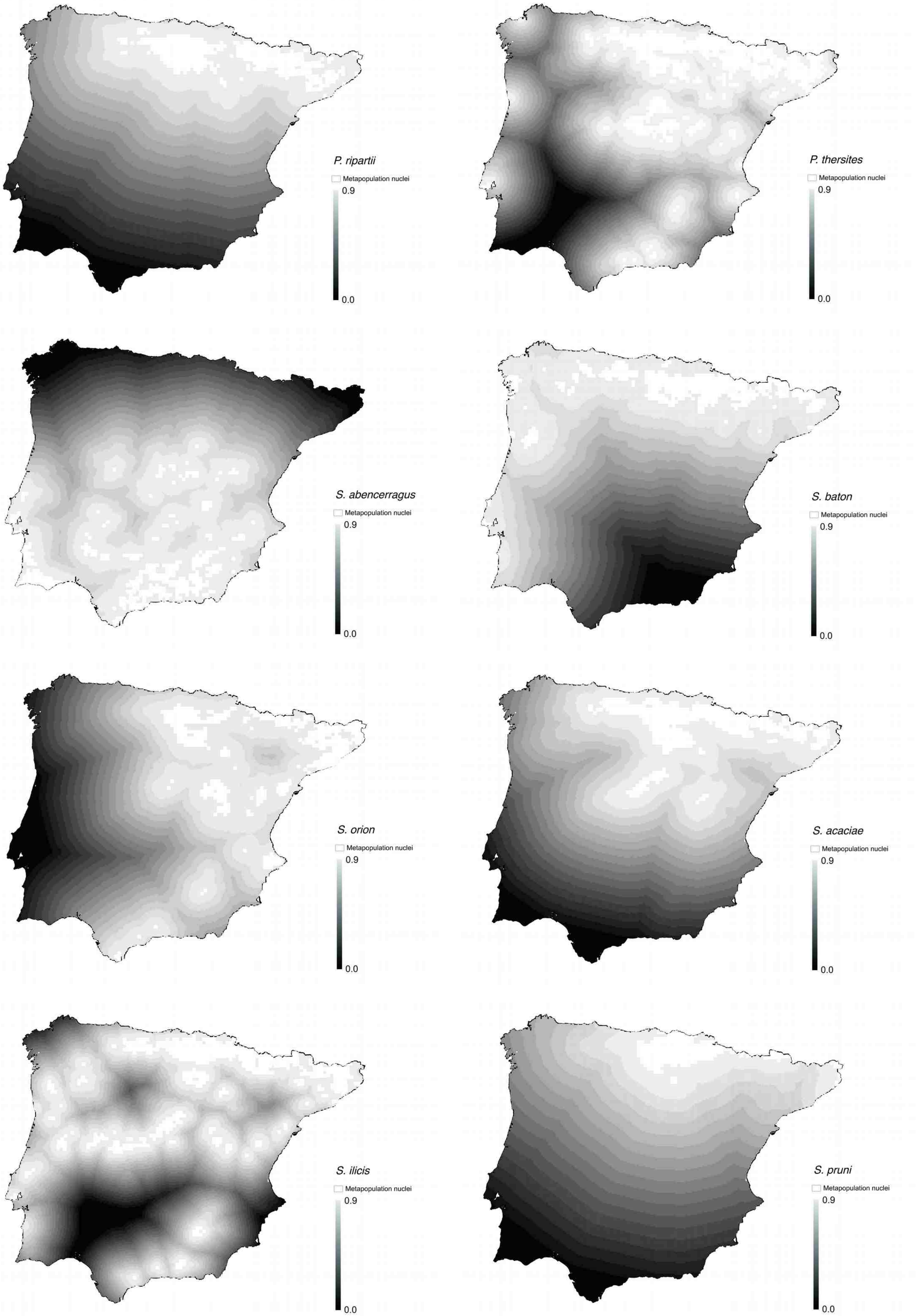

Family Lycaenidae

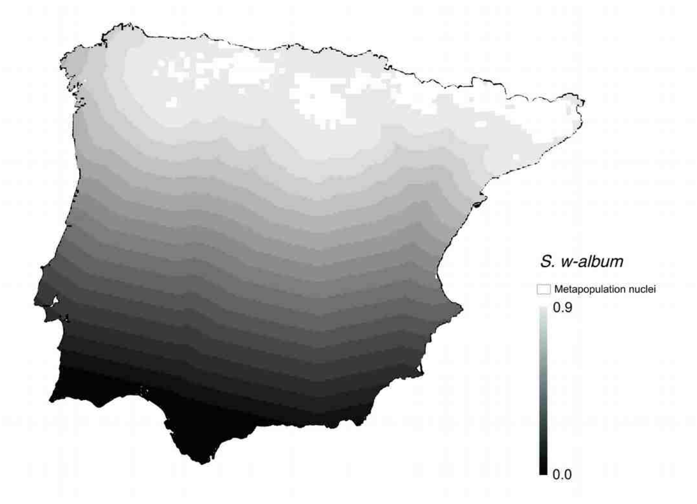

Figure S1. Maps of connectivity gradient between metapopulation nuclei (white plots). The matrix has been graded on a scale of eighteen levels of connectivity with a 0.05 connectivity range each (repre-sented in grayscale).
